# Discovery of the Hedgehog Pathway Inhibitor Pipinib that Targets PI4KIIIß

**DOI:** 10.1002/anie.201907632

**Published:** 2019-10-04

**Authors:** Lea Kremer, Elisabeth Hennes, Alexandra Brause, Andrei Ursu, Lucas Robke, Hideaki T. Matsubayashi, Yuta Nihongaki, Jana Flegel, Ivana Mejdrová, Jan Eickhoff, Matthias Baumann, Radim Nencka, Petra Janning, Susanne Kordes, Hans R. Schöler, Jared Sterneckert, Takanari Inoue, Slava Ziegler, Herbert Waldmann

**Affiliations:** ^1^ Department of Chemical Biology Max-Planck-Institute of Molecular Physiology Otto-Hahn-Straße 11 44227 Dortmund Germany; ^2^ Faculty of Chemistry and Chemical Biology Technical University Dortmund Otto-Hahn-Straße 6 44221 Dortmund Germany; ^3^ Department of Cell Biology Johns Hopkins University School of Medicine 855 N. Wolfe Street, 453 Rangos Baltimore MD 21205 USA; ^4^ Institute of Organic Chemistry and Biochemistry Flemingovo nam. 2 16610 Prague 6 Czech Republic; ^5^ Lead Discovery Center GmbH Otto-Hahn-Straße 15 44227 Dortmund Germany; ^6^ Department of Cell and Developmental Biology Max Planck Institute for Molecular Biomedicine Röntgenstr. 20 48149 Münster Germany; ^7^ Medical Faculty University of Münster Domagkstr. 3 48149 Münster Germany; ^8^ Technische Universität Dresden DFG-Research Center for Regenerative Therapies Dresden 01307 Dresden Germany; ^9^ Current address: Department of Chemistry The Scripps Research Institute 110 Scripps Way Jupiter FL 33458 USA

**Keywords:** biological activity, Hedgehog signaling, inhibitors, PI4KB

## Abstract

The Hedgehog (Hh) signaling pathway is crucial for vertebrate embryonic development, tissue homeostasis and regeneration. Hh signaling is upregulated in basal cell carcinoma and medulloblastoma and Hh pathway inhibitors targeting the Smoothened (SMO) protein are in clinical use. However, the signaling cascade is incompletely understood and novel druggable proteins in the pathway are in high demand. We describe the discovery of the Hh‐pathway modulator Pipinib by means of cell‐based screening. Target identification and validation revealed that Pipinib selectively inhibits phosphatidylinositol 4‐kinase IIIβ (PI4KB) and suppresses GLI‐mediated transcription and Hh target gene expression by impairing SMO translocation to the cilium. Therefore, inhibition of PI4KB and, consequently, reduction in phosphatidyl‐4‐phosphate levels may be considered an alternative approach to inhibit SMO function and thus, Hedgehog signaling.

## Graphical abstract

**Cell‐based screening** identifies a thieno[3,2]pyrimidine derivative as an inhibitor of Hedgehog signaling by selective inhibition of phosphatidylinositol 4‐kinase IIIβ (PI4KB), reduction of PI4P levels and prevention of Hedgehog‐mediated ciliary localization of Smoothened. Thus, PI4KB may be a relevant target protein for inhibition of Hedgehog signaling.

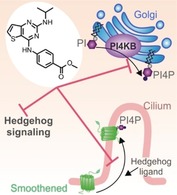

## Introduction

The Hedgehog (Hh) signaling pathway is essential during vertebrate embryonic development as well as for tissue homeostasis and regeneration in adults. Crucial events during pathway activation include binding of the Hh ligand to the Patched (PTC) receptor, transduction of the signal to the seven‐pass transmembrane protein Smoothened (SMO) and subsequent relocalization of SMO from intracellular vesicles to the cilium.^1^ This recruitment ultimately leads to translocation of the glioma‐associated oncogene 2 and 3 (GLI2/3) transcription factors to the nucleus and subsequent transcriptional activation of the pathway target genes, for example, *Gli1*, *Ptch1* and *Ptch2*.^2^

Hh signaling is upregulated in cancers of the skin (basal cell carcinoma, BCC)^3^ and the brain (medulloblastoma, MB).^4^ Pathway inhibitors targeting the SMO protein are in clinical use and under continuous development.^5^ However, therapeutic application of SMO inhibitors is hampered by development of resistance due to point mutations in SMO.^6^ Moreover, different mechanisms to activate Hh signaling in cancer cells exist that bypass SMO.^7^ Thus, identification of inhibitors that modulate proteins, not yet investigated as potential targets, is of major interest.^1^, ^6^ Identification of such compounds and their targets may be facilitated by target‐agnostic cellular assays and subsequent identification of the biological target. Due to their unbiased nature such assays may link bioactive compounds to unknown targets by means of appropriate readouts like monitoring differentiation processes/markers and indicative phenotypic changes.

We monitored Hh‐induced osteogenesis as a cell‐based screening for inhibitors of the Hh pathway. Subsequent target deconvolution and validation led to the discovery of the Hh pathway inhibitor Pipinib, which selectively targets PI4KB over the other three PI4‐kinases. Inhibition of PI4KB by Pipinib reduces intracellular PI4P levels, in particular at the Golgi apparatus, and impairs translocation of SMO to the cilium as well as subsequent pathway activation. Our results indicate that PI4KB may be a relevant target protein for pharmacological inhibition of Hh signaling.

## Results and Discussion

### Identification of a Novel Hh Signaling Pathway Inhibitor

**Figure 1:**
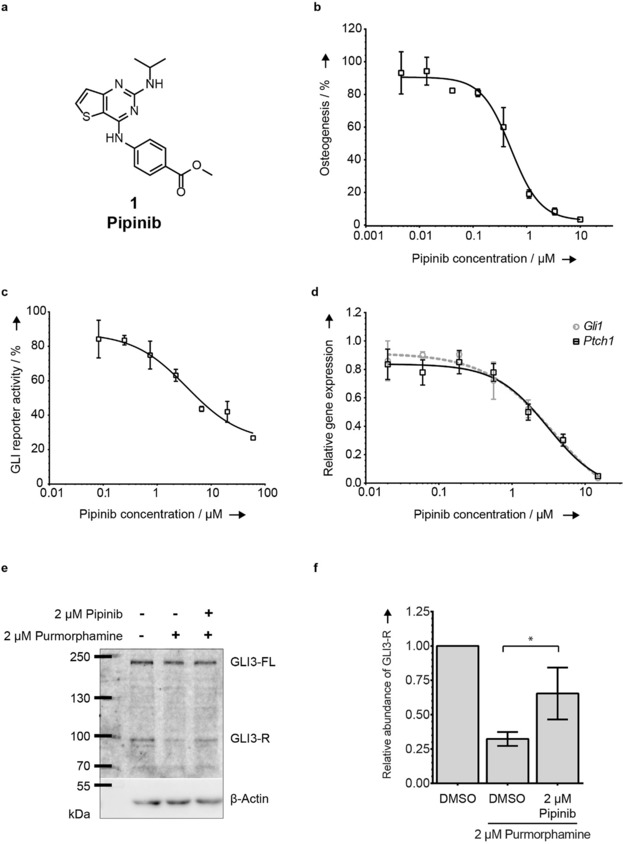
Identification of a novel Hh signaling pathway inhibitor. **a**. Structure of Pipinib (**1**). **b**. Osteoblast differentiation assay using C3H10T1/2 cells. DMSO‐Purmorphamine was set to 100 %. Data are mean values ± SD of three biological replicates. **c**. GLI reporter gene assay using Shh‐LIGHT2 cells. DMSO‐Purmorphamine was set to 100 %. Data are representative (mean values ± SD) of three biological replicates. **d**. Hh target gene expression. DMSO‐Purmorphamine was set to 1. Data are mean values ± SD of three biological replicates. **e**. GLI3‐FL and GLI3‐R levels. A representative blot of three biological replicates is shown. **f**. Quantification of GLI3‐R band intensities for three biological replicates. GLI3 band intensities were normalized to β‐actin band intensities and non‐activated lysates were set to 100 %. Statistical analysis was performed using an unpaired two‐tailed t‐test; *: *p*<0.05. Experimental details are given in the Supporting Information.

In order to identify novel inhibitors of Hh signaling, 336,639 compounds were subjected to an osteoblast differentiation assay that monitors differentiation of multipotent C3H10T1/2 mesenchymal progenitor cells to osteoblasts upon activation of the Hh pathway by treatment with the SMO agonist Purmorphamine.^10^, ^11^ Osteogenesis is detected through monitoring of alkaline phosphatase expression and activity (Figure S1 a, Supporting Information). The screen revealed a class of thieno[3,2]pyrimidine derivatives as potent inhibitors (Table S1, Supporting Information), with compound **1** (Figure 1 a) showing the highest activity. In addition to these compounds, several analogues were synthesized to further explore the correlation between activity and structure. The results shown in Table 1 indicate that for R^1^ the introduction of primary amines with acyclic (Table 1, entries **1**–**3** and **6**) or cyclic substituents (Table 1, entries **4**, **5**, **7**, **8**) and introduction of heteroatoms (Table 1, entries **6**, **7**) or of tertiary amines (Table 1, entries **9**–**11**) leads to reduction of bioactivity. In two cases, moderate cytotoxicity was observed (Table 1, entries **5** and **11**). Replacement of the methyl ester in R^2^ by a nitrile group (Table 1, entries **12**–**14**) and wider variation of the substitution pattern of the aromatic ring (Table S1) also yielded compounds with lower activity. Introduction of a substituent into the thiophene ring was not beneficial either (Table S1).

**Table 1:** Modulation of Purmorphamine‐induced osteogenesis^[a]^ and cell viability^[b]^ by thieno[3,2]pyrimidine derivatives. 
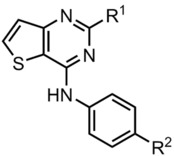

No.	R^1^	R^2^	Osteogenesis IC_50_ [μm]^[a]^	Viability IC_50_ [μm]^[b]^
1	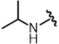	COOMe	0.6(±0.3)	inactive
2	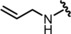	COOMe	2.9(±0.2)	>10
3	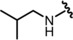	COOMe	3.4(±0.1)	>10
4	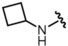	COOMe	3.8(±0.2)	>10
5	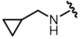	COOMe	3.4(±0.3)	8.0(±0.6)
6	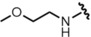	COOMe	4.9(±0.5)	inactive
7	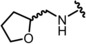	COOMe	6.8(±0.3)	inactive
8	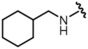	COOMe	7.7(±1.4)	>10
9	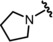	COOMe	2.1(±0.5)	>10
10	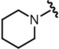	COOMe	3.0(±0.1)	>10
11	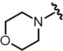	COOMe	2.9(±0.1)	6.8(±0.1)
12	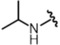	CN	2.4(±0.2)	inactive
13	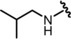	CN	6.5(±0.4)	inactive
14	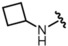	CN	4.0(±0.3)	inactive
15	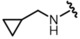	CN	3.8(±0.1)	>10
16	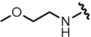	CN	6.3(±0.7)	inactive

[a] C3H10T1/2 cells were incubated with 1.5 μm Purmorphamine and the compounds or DMSO as a control for 96 h. The activity of alkaline phosphatase was assessed by means of a luminescence readout. Data are mean values of three independent experiments (*n*=3) ± SD. [b] Viability was measured in the same setting using a CellTiter Glo Kit (Promega). Values >10 μm were obtained when the inhibition achieved up to a concentration of 10 μm was not sufficient to calculate an IC_50_ value. A compound was considered as “inactive” if no toxicity was observed up to the maximum applied concentration of 10 μm.

The most active compound **1** (IC_50_=0.6±0.3 μm, Figure 1 b) was termed Pipinib and selected for further characterization. In an orthogonal assay, Pipinib inhibited GLI reporter gene activity in Sonic hedgehog (Shh)‐LIGHT2 cells (IC_50_=1.7±0.1 μm, Figure 1 c and S1 b) and expression of Hh target genes *Ptch1* and *Gli1* (IC_50_=3.1±0.9 μm and 4.1±1.6 μm, respectively, Figure 1 d). Treatment of NIH/3T3 cells with 2 μm Pipinib led to an increase of the truncated repressor form GLI3‐R (as compared to treatment with Purmorphamine) to which the full‐length protein is proteolytically converted upon pathway inactivation (Figure 1 e, quantification in Figure 1 f). These results show that Pipinib inhibits the Hh pathway upstream of GLI processing.

**Figure 2:**
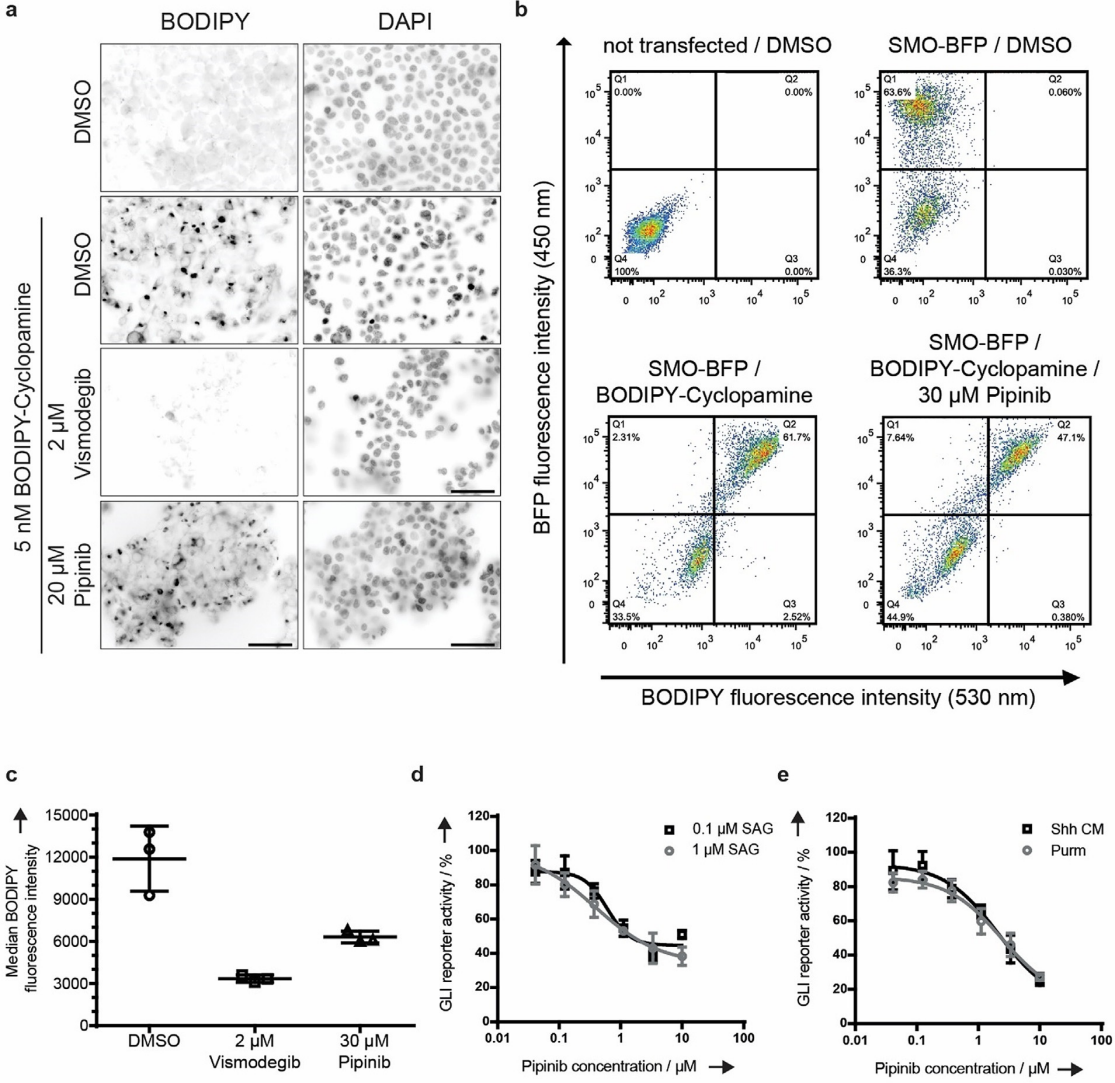
Pipinib does not modulate Hh signaling via SMO. **a**. SMO binding assay. Images are representative of three biological replicates. Scale bar: 50 μm. **b** and **c**. Quantitative SMO binding assay. **b**. Representative dot plots. **c**. Quantification of median BODIPY fluorescence intensity of three biological replicates. Only BFP‐positive, that is, SMO‐expressing, cells were considered for the calculation. Data are mean values of three biological replicates ± SD. **d** and **e**. GLI reporter gene assay using Shh‐LIGHT2 cells. Shh‐LIGHT2 cells were treated with different concentration of SAG (**d**) or 2 μm Purmorphamine or Shh conditioned medium (Shh CM) (**e**) and Pipinib or DMSO as a control for 48 h. Data are mean values ± SD of three biological replicates. Experimental details are given in the Supporting Information.

Numerous small molecule Hh pathway inhibitors bind to SMO and thereby inhibit Hh signaling.^12^ To assess direct binding of Pipinib to SMO, we monitored displacement^13^ of the BODIPY‐labelled SMO antagonist Cyclopamine, which binds to the heptahelical bundle of SMO.^14^ While the known SMO antagonist Vismodegib successfully competed with BODIPY‐Cyclopamine in SMO‐transfected HEK293T cells (detected as a decrease in BODIPY‐related cellular fluorescence, Figure 2 a), Pipinib did not displace BODIPY‐Cyclopamine from SMO at 10 and 20 μm (Figure 2 a and S2 a).

Quantitative analysis of BODIPY‐related fluorescence in live cells expressing BFP‐SMO via flow cytometry indicated partial displacement of BODIPY‐Cyclopamine by Pipinib, (Figure 2 b,c and S2 b,c). To further address the putative SMO targeting by Pipinib, we employed inducers of Hh signaling with different modes of action, i.e., Shh, which binds to PTC1 and acts upstream of SMO and SAG, which is a SMO agonist. Compounds that bind to the heptahelical bundle in SMO, which is targeted by most SMO modulators like Purmorphamine, SAG, and Vismodegib, should display weaker potency when Hh signaling is activated with high vs. low concentration of SAG (e.g., Vismodegib, Figure S3 a) or Purmorphamine vs. Shh (e.g., Vismodegib, Figure S3 b).^15^ In contrast, Pipinib, similar to the GLI inhibitor GANT61, retained similar potency upon activation of the Hh pathway with 1 μm SAG (Figure 2 d and S3 c) or Purmorphamine (Figure 2 e, S1 c, and S3 d) as compared to stimulation with 0.1 μm SAG or Shh, respectively. Thus, whereas Pipinib may bind to SMO at high concentrations, it most likely does not act via inhibition of SMO at lower concentration.

### Pipinib is an Inhibitor of PI4KB

Inspection of the chemotype9a, 16 representative of Pipinib suggested that the compound might be a kinase inhibitor. Indeed, thienopyrimidine derivatives with such activity have been reported to target SYK and Protein kinase D1 (PRKD1).^17^ Investigation of binding to, or inhibition of 394 wildtype and 66 mutated kinases by 10 μm Pipinib (Table S2) revealed eight potential targets (Table S3), of which phosphatidylinositol 4‐kinase IIIβ (PI4KB) showed the highest inhibition (76±2 %). Pipinib did not inhibit the enzymatic activity of Syk and PRKD1 and PRKD2 (Table S2). Of the potential targets, GRK7 was excluded because mouse cells were used in the osteoblast differentiation assay and mouse orthologs of GRK7 do not exist (https://www.genecards.org;^18^ GCID: GC03P141778). Although we cannot rule out impairment of a non‐enzymatic function, MAPK8 and MYLK4 were excluded as well because, in contrast to binding, inhibition of the enzymatic activity was <50 % (Table S4). siRNA knockdown or employment of structurally unrelated kinase inhibitors devalidated TTK, GAK, PIP5K1C and PIK3C2G with respect to Hh signaling (Figure S4).

**Figure 3:**
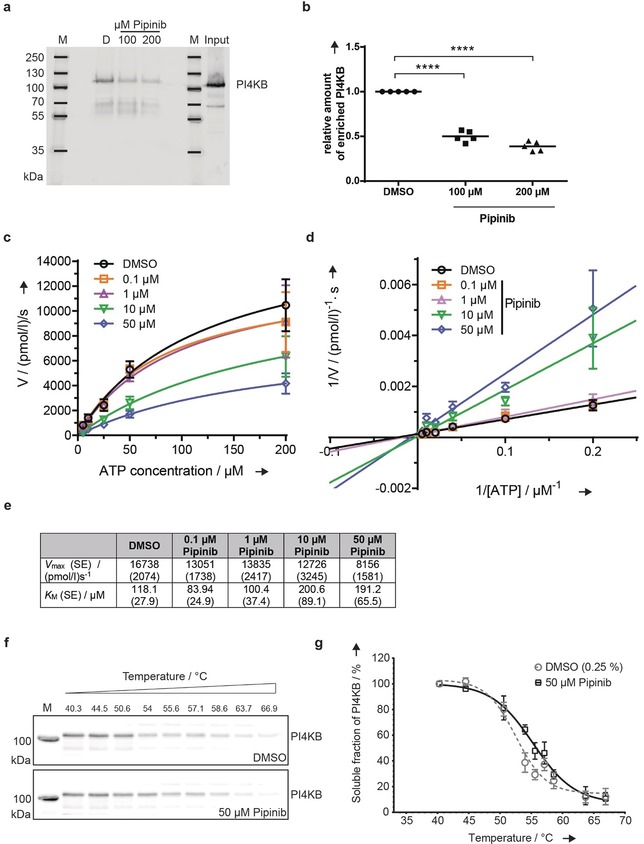
Pipinib is an inhibitor of PI4KB. **a**. Immunoblot readout for PI4KB after ActiveX affinity chromatography. A representative blot is shown (five independent experiments). **b**. Quantification of PI4KB that was enriched by the desthiobiotin probe (see **a**, data from five biological replicates). ****: *p*<0.0001 (unpaired t‐test with Welch's correction). **c**–**e**. Mode of PI4KB inhibition. Different concentrations of Pipinib were titrated against different ATP concentrations in an in vitro kinase activity assay. Michalelis‐Menten plot (**c**) and Lineweaver–Burk plot (**d**) of the obtained velocity against the substrate concentration are shown. Data are mean values ± SD (three independent experiments). **e**. *V*_max_ and *K*_m_ values obtained from **c**. SE: standard error. **f**. Cellular thermal shift assay. The blot is representative of three biological replicates. **g**. Quantification of PI4KB band intensities from three repetitions of the experiment shown in **f**. Data were normalized to the 40.3 °C band intensity (100 %) and are mean values ± SD (three biological replicates). Experimental details are given in the Supporting Information.

Monitoring of ATP displacement from kinase active sites by an inhibitor, here Pipinib, by means of an affinity enrichment in cell lysates (ActiveX ATP probe displacement assay, Figure S5 a) identified PI4KB as the only target protein of Pipinib (Figure 3 a,b and S5 b–S5d). Pipinib inhibits PI4KB (IC_50_=2.2±0.8 μm) but not the isoenzymes PI4KA (phosphatidylinositol 4‐kinase IIIα), PI4K2A (phosphatidylinositol 4‐kinase IIα) or PI4K2B (phosphatidylinositol 4‐kinase IIβ) up to a concentration of 50 μm (Figure S5 e). Kinetic measurements and analysis demonstrated that Pipinib is an ATP‐competitive inhibitor of PI4KB activity (Figure 3 c–e). Target engagement was confirmed by means of a cellular thermal shift assay (CETSA)^19^ that showed an increase of the melting temperature for PI4KB by 2.8±0.6 °C upon exposure to Pipinib (Figure 3 f, quantification in Figure 3 g). Identification of PI4KB as target of Pipinib in the context of Hh pathway inhibition is in accordance with earlier findings suggesting a role for PI4P, i.e., the product of phosphorylation of PIP by PI4KB, in the regulation of SMO activity^1^, ^20^ and of protein trafficking to the cell membrane.^21^

### Pipinib Reduces PI4P Levels in Cellulo

**Figure 4:**
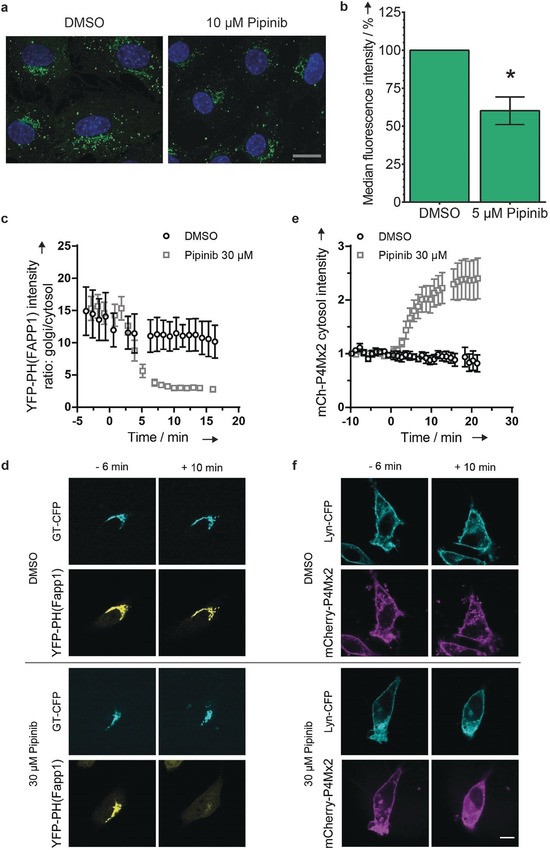
Pipinib reduces PI4P levels in NIH/3T3 cells. **a**. Qualitative assessment of intracellular PI4P levels. Cells were incubated with the compounds for 6 h and were fixed and stained with an antibody against PI4P (green) and DAPI (nucleus, blue). Zoom‐in of representative images (three biological replicates, scale bar=20 μm), full size images are available in Figure S6. **b**. Quantitative assessment of intracellular PI4P levels. Cells were treated with the compounds for 48 h. Data are mean values ± SD of three biological replicates. *: *p*<0.05 (unpaired t‐test with Welch's correction). **c**. Live‐cell tracing of PI4P with PH(FAPP1). The graph shows the ratio of Golgi to cytosol intensity of the YFP‐PH(FAPP1) sensor. Data are mean values ± SEM (*N*=8 for each). **d**. Representative images show cells 6 min before and 10 min after addition of Pipinib and DMSO, respectively. **e**. Live‐cell tracing of PI4P with P4Mx2. The graph shows the intensity of cytosolic mCherry‐P4Mx2. **f**. Representative images show cells 6 min before and 10 min after addition of Pipinib and DMSO, respectively. Scale bar: 10 μm (for the full movies, see Supporting Information, Movies 1–4). Experimental details are given in the Supporting Information.

Visualization of cellular PI4P levels with a validated antibody^22^ revealed a reduction of PI4P levels 6 h after treatment of NIH/3T3 cells with Pipinib (Figure 4 a and S6). Prolonged treatment with 5 μm Pipinib for 48 h led to a decrease of PI4P levels to ca. 60 % (Figure 4 b), as measured by means of flow cytometry. The dynamics of intracellular PI4P pools was monitored employing a YFP‐PH(FAPP1) PI4P sensor^23^ or a mCh‐P4Mx2 PI4P sensor,^24^ which both recognize PI4P via the PH domain of the FAPP1 protein or the P4M domain from *L. pneumophila* SidM,^23^, ^24^ qualifying these fusion proteins as sensors for PI4P.

The sensors were applied in combination with a Golgi marker (β1,4‐galactosyltransferase fused to CFP, GT‐CFP^25^) or a plasma membrane marker (Lyn kinase fused to CFP, Lyn‐CFP^26^). Addition of 30 μm Pipinib to NIH/3T3 cells transfected with YFP‐PH(FAPP1) and GT‐CFP led to reduction of the Golgi intensity of YFP‐PH(FAPP1) within 10 min (Figure 4 c,d). In addition, treatment of NIH/3T3 cells transfected with mCh‐P4Mx2 and Lyn‐CFP led to an increase of mCh‐P4Mx2 in the cytosol (Figure 4 e,f). PI4KB is located at the Golgi, while the isoenzyme PI4KA localizes to the plasma membrane.^27^ Since Pipinib selectively inhibits PI4KB, the live‐cell imaging results indicate that reduction of PI4P levels at the Golgi is directly due to enzyme inhibition.

### Pipinib Impairs Ciliary Localization of SMO

**Figure 5:**
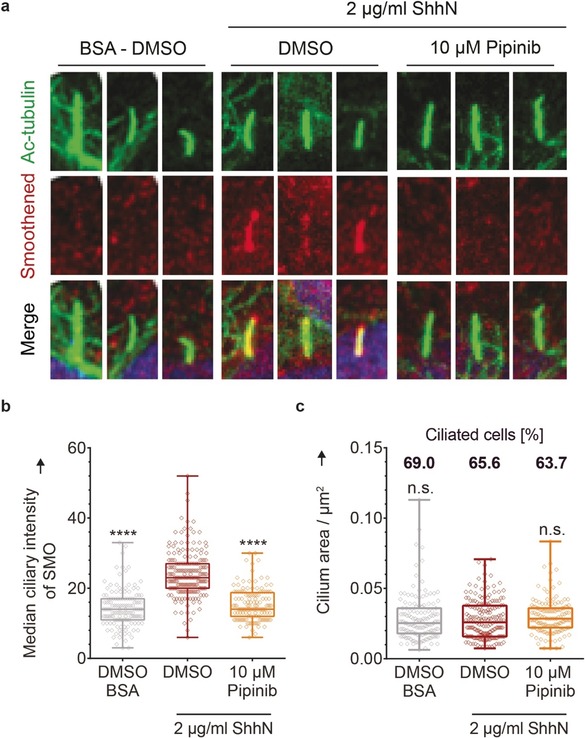
Pipinib impairs ciliary localization of SMO in NIH/3T3 cells. **a**. Ciliary localization of SMO. Cilia were stained with an antibody against acetylated tubulin (Ac‐tubulin, green), SMO was stained with an anti‐SMO antibody (red) and the nucleus was marked with DAPI (blue). Images are representative of three biological replicates. Zoom‐in on examplary cilia is shown. Full images are provided in Figure S7. **b**. Quantification of ciliary localization of SMO. More than 100 cilia of a representative experiment were analyzed for the intensity of the anti‐SMO antibody (see Figure 5 a). Each data point represents the intensity value of a single cilium. Statistical significance was assessed using an unpaired t‐test with a confidence level of 95 % (****=*p*<0.0001). **c**. Quantification of cilium area and percentage of ciliated cells. More than 100 cilia of a representative experiment were analyzed for the ciliary area defined by Ac‐tubulin staining (three independent experiments). Each data point represents the area count of a single cilium. Statistical significance was assessed using an unpaired t‐test with a confidence level of 95 % (n.s.=*p*>0.05). The percentage of ciliation was assesed by dividing the number of cilia (ac‐tubulin staining) by the number of cells (DAPI staining) in each picture (three independent experiments). Experimental details are given in the Supporting Information.

PI4P was reported to bind to SMO and to promote its phosphorylation and thus ciliary accumulation.^20^ Pipinib inhibits PI4KB and reduces PI4P levels suggesting that this compound may also impair the proper localization of SMO upon pathway activation. To investigate whether Pipinib affects SMO trafficking, we detected SMO and acetylated tubulin as a ciliary marker^28^ in ciliated cells. Pipinib induced a significant decrease of ciliary SMO after activation of Hedgehog signaling (Figure 5 a, quantification in Figure 5 b, see also Figure S7), while neither ciliary size nor ciliogenesis were affected (Figure 5 c and S7). Thus, Pipinib prevents translocation of SMO to the cilium, which may be attributed to reduced PI4P levels and/or binding to SMO, thereby inhibiting Hedgehog signaling.

### Validation of PI4KB as a Positive Regulator of Hh Signaling and Effector of Pipinib

**Figure 6:**
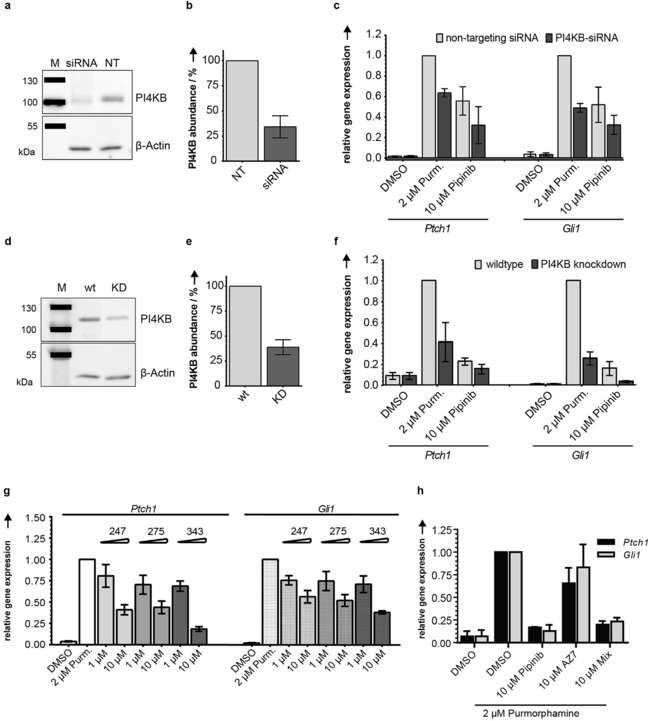
Validation of PI4KB as a positive regulator of Hh signaling and effector of Pipinib in NIH/3T3 cells. **a**. siRNA knockdown efficiency. Representative blot of PI4KB levels. **b**. Quantification of band intensities from three repetitions of the experiment shown in **a**. PI4KB band intensities were normalized to β‐actin band intensities and values for cells that were treated with non‐targeting siRNA (NT) were set to 100 %. **c**. siRNA‐mediated knockdown of PI4KB. Data are mean values ± SD of three biological replicates. **d**. CRISPR‐Cas9‐mediated knockdown (KD) efficiency. Representative blot of PI4KB levels. **e**. Quantification of band intensities from three repetitions of the experiment shown in **d**. PI4KB band intensities were normalized to β‐actin band intensities and lysates from parental NIH/3T3 cells (wt) were set to 100 %. **f**. CRISPR‐Cas9‐mediated knockdown of PI4KB.Cells were treated with the compounds for 48 h. Data are mean values ± SD of three biological replicates. **g**. Influence of PI4KB inhibitors on Hh target gene expression. Data are mean values ± SD of three biological replicates. **h**. Influence of PI4KA inhibitor AZ7 on Hh target gene expression. NIH/3T3 cells were treated as in G, using AZ7 instead of the PI4KB inhibitors as well as an equimolar mixture of Pipinib and AZ7 (Mix). Data are mean values ± SD of three independent experiments. Experimental details are given in the Supporting Information.

Downregulation of PI4KB by means of siRNA (Figure 6 a, quantification in Figure 6 b; knockdown efficiency 69 %) led to reduced expression of *Ptch1* and *Gli1* target genes after pathway activation with Purmorphamine (Figure 6 c), which is in accordance with earlier findings.^29^ Additional treatment with Pipinib led to further reduction of gene expression (Figure 6 c), and, accordingly, a shift of IC_50_ values for inhibition of *Ptch1* and *Gli1* expression from 5.2±2.5 μm and 7.3±2.4 μm to 1.6±0.3 μm and 1.6±0.5 μm, respectively, in the presence of PI4KB targeting siRNA (see also Figure S8). Using the CRISPR‐Cas9 system, we attempted to disrupt the *Pi4kb* gene. Unfortunately, *Pi4kb* knockout cells could not be generated, most likely because PI4KB is an essential gene.^30^ However, the CRISPR‐Cas9 approach led to depletion of PI4KB. By analogy to siRNA‐mediated knockdown, depletion of PI4KB with the CRISPR‐Cas9 system (efficiency 61.6±5.3 %, Figure 6 d, quantification in Figure 6 e) led to reduced expression of *Ptch1* and *Gli1* after pathway stimulation with Purmorphamine (Figure 6 f). Application of known PI4KB inhibitors MI‐247, 275 and 343 with unrelated chemotypes^31^ (for structures see Figure S9 a) dose dependently inhibited *Ptch1* and *Gli1* expression (Figure 6 g), reduced the GLI responsive reporter activity (Figure S9 b) and the ciliary localization of SMO ( Figure S9 c), thereby further confirming the results obtained with Pipinib. These findings show that Pipinib targets and inhibits PI4KB and thereby leads to impaired Hh signaling.

### PI4KB is the Major PI4‐Kinase Required for Hh Signaling

Depletion of both, PI4KB and PI4KA has been reported to reduce GLI reporter gene expression, whereas depletion of PI4K2A or PI4K2B did not affect Hh signaling.^29^ In order to determine whether both isoenzymes are positive regulators of Hh signaling, we investigated modulation of Hh target gene expression by the selective PI4KA inhibitor AZ7.^32^ While treatment of NIH/3T3 cells with 10 μm Pipinib led to reduction of *Ptch1* and *Gli1* expression by 83 and 87 %, application of the biochemically ca. 1000‐fold more potent PI4KA inhibitor (IC_50_=6.3 nm) at the same concentration reduced gene expression only by 44 and 17 % (Figure 6 h). In addition, application of an equimolar mixture of Pipinib and AZ7 did not show an additive effect suggesting that PI4KB is at least the major PI4‐kinase positively regulating Hh signaling.

## Conclusion

Target‐agnostic cell‐based assessment of bioactivity for small molecules holds promise to link biological processes to yet unexplored biomolecules, in particular proteins. We have identified the thieno[3,2]pyrimidine derivative Pipinib as a novel selective inhibitor of PI4KB and show that small‐molecule inhibition of this lipid kinase leads to Hh pathway inhibition. We demonstrate that Pipinib reduces ciliary localization of SMO, which may be attributed to inhibition of PI4KB and/or the binding to SMO. We validate PI4KB as a relevant Hh pathway component via siRNA knockdown and employment of additional PI4KB inhibitors with a structurally unrelated chemotype.

Phosphatidylinositol‐4‐phosphate (PI4P) was proposed as small‐molecule mediator of signal propagation from PTC to SMO. We demonstrate that phosphatidylinositol 4‐kinase IIIβ (PI4KB) generates PI4P that is relevant for Hh signaling. The mechanism by which the Hh signal is transduced from PTC to SMO and by which PTC controls SMO in mammalian cells has been described as “the major mystery of the Hh field”.^1^ Since both receptors do not physically interact, it has been suggested that the inhibitory effect of PTC on SMO is mediated via an endogenous small molecule modulator whose abundance is controlled directly or indirectly by PTC.^1^ Several endogenous modulators have been postulated, for example, oxysterols,^33^ cholesterol^34^ or budesonide^35^ but final proof and a link to PTC is still missing.

In *Drosophila*, loss‐of‐function mutations have shown that the lipid kinase SST4, the ortholog of mammalian PI4KA, is necessary for SMO translocation to the membrane upon Hh pathway activation.^29^ In addition, Hh pathway activity is reduced in mouse cells after depletion of PI4KA and PI4KB but not after knockdown of PI4K2A or PI4K2B.^29^ Recent findings by Jiang et al. confirmed the link between PI4P and Hedgehog signaling.^20^ It was shown that the phosphoinositide binds to the C‐terminal tail of SMO in vitro, which induces a conformational change that promotes SMO migration to the cilium and that Hh signaling regulates PI4P levels through PTC.^20^ However, the mechanism underlying this possible modulation is unknown. In general, PI4P is generated by four isoenzymes that have distinct cellular localizations and are differently regulated.^27^ PI4P is necessary for membrane trafficking, serves as precursor for various lipid signaling molecules and can be a signaling molecule on its own.21b, 36 PI4P generation at the Golgi, e.g., by PI4KB, modulates the lipid composition of more distal membranes like the plasma membrane, suggesting that inhibition of differently localized PI4‐kinases may have widespread effects throughout the whole cell.^36^

Our results demonstrate the relevance of PI4KB for Hh signaling as previously indicated by genetic evidence.^29^ PI4KB is localized at the Golgi where it generates PI4P that is required for vesicular trafficking to the plasma membrane.^27^ Pipinib also inhibits Hh signaling when the pathway was activated by the SMO agonist Purmorphamine.^13^, ^15^ Since this mode of pathway activation circumvents PTC, it is unlikely that PTC regulates PI4KB. A possible explanation could be that PI4P is one of several SMO ligands. It has been suggested that SMO is controlled by two endogenous ligands, i.e., one Shh‐independent ligand that targets the cysteine‐rich domain (CRD), and one ligand regulated by PTC that binds to the transmembrane (TM) core of SMO.6c The CRD ligand would prime SMO for activation and would act in synergism with the second ligand—both steps would be required for full pathway activation.6c PI4P binds to the cytoplasmic tail of SMO^20^ and could likewise fulfill the role of a priming ligand. Purmorphamine, which binds to the TM domain, might need PI4P to fully activate Hh signaling. Jiang et al., however, have shown that PI4P alone activates Hh signaling and induces translocation of SMO to the cilium.^20^ Since signaling in cells stimulated with the SMO agonist Purmorphamine was inhibited by treatment with Pipinib or by siRNA‐mediated depletion of PI4KB, we propose that PI4KB activity is not controlled by Hh signaling. This is supported by the fact that PI4P is required for general cellular processes like overall membrane trafficking and that there is no indication for PTC‐mediated regulation of PI4KB activity.

## Conflict of interest

The authors declare no conflict of interest.

## Supporting information

As a service to our authors and readers, this journal provides supporting information supplied by the authors. Such materials are peer reviewed and may be re‐organized for online delivery, but are not copy‐edited or typeset. Technical support issues arising from supporting information (other than missing files) should be addressed to the authors.

Supplementary

Supplementary

Supplementary

Supplementary

Supplementary
