# Supplementary material for: Discovery of the Hedgehog Pathway Inhibitor Pipinib that Targets PI4KIIIß
Source: Angew Chem Int Ed Engl. 2019 Oct 4;58(46):16617–28. doi: 10.1002/anie.201907632 (PMC6900058; doi:10.1002/anie.201907632)
Supplement: Supplementary file 1 — Supplementary [file ANIE-58-16617-s001.pdf]

Angewandte Chemie
Eine Zeitschrift der Gesellschaft Deutscher Chemiker

Supporting Information

Discovery of the Hedgehog Pathway Inhibitor Pipinib that Targets PI4KIIIß

*Lea Kremer, Elisabeth Hennes, Alexandra Brause, Andrei Ursu, Lucas Robke, Hideaki T. Matsubayashi, Yuta Nihongaki, Jana Flegel, Ivana Mejdrová, Jan Eickhoff, Matthias Baumann, Radim Nencka, Petra Janning, Susanne Kordes, Hans R. Schöler, Jared Sterneckert, Takanari Inoue, Slava Ziegler, and Herbert Waldmann**

anie_201907632_sm_miscellaneous_information.pdf
anie_201907632_sm_Movie_S1.avi
anie_201907632_sm_Movie_S2.avi
anie_201907632_sm_Movie_S3.avi
anie_201907632_sm_Movie_S4.avi

Author Contributions

H.W. conceived the project. S.Z., L.K. and T.I. designed the experiments. L.K. performed the biological experiments (lead). E.H. performed the PI4KB siRNA experiments and A.B. performed the GLI3 immunoblots, the CETSA experiments and other siRNA experiments. J.F. performed SMO localization and binding assays and reporter gene assays. A.U. and L.R. performed the chemical synthesis. H.T.M. performed the PI4P sensor experiments. S.K., H.R.S., J.S., M.B. and J.E. derived the target hypothesis. R.N. provided the published PI4KB inhibitors that have been synthesized by I.M. P.J. supervised the MS/MS measurements and performed the associated data analysis. Y.N. and T.I. analyzed the results of the PI4P sensor experiments. H.W., S.Z. and L.K. interpreted all experimental data. H.W., S.Z., T.I. and L.K. wrote the manuscript. All authors commented on the manuscript.

Table of Contents

1. Supporting Tables 3
2. Supporting Figures 9
3. Supporting Movies 20
4. Experimental procedures – Biology 21
5. Experimental procedures - Chemical synthesis 37
   5.1. PI4KA inhibitor 40
   5.2. PIK3C2G inhibitors 40
   5.3. Derivatives of Pipinib (entry 1) 43
   5.5. NMR Spectra 52
6. References 60

1. Supporting Tables

Supporting Table S1 (related to Table 1). Results of the initial osteogenesis screen[a].

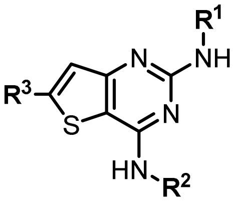

No	R^1	R^2	R^3	Osteogenesis IC_{50} (µM)
**1**	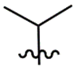	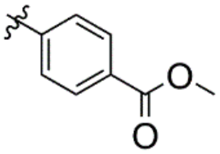	H	0.93
**2**	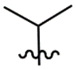	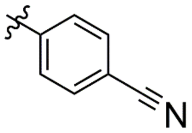	H	5.47
**3**	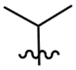	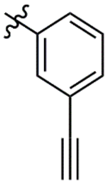	H	5.62
**4**	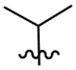	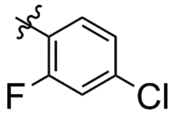	H	16.26
**5**	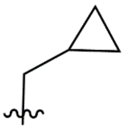	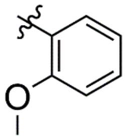	H	4.12
**6**	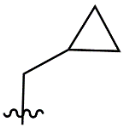	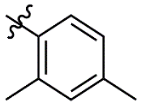	H	4.21
**7**	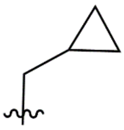	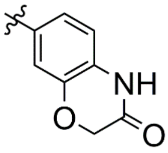	H	5.11

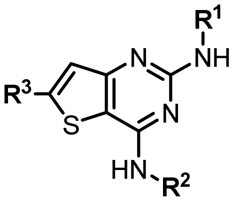				
**No**	**R¹**	**R²**	**R³**	**Osteogenesis IC_{50} (μM)**
**8**	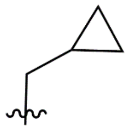	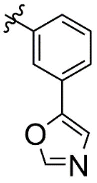	H	8.18
**9**	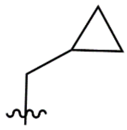	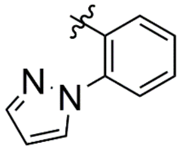	H	20.66
**10**	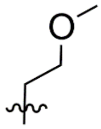	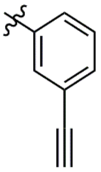	H	8.62
**11**	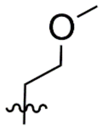	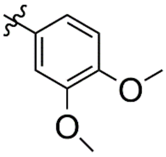	H	16.91
**12**	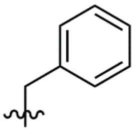	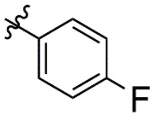	H	9.97
**13**	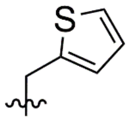	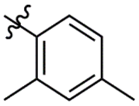	H	11.69
**14**	CH_3	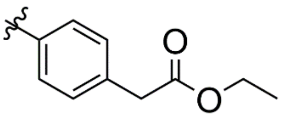	H	14.57

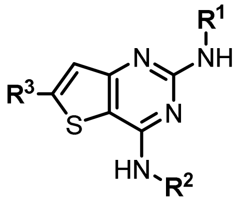

No	R^1	R^2	R^3	Osteogenesis IC_{50} (µM)
**15**	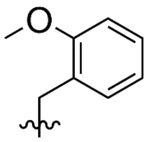	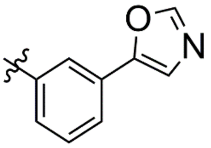	H	5.07
**16**	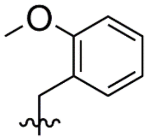	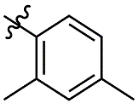	H	6.86
**17**	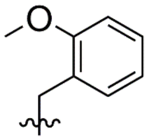	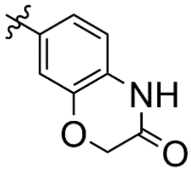	H	11.29
**18**	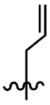	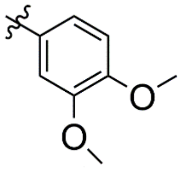	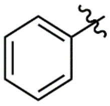	1.75
**19**	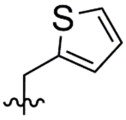	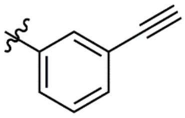	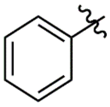	6.70
**20**	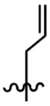	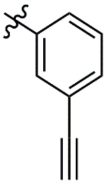	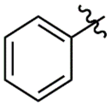	10.68
**21**	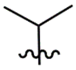	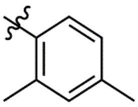	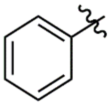	13.42

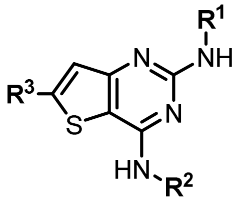

No	R^1	R^2	R^3	Osteogenesis IC_{50} (µM)
**22**	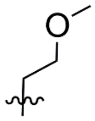	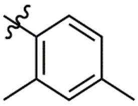	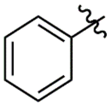	17.76
**23**	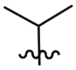	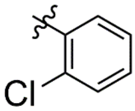	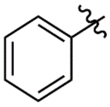	20.76

a C3H10T1/2 cells were incubated with 1.5 µM Purmorphamine and the compounds or DMSO as a control for 96 h. The activity of alkaline phosphatase, an enzyme that is expressed upon induction of osteogenesis, was assessed by means of a luminescence readout. Data were measured in triplicates. Viability was measured in the same setting using a CellTiter Glo Kit (Promega). IC_{50} values > 10 µM were obtained when the inhibition achieved up to a concentration of 10 µM was not sufficient to calculate an IC_{50} value.

Supporting Table S2 (related to Figure 3) Results of the kinase panel with 10 µM Pipinib. Data can be found in the included Excel file.f

Supporting Table S3. Kinase panel hits. Kinases for which inhibition or displacement by 10 µM Pipinib was clearly above 50 % were considered hits. Assays were performed at SelectScreen™, Thermo Fisher Scientific.

**Kinase**	**inhibition (i)/ displacement (d) (%)**
Phosphatidylinositol 4-kinase IIIβ (PI4KB)	76 ± 2 (i)
Cyclin G associated kinase (GAK)	73 ± 1 (d)
Ttk protein kinase (TTK)	69 ± 3 (i)
Mitogen-activated protein kinase 8 (MAPK8 (JNK1))	65 ± 2 (d)
Phosphatidylinositol 4-phosphate 5-kinasae type 1γ (PIP5K1C)	56 ± 0 (i)
Myosin light chain kinase family member 4 (MYLK4)	53 ± 1 (d)
Phosphatidylinositol-4-phosphate 3-kinase catalytic subunit type 2γ (PIK3C2G)	53 ± 3 (i)
G protein-coupled receptor kinase 7 (GRK7)	53 ± 3 (i)

Supporting Table S4 (related to Figure 3). Further kinase activity measurements to assess inhibition. Kinase assays were performed at 10 µM Pipinib at SelectScreen (MAPK8) and Reaction Biology (MYLK4).

Kinase	inhibition (%)
Mitogen-activated protein kinase 8 (MAPK8 (JNK1))	8 ± 4
Myosin light chain kinase family member 4 (MYLK4)	41.5 ± 0.1

2. Supporting Figures

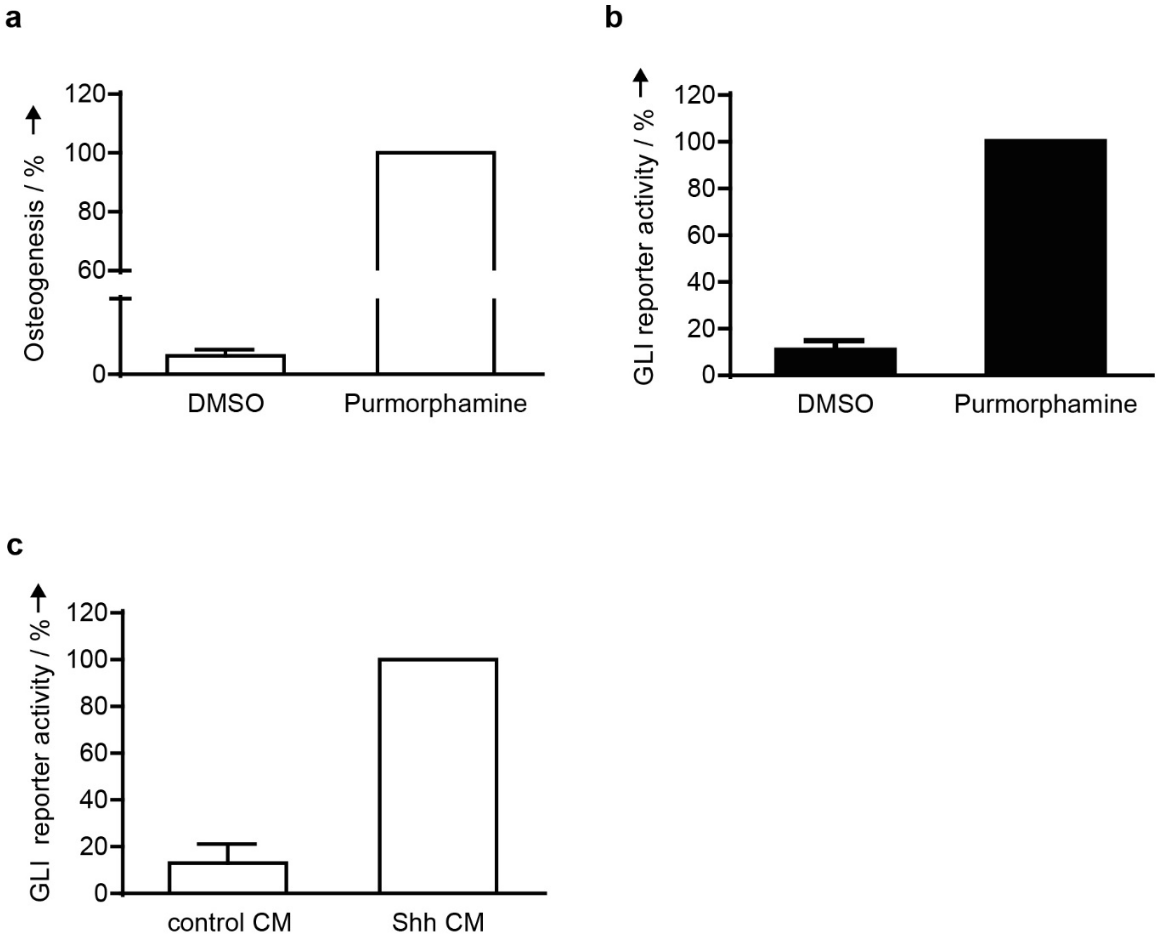

Supporting Figure S1 (related to Figure 1). a. Osteoblast differentiation assay using C3H10T1/2 cells. C3H10T1/2 cells were treated with 1.5 µM Purmorphamine or DMSO as a control for 96 h. Activity of alkaline phosphatase was determined as a measure of differentiation. DMSO-Purmorphamine was set to 100%. Data are mean values ± SD of three biological replicates. **b.** GLI reporter gene assay using Shh-LIGHT2 cells. Shh-LIGHT2 cells were treated with 2 µM Purmorphamine or DMSO as a control for 48 h. The GLI-responsive firefly reporter signal was divided by the control signal of the *Renilla* reporter and DMSO-Purmorphamine was set to 100%. Data are mean values ± SD of three biological replicates. **c**. GLI reporter gene assay using Shh coditioned medium (Shh CM) in NIH/3T3 cells. NIH/3T3 cells were treated Shh CM or conrol conditioned medium 48 h. The GLI-responsive firefly reporter signal was divided by the control signal of the renilla reporter and DMSO-Shh CM was set to 100%. Data are mean values ± SD of three biological replicates.

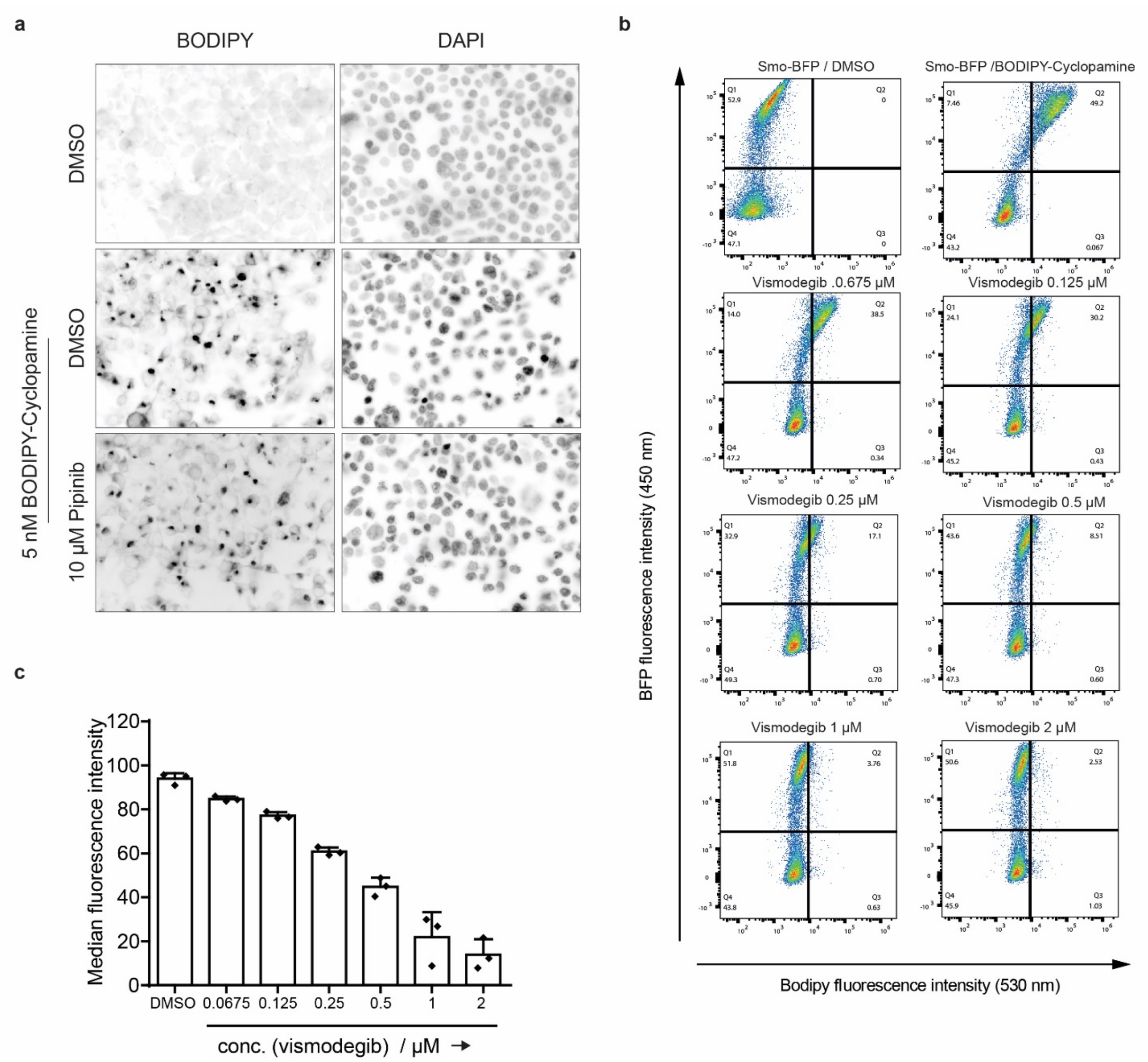

Supporting Figure S2 (related to Figure 2). Displacement of BODIPY-Cyclopamine from SMO. a. HEK293T cells were transfected with a SMO expression construct. 48 h later cells were treated with the compound and BODIPY-Cyclopamine or DMSO as a control for 5 h prior to fixation and staining with DAPI to visualize the nuclei. Complementary images belonging to Figure 2a. **b** and **c.** Quantitative Smo binding assay. HEK293T cells were transfected with a SMO-BFP expression construct. 48 h later, cells were treated with 5 nM BODIPY-Cyclopamine and Vismodegib or DMSO as a control for 4 h. Cells were then harvested and subjected to flow cytometry to quantify the intensity of BODIPY-Cyclopamine. **b**. Representative dot plots. **c**. Quantification. Only BFP-positive, i.e. SMO expressing, cells were considered for the calculation. Data are mean values of three biological replicates ± SD.

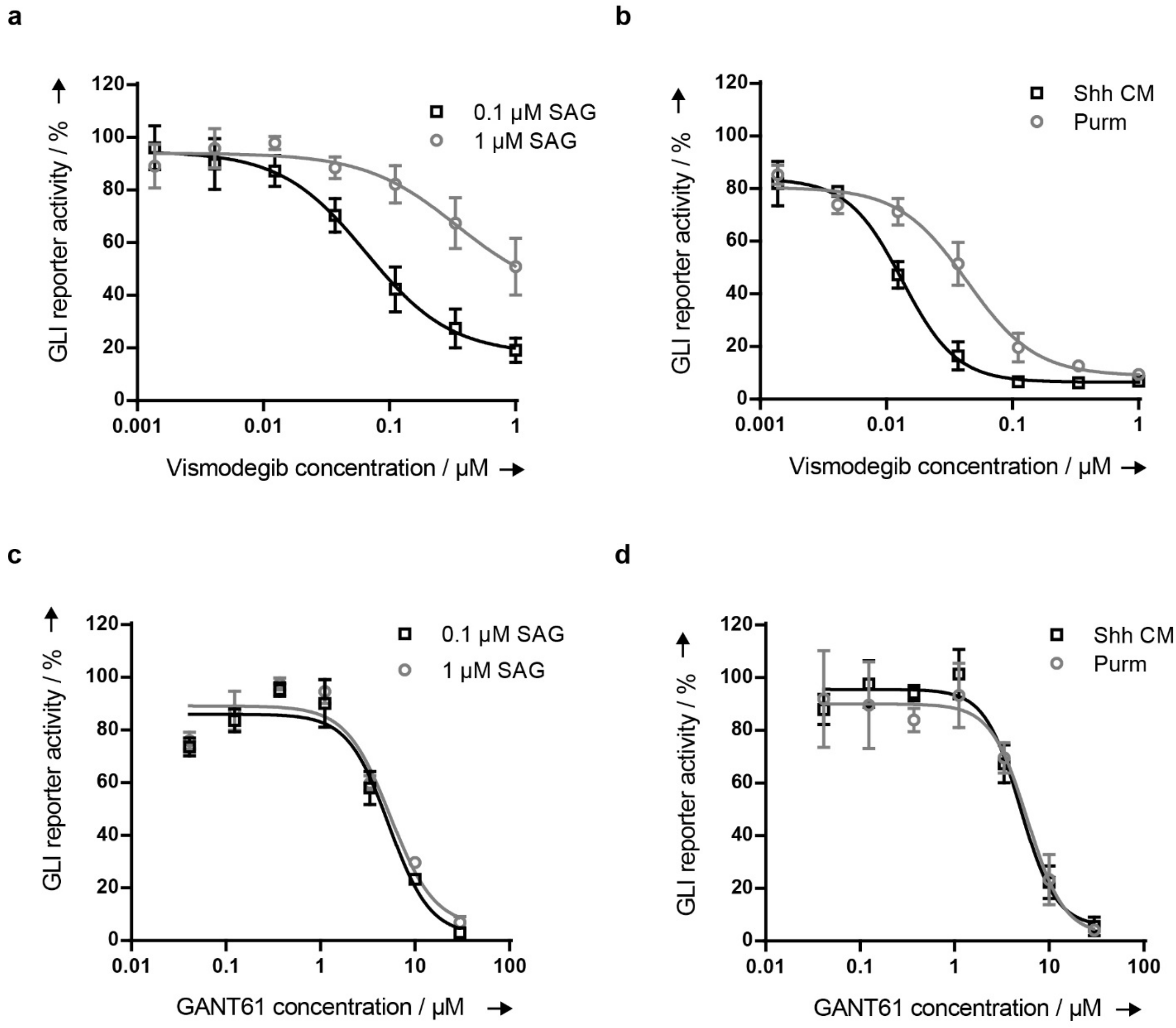

Supporting Figure S3 (related to Figure 2). GLI reporter gene assay using Shh-LIGHT2 cells. Shh-LIGHT2 cells were treated with different concentration of SAG (**a** and **c**) or 2 µM Purmorphamine or Shh conditioned medium (Shh CM) (**b** and **d**) and Vismodegib (**a** and **b**) or GANT61 (**c** and **d**) or DMSO as a control for 48 h. The GLI-responsive firefly reporter signal was divided by the control signal of the *Renilla* reporter. Data are mean values ± SD of three biological replicates.

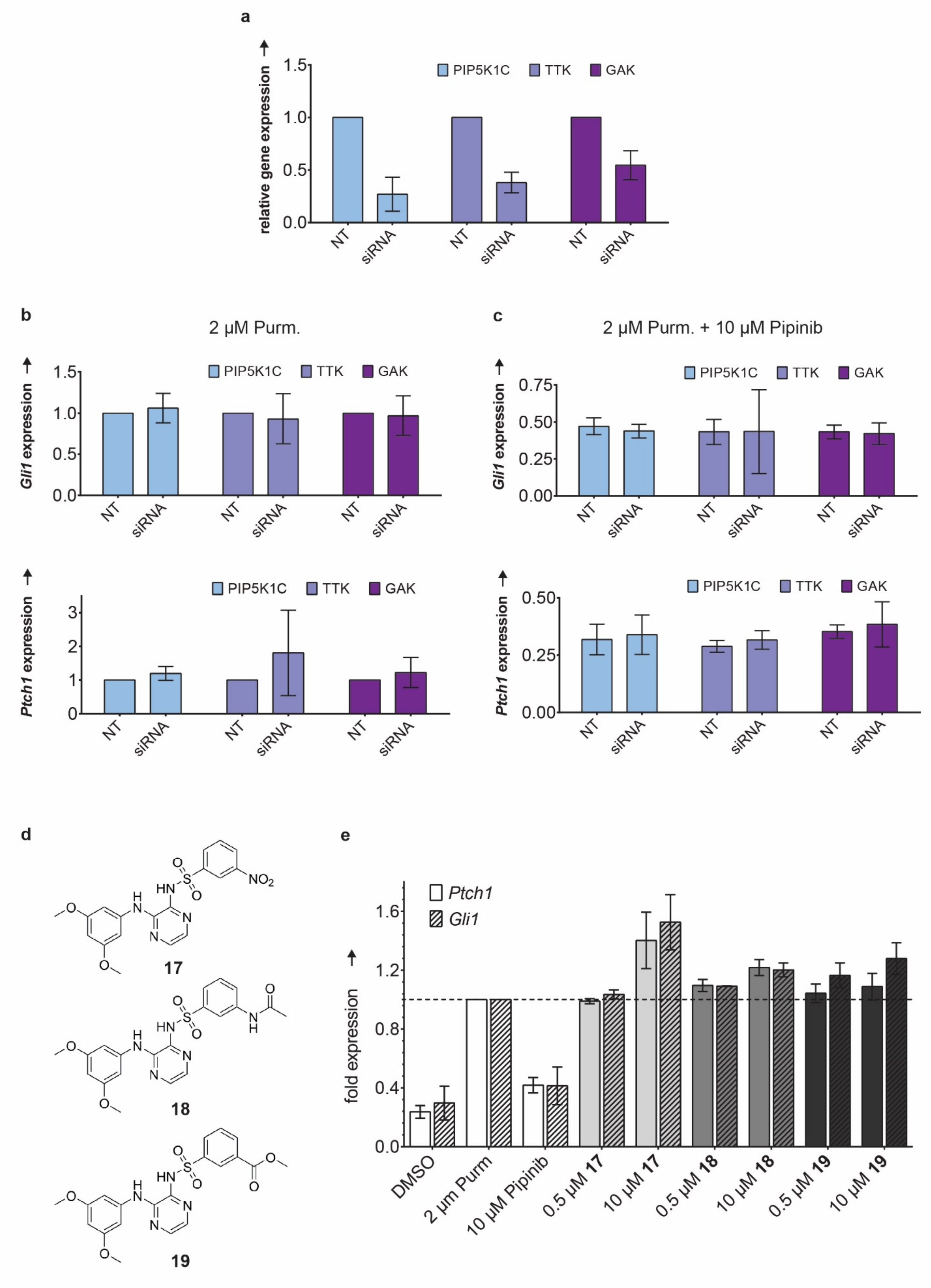
a
relative gene expression
1.5
1.0
0.5
0.0
PIP5K1C
TTK
GAK
NT
siRNA
b
2 μM Purm.
Gli1 expression
PIP5K1C
TTK
GAK
Ptch1 expression
c
2 μM Purm. + 10 μM Pipinib
0.75
0.50
0.25
0.00
d
NO2
HN
H
N
O
S
17
18
19
e
Ptch1
Gli1
fold expression
1.6
1.2
0.8
0.4
0.0
DMSO
2 μm Purm
10 μM Pipinib
0.5 μM 17
10 μM 17
0.5 μM 18
10 μM 18
0.5 μM 19
10 μM 19

Supporting Figure S4 (related to Figure 3). Evaluation of potential kinases with regard to Hh signaling. a. Knockdown efficiency was determined via RT-qPCR. NIH/3T3 cells were incubated with 50 nM PIP5K1C, TTK or GAK siRNA (siRNA) or non-targeting siRNA (NT) as a control for 48 h prior to DMSO treatment for another 48 h. Afterwards, cells were subjected to RNA isolation, cDNA preparation and quantitative PCR (RT-qPCR) using primers specific for *Pip5k1c, Ttk, Gak* and *Gapdh* as a reference gene. NT samples were set to 1. Data are mean values ± SD of three independent experiments (n=3). **b** and **c.** siRNA-mediated knockdown of PIP5K1C, TTK and GAK. NIH/3T3 cells were incubated with 50 nM PIP5K1C, TTK or GAK siRNA (siRNA) or non-targeting siRNA (NT) as a control for 48 h prior to treatment with 2 µM Purmorphamine (**b**) or 2 µM Purmorphamine and 10 µM Pipinib (**c**) for another 48 h. Afterwards, cells were subjected to RNA isolation, cDNA preparation and quantitative PCR (RT-qPCR) using primers specific for *Ptch1, Gli1* and *Gapdh* as a reference gene. Values for Purmorphamine (Purm.)-DMSO-treated cells that were incubated with non-targeting siRNA were set to 1. Data are mean values ± SD of three independent experiments (n=3). **d.** Structures of PI3KC2G inhibitors **17**, **18** and **19**. **e.** Influence of PIK3C2G inhibitors on Hh target gene expression. NIH/3T3 cells were incubated with 2 µM Purmorphamine and the compounds or DMSO as a control for 48 h prior to RNA isolation, cDNA preparation and quantitative real-time PCR using primers specific for *Ptch1* and *Gli1* and *Gapdh* as a reference gene. Values for Purmorphamine-DMSO-treated cells were set to 1. Data are mean values ± SD of three independent experiments (n=3).

a

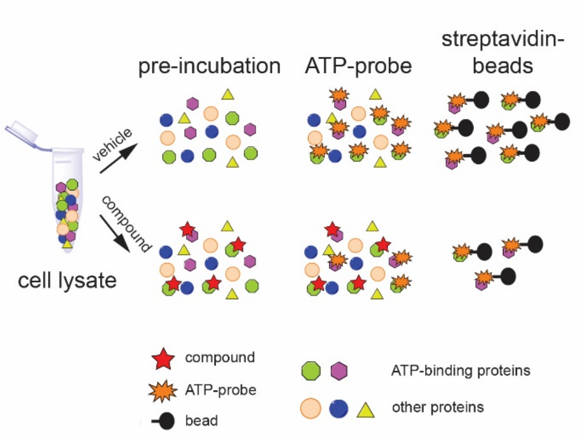

b

n1	n2	n3	count identified
Acaa2			1
	Acad10		1
		Blmh	1
	Cav1		1
Cdc42			1
	Commd2		1
	Dctn1		1
		Dsp	1
	Gm5039		1
	Gm8991		1
	H3f3a		1
		Hnrnpm	1
	Pdk1		1
**Pi4kb**	**Pi4kb**	**Pi4kb**	**3**
	Psmd7		1
Rfk			1
Rock2			1
Rpl23a			1
	Ube2v1		1

c

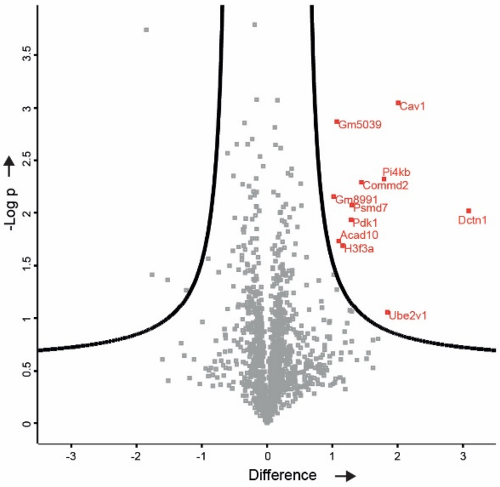

d

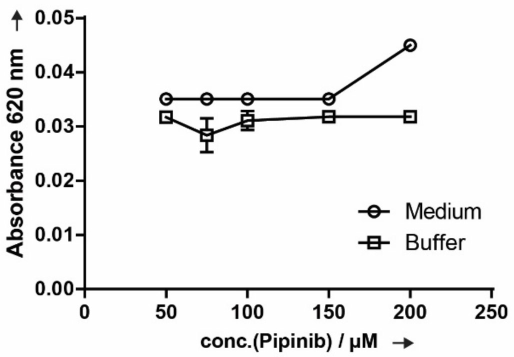

e

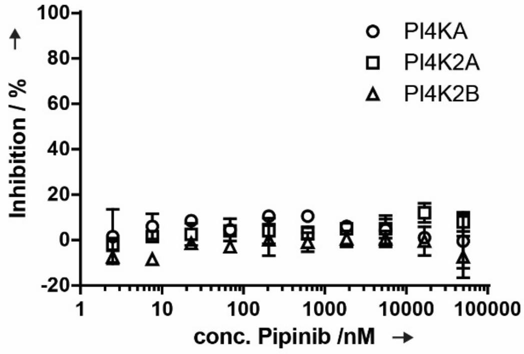

Supporting Figure S5 (related to Figure 3). ActiveX affinity chromatography (ATP pulldown). a. Assay principle. NIH/3T3 lysate was preincubated either with compound or DMSO as a vehicle control. Next, the lysate was supplemented with a desthiobiotin-ATP probe and afterwards exposed to streptavidin beads. Streptavidin binding to desthiobiotin-ATP allows enrichment of ATP-binding proteins. Proteins that bind to the compound and thus are not labelled (or to a lesser extent) by the probe would be enriched to a lesser extent than from the control lysate. Detection of proteins can either be achieved via immunoblotting or mass spectrometry. **b.** Results of the mass spectrometry analysis after ATP pulldown and protein digestion. NIH/3T3 lysate was incubated with 200 µM Pipinib or DMSO as a control for 60 min and then incubated with 5 µM desthiobiotin-ATP for 10 min before addition of streptavidin beads to enrich ATP-binding proteins. Bound proteins were digested and eluted peptides were subjected to MS/MS quantification. The table shows significant hits for all three independent biological replicates of the experiment (n=3, each performed in technical triplicates). Statistical significance was assessed by means of a t-test for each technical triplicate. **c.** Volcano plot for experiment n2 from **b**. The results of a t-test are visualized. The t-test is able to determine proteins which differ statistically significant in label-free quantification (LFQ) values in the DMSO group compared to the compound treated group. The '- log p' value resulting from the t-test is plotted against the difference of the logarithmized (log 2) LFQ values (DMSO group - compound treated group). Proteins, which had significantly lower intensities in the compound-treated group compared to the DMSO group, are marked in red. The black lines indicate significant borders (s0 = 0, FDR = 0.05). **d.** Turbidimetric assessment of Pipinib aggregation in aqueous buffer or medium. **e**. Influence of Pipinib on the activity of PI4KA, PI4K2A and PI4K2B. Enzymatic activity was determined in an Adapta activity assay. Data are mean values (N=2) ± S.D. and representative of two independent experiments.

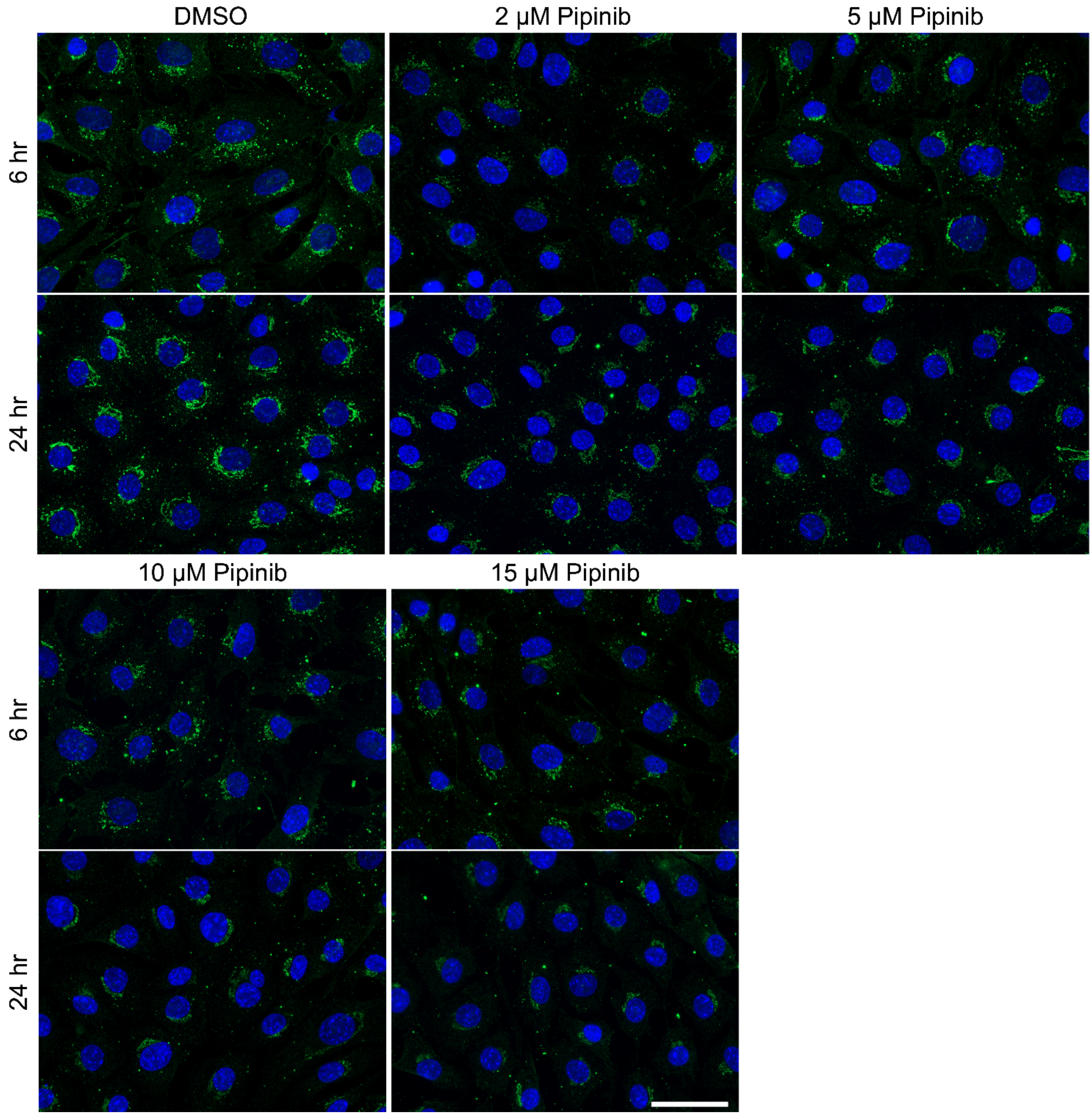

Supporting Figure S6 (related to Figure 4). Qualitative assessment of intracellular PI4P levels. NIH/3T3 cells were incubated with Pipinib or DMSO as a control for 6 or 24 h. Cells were fixed and stained with an antibody against PI4P (green) and DAPI (nucleus, blue). Full-size images belonging to Figure 4a (three independent experiments, n=3, scale bar=50 µm).

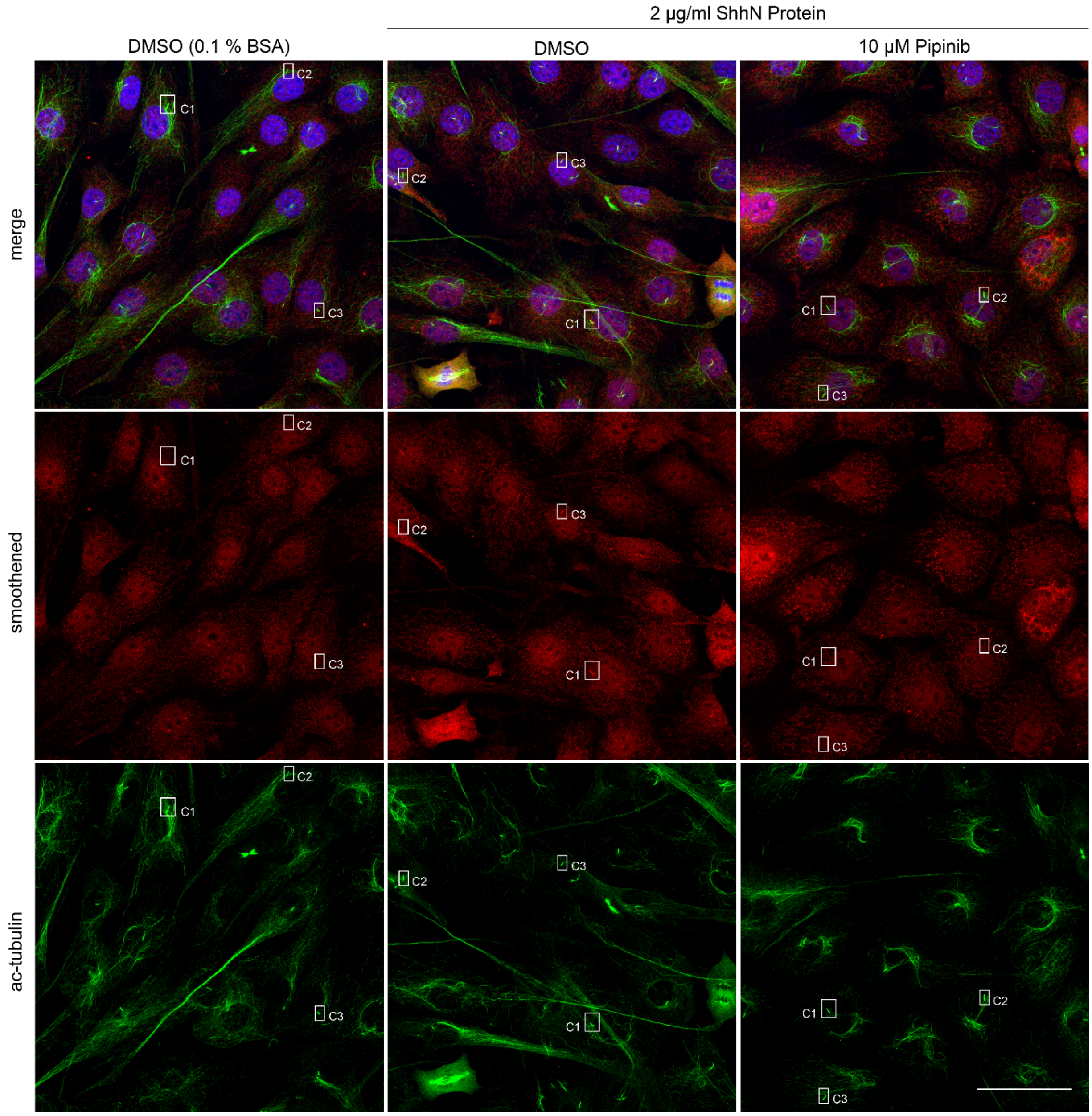

Supporting Figure S7 (related to Figure 5). SMO trafficking. NIH/3T3 cells were serum starved for 24 h to induce ciliation prior to incubation with 10 µM Pipinib or DMSO as a control for 24 h, followed by co-incubation with 2 µg/ml ShhN for another 24 h. Cilia were then stained with an antibody against acetylated tubulin (ac-tubulin, green), SMO was stained with an anti-SMO antibody (red) and the nucleus was marked with DAPI (blue). Full-size images belonging to Figure 5a, representative of three independent experiments (n=3, scale bar: 50 µm).

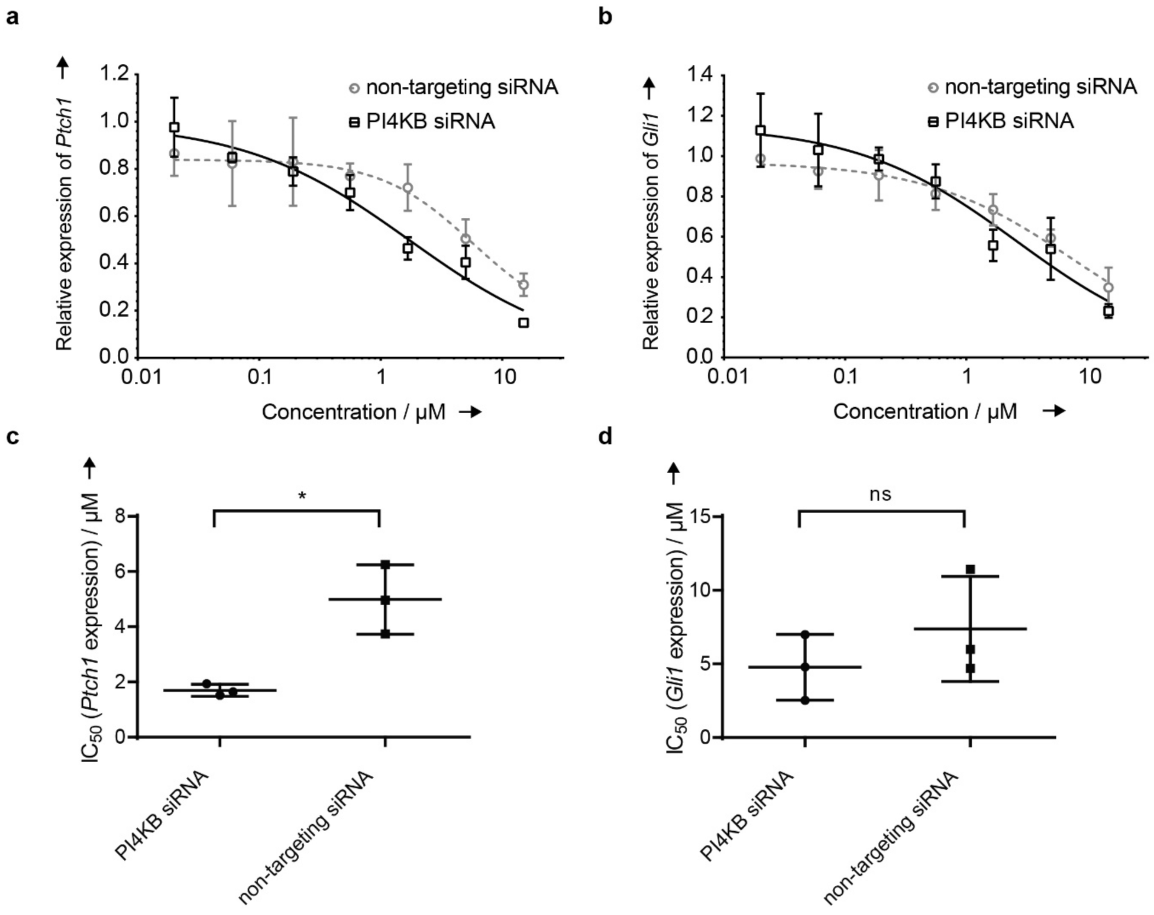

Supporting Figure S8 (related to Figure 6). Dose dependency of Pipinib-mediated inhibition of target gene expression after siRNA knockdown of PI4KB. NIH/3T3 cells were incubated with 30 nM PI4KB siRNA or non-targeting siRNA as a control for 48 h prior to treatment with Pipinib for another 48 h. Cells were subjected to RNA isolation, cDNA preparation and quantitative PCR using primers specific for *Ptch1* and *Gli1* and *Gapdh* as a reference gene. Values for Purmorphamine-DMSO-treated cells were set to 1. Data are mean values ± SD of three independent experiments (n=3). **a.** Expression of *Ptch1.* **b.** Expression of *Gli1*. IC_{50} values for suppression of *Ptch1* (**c**) or *Gli1* (**d**) expression by Pipinib were compared for cells with or without PI4KB depletion (from a and b). Statistical analysis was performed using two-tailed t-test. *: $p<0.05$; ns: not significant.

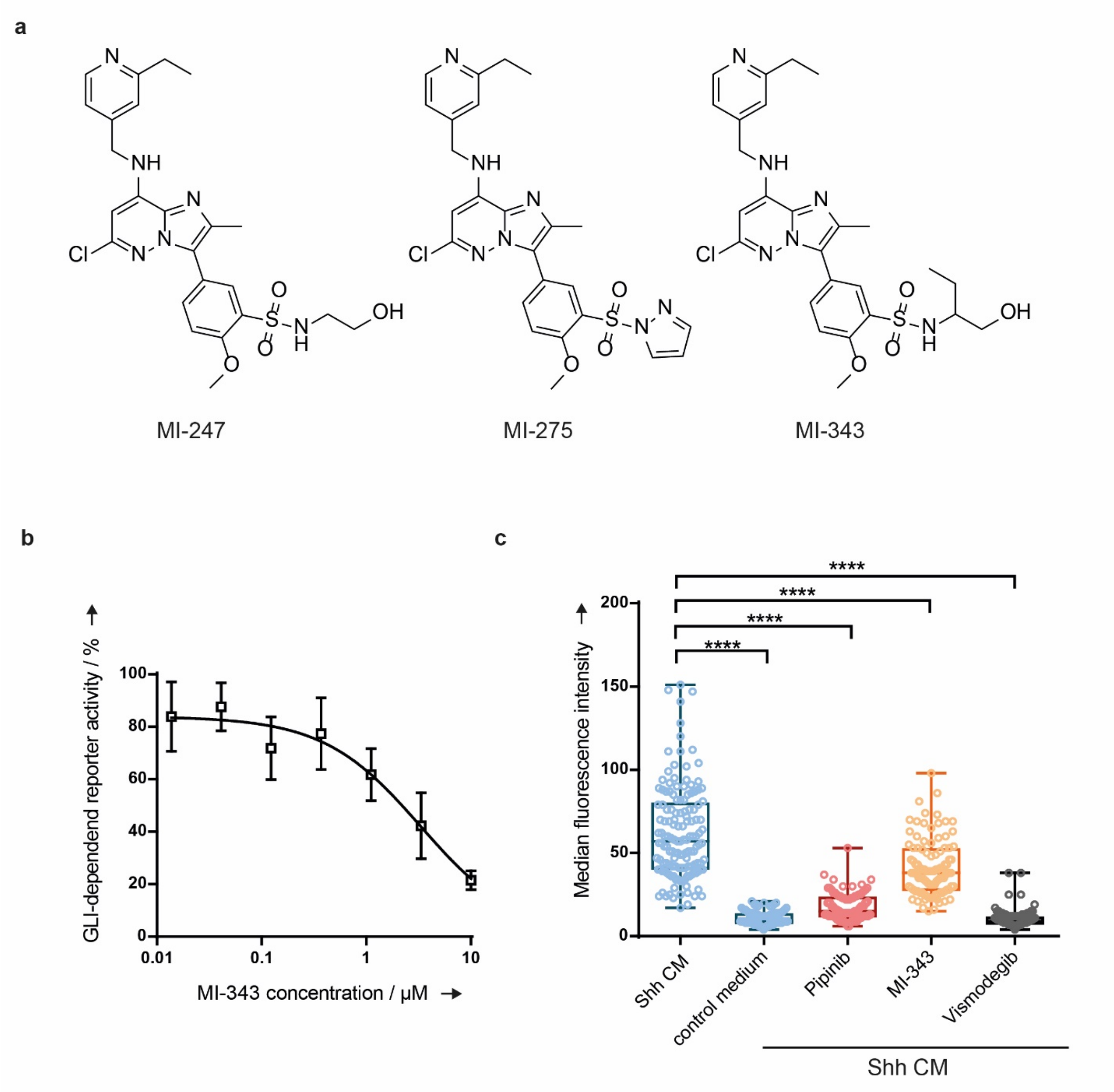

Supporting Figure S9 (related to Figure 5). Influence of unrelated PI4KB inhibitors on Hh signaling. a. Structures of PI4KB inhibitors MI-247, MI-275 and MI-343. **b.** GLI reporter gene assay using Shh-LIGHT2 cells. Shh-LIGHT2 cells were treated with 2 µM Purmorphamine, and MI-343 or DMSO as a control for 48 h. The GLI-responsive firefly reporter signal for Purmorphamine/DMSO-treated cells was set to 100%. Data are mean values ± SD of three biological replicates. IC_{50} = 2.3 ± 1.3 µM. **c.** Quantification of ciliary localization of SMO. SMO localization was detected upon ciliation and incubation with Shh condititioned medium (ShhN CM) and the compounds (10 µM Pipinib, 10 µM MI-343 or 1 µM Vismodegib) for 48 h. At least 100 cilia of a representative experiment were analyzed for the intensity of the anti-SMO antibody. Each data point represents the intensity value of a single cilium. Statistical significance was assessed using an unpaired t-test; ****: p<0.0001.

3. Supporting Movies

Supporting movie 1: NIH/3T3 transfected with GT-CFP and treated with DMSO.

Supporting movie 2: NIH/3T3 transfected with YFP-PH (Fapp1) and treated with DMSO.

Supporting movie 3: NIH/3T3 transfected with GT-CFP and treated with 30 µM Pipinib

Supporting movie 4: NIH/3T3 transfected with YFP-PH (Fapp1) and treated with 30 µM Pipinib.

4. Experimental procedures – Biology

Reagents

Purmorphamine was purchased from Cayman Chemical (#10009634). Vismodegib was obtained from Selleckchem (#S1082). All other reagents and the respective source of supply are mentioned within the single protocols.

Cell lines

The murine fibroblast cell line NIH/3T3 (DSMZ, ACC-59) was cultured in Dulbecco's Modified Eagle's medium (DMEM, high glucose, PAN, #P04-03550) supplemented with 10% fetal calf serum (Fisher Scientific, #10136253, heat-inactivated), 2 mM L-glutamine (PAN, #P04-80100) and 1 mM sodium pyruvate (PAN, #P04-43100). NIH/3T3 cells, stably transfected with a Gli-responsive firefly luciferase reporter plasmid [1] and a pRL-TK constitutive Renilla-luciferase expression vector (Promega) (Shh-LIGHT2 cells [2]), were cultured in the same culturing medium as the parental cell line, additionally supplemented with 400 µg/ml Geneticin (Sigma Aldrich, #A1720) and 150 µg/ml Zeocin (InvivoGen, #ant-zn-1) as selecting agents. The murine C3H/10T1/2 cell line (ATCC, CCL-226) was cultured in Dulbecco's Modified Eagle's medium (DMEM, high glucose, PAN, #P04-03550) supplemented with 10% fetal calf serum (Fisher Scientific, #10136253, heat-inactivated), 6 mM L-glutamine (PAN, #P04-80100), 1 mM sodium pyruvate (PAN, #P04-43100) as well as penicillin/streptomycin (PAN, #P06-07100). The human embryonic kidney cell line HEK293T (ATCC, CRL-11268) was cultured in Dulbecco's Modified Eagle's medium (DMEM, high glucose, PAN, #P04-03550) supplemented with 10% fetal bovine serum (FBS, Thermo Fisher Scientific, #10270106), 1 mM sodium pyruvate (PAN, #P04-43100) and MEM non-essential amino acids (PAN, #P08-32100). 3T3-GBS cell lines were generated as described (Phua et al. Cell 2017 http://dx.doi.org/10.1016/j.cell.2016.12.032). 3T3-GBS were cultured in DMEM containing 10% FBS and 2 mM GlutaMAX (Gibco). NIH/3T3 cells were

cultured for live-cell imaging in DMEM (Corning, 10-013-CV) supplemented with 10% fetal bovine serum (Sigma, F6178). All cell lines were maintained at 37°C and 5% CO_2 in humidified atmosphere. All cell lines were regularly tested for mycoplasma contaminations and were found to be free of contamination at all times. None of the used cell lines has been listed in the database of commonly misidentified cell lines maintained by the ICLAC.

Preparation of ShhN-conditioned medium

The ShhN-conditioned medium was obtained by transient transfection of HEK293T cells (ATCC, CRL-11268). Briefly, $2x10^6$ cells were seeded into a T-75 cell culture flask and grown overnight. Afterwards, cells were transfected with a ShhN-encoding plasmid (pcDNA3.1 ShhN was a gift from Philip Beachy; Addgene plasmid # 37680; http://n2t.net/addgene:37680; RRID:Addgene_37680), or with an empty vector (Invitrogen) as a control, using FuGENE HD transfection reagent (Promega). After incubation for 24 h the medium was exchanged by serum-reduced medium (DMEM, supplemented with 0.5% FCS and 1 mM sodium pyruvate. Another 24 h later, the medium was harvested by 20 min centrifugation at 2000 xg, sterile filtered using a 0.2 µm syringe filter (Sarstedt) and finally stored at 4°C. Cells were then supplied with fresh serum-reduced medium and were again incubated for 24 h. Afterwards, the ShhN-conditioned medium was harvested for a second time, as described above. For assays, both fractions were combined and diluted with fresh serum-reduced medium in a ratio of 3:1.

Osteogenesis Assay

The osteogenesis assay was performed as published before.[10] Eight hundred C3H/10T1/2 cells were seeded per well in white 384-well plates (Greiner, # 781080). On the next day, cells were treated with 1.5 µM Purmorphamine and different concentrations of the compound or DMSO as a control. After 96 h, cells were lysed (lysis buffer: 100 mM Tris pH 9.5, 250 mM NaCl, 25 mM

$MgCl_2$, 1% Triton X-100, sterile filtered) and the luminogenic alkaline phosphatase (AP) substrate CDP-Star (Roche, #11685627001) was added to the wells to detect AP activity. One hour after addition of CDP-Star, luminescence was measured on an Infinite M200 plate reader (Tecan, Austria). DMSO was set to 100%. To obtain IC_{50} values, nonlinear regression was performed using a four-parameter fit (GraphPad Prism 6, GraphPad Software, USA).

Reporter gene assay

The reporter gene assay was performed as published before [10] $2.5x10^4$ Shh-LIGHT2 cells were seeded per well in 96-well plates (Sarstedt, #83.3924). After incubation overnight, cells were treated with 2 µM Purmorphamine, 0.1 µM or 1 µM SAG (Calbiochem, #566660) in serum-reduced medium or with ShhN-conditioned medium to activate Hh signaling and the compound at different concentrations or DMSO as a control for 48 h. Luciferase expression and activity was detected by means of the Dual-Luciferase Reporter Assay System (Promega, #E1960) using the Infinite M200 plate reader (Tecan, Austria). The firefly luciferase signal was normalized to the *Renilla* luciferase signal and DMSO or DMSO-Purmorphamine was set to 100%. To obtain IC_{50} values, nonlinear regression was performed using a four-parameter fit (GraphPad Prism 6, GraphPad Software, USA).

Reverse Transcription Quantitative PCR (RT-qPCR)

The RT-qPCR was performed as published before [3]. NIH/3T3 cells were seeded in 24-well plates (Sarstedt, #83.3922, $2x10^4$ cells/well). After incubation overnight, cells were treated with 2 µM Purmorphamine together with the compound or DMSO as a control, diluted in assay medium (DMEM, high glucose (PAN, #P04-03550) containing 0.5% FBS (Thermo Fisher

Scientific, #10270106)) for 48 h. RNA was isolated using the RNeasy Kit (Qiagen, #74104), cDNA was synthesized using the QuantiTect Reverse Transcription Kit (Qiagen, # 205313). The relative mRNA amount of the Hedgehog target genes *Ptch1* and *Gli1* and the reference gene *Gapdh* was assessed using the QuantiFast SYBR Green PCR Kit (Qiagen, # 204054) using the following primers: *Gapdh*: 5'-CAGTGCCAGCCTCGTC-3' and 5'-CAATCTCCACTTTG-CCACTG-3'; *Ptch1* [4]*:* 5'-CTCTGGAGCAGATTTCCAAGG-3' and 5'-TGCCGCAGTTCTT-TTGAATG 3'. *Gli1* [5]*:* 5'-CACCGTGGGAGTAAACAGGCCTTCC-3'. The SYBR Green signal was detected with an iQ™5 Real-Time PCR Detection System (Bio-Rad, Germany). Relative expression levels were calculated using the $\Delta\Delta C_t$ method with *Gapdh* as a reference gene [6]. Expression levels of *Ptch1* and *Gli1* were related to the levels of the respective gene in Purmorphamine-treated cells (set to 1).

Smoothened trafficking assay (Ciliary localization of SMO)

The Smoothened trafficking assay was performed as published before [3], with some modifications. To detect trafficking of Smoothened, cells were seeded on cover slips (Thermo Scientific, 12 mm, #10062491) placed in 24-well plates (Sarstedt, #83.3922). After incubation overnight, the medium was changed to assay medium to starve the cells and induce ciliation. After 24 h, compounds were added. Alternatively, medium was exchanged to ShhN-conditioned medium and compounds were added, for 48 h. After another 24 h, compounds and 2 µg/ml ShhN were added for additional 24 h. Cells were then fixed in 4% paraformaldehyde in ice-cold PBS for 10 min, washed three times with ice-cold PBS and incubated in blocking solution (1% heat-inactivated horse serum, 0.1% Triton X-100 in PBS) for 30 min at room temperature. Cells were incubated with the primary antibodies diluted in blocking solution overnight at 4°C. Anti-Smoothened antibody (abcam, #ab38686 or Santa Cruz Biotechnology, #sc-166685), and anti-tubulin acetylated antibody (Sigma, #T6793 or Cell Signaling Technology, #5335S) were used to visualize the Smoothened receptor and the primary cilium, respectively. Cells were then

washed three times with 0.1% Triton X-100 in PBS and incubated with secondary antibodies and DAPI in blocking solution for 45 min at room temperature. A donkey anti-mouse Alexa488-conjugated antibody (Invitrogen, A-21202) and a goat anti-rabbit Alexa594-conjugated antibody (Invitrogen, A-11012) were used. Afterwards, cells were washed two times in 0.1% Triton X100 in PBS, once in PBS and mounted on glass slides. Imaging was performed with a Leica SP8 or SP5 confocal microscope, equipped with 63x 1.4 NA objective (Leica Microsystems CMS GmbH, Mannheim, Germany). Images were acquired as Z-sections and converted to maximal intensity projections using the software ImageJ [7] for illustrative purposes. The staining protocol was adopted (with modifications) from Rohatgi et al. [8]. Quantification was carried out employing ImageJ [7]. For this, acetylated tubulin-positive cilia were marked and the intensity of the anti-Smoothened antibody was measured within this area. Statistical significance was assessed using an unpaired t-test (GraphPad Prism 6, GraphPad Software, USA) with a confidence level of 95% (*=$p<0.05$, **=$p<0.005$, ***=$p<0.0005$, ****=$p<0.0001$). Data of three independent experiments was used to generate the data presented.

Smoothened binding assay

Microscopy analysis of BODIPY-Cyclopamine labeling of cells was performed as described by Sinha et al. [9]. Briefly, HEK293T cells were seeded on cover slips (Thermo Scientific, 12 mm, #10062491) at $1.5x10^4$ cells per well in 24-well plates (Sarstedt, #83.3922). After incubation overnight, cells were transfected with a SMO-expression construct (pGEN-mSMO, was a gift from Philip Beachy, Addgene plasmid # 37673) [2] using Fugene HD (Promega, # E2311). Two days after transfection, cells were fixed in phosphate-buffered saline (PBS) containing 3% paraformaldehyde for 10 min, treated with PBS containing 10 mM glycine and 0.2% sodium azide for 5 min and washed with PBS. Fixed cells were then treated with the compound or DMSO in DMEM containing 0.5% FBS (assay medium) and 5 nM BODIPY-Cyclopamine

(Carbosynth Limited, FB18988) for four hours at room temperature. Afterwards, cover slips were washed with PBS and incubated with 1 µg/ml 4',6 diamidino-2-phenylindole (DAPI, Sigma Aldrich, Roche, #10236276001) in PBS for 10 min at room temperature. Cover slips were then washed again and mounted onto glass slides. Samples were examined by means of fluorescence microscopy using the Zeiss Observer Z1 (Carl Zeiss, Germany) and a Plan-Apochromat 63x/1.40 Oil DIC M27 objective. For the quantitative SMO binding assay, flow cytometry analysis of BODIPY-Cyclopamine labeled cells was adopted for HEK293T cells from Chen et al. [10]. Briefly, HEK293T cells were seeded in 6-well plates (Sarstedt, #83.3920) at $2.5x10^5$ cells per well. After incubation overnight, cells were transfected with a SMO-BFP-expression construct [3]. Two days later, cells were treated with the compound or DMSO in assay medium and 5 nM BODIPY-Cyclopamine for five hours at 37°C. Cells were then detached with trypsin/EDTA (PAN, P10-023100), diluted in assay medium and centrifuged at 129 x g for 5 min at room temperature. Cells were resuspended in ice-cold PBS, collected at 129 x g for 5 min at 4°C and finally resuspended in ice-cold PBS prior to flow cytometry analysis employing the BD LSR II Flow Cytometer (BD Biosciences, USA) (laser line: 488 nm, emission filter: 530/30 for BODIPY and laser line: 405 nm, emission filter: 450/50 for BFP). Data were analyzed with the FlowJo software, version 7.6.5 (Tree Star Inc., USA).

End-point evaluation of PI4P levels

For detection via immunofluorescence staining, NIH/3T3 cells were seeded into 24-well plates (Sarstedt, #83.3922) containing Ø 12 mm coverslips at a concentration of $2x10^4$/well. The next day, compound or DMSO as a control was added to the cells. After an incubation of 2, 6, 24 or 48 h, cells were fixed in 1.5% Formaldehyde in PBS for 15 min, quenched with 50 mM NH_4Cl in PBS for 10 min and washed with PBS for 5 min. After blocking with 3% gelatine, 0.2% Saponin in PBS for 30 min at RT, mouse anti-PI4P antibody (echelon, # Z-P004) was added in PI4P blocking buffer at a 1:200 dilution. After incubation at 4°C overnight, the samples were washed

with PBS for 5 min and incubated with the secondary antibody (donkey anti-mouse-alexa-488, Thermo Fisher Scientific, # A-21202) and 1 µg/ml DAPI in blocking buffer for 45 min at RT. Finally, the cells were washed two times with PBS for 5 min before they were mounted on glass object slides for microscopic examination using the Zeiss Observer Z1 (Carl Zeiss, Germany) and a Plan-Apochromat 63x/1.40 Oil DIC M27 objective. For quantitative detection of PI4P via flow cytometry, NIH/3T3 cells were seeded in a 6-well plate (Sarstedt, #83.3920) at $1x10^5$ cells/well. The next day, cells were treated with the compounds or DMSO (solvent control) for 48 h. Subsequently, cells were washed with PBS, trypsinized and collected in growth medium. The cells were centrifuged at 1000 g for 5 min and resuspended in 200 µL PBS. To fix the cells, 800 µL of a PBS/formaldehyde solution (final concentration of formaldehyde: 3%) were added and the suspension was incubated at room temperature for 10 min. Afterwards, the fixation solution was removed via centrifugation (5 min at 1000 g), the pellet was resuspended in 1 mL 20 µM digitonin in HBS buffer (21 mM HEPES, 1.8 mM Na_2HPO_4, 137 mM NaCl, 4.8 mM KCl in mH_2O) and incubated at RT for 5 min. The digitonin solution was removed via centrifugation, replaced with 300 µL antibody solution (anti-PI4P 1:200 in HBS buffer) and incubated at 37°C for 30 min. The antibody solution was removed via centrifugation (5 min at 1000 g) and the pellet was washed two times with 500 µL HBS before addition of 300 µL secondary antibody solution (anti-mouse-alexa-488 1:500 in HBS buffer) and incubation at 37°C for 20 min. Cells were then centrifuged for 5 min at 1000 g, resuspended in 500 µL HBS and subjected to flow cytometry employing the BD LSR II Flow Cytometer (BD Biosciences, USA) (laser line: 488 nm, emission filter: 530/30 for alexa-488). Data were analysed with the FlowJo software, version 7.6.5 (Tree Star Inc., USA). Statistical significance was assessed using an unpaired t-test (GraphPad Prism 6, GraphPad Software, USA) with a confidence level of 95% (*=$p<0.05$).

Live cell evaluation of PI4P levels

For transient expression, Lyn-CFP plasmid was designed as previously described [11]. GT-CFP (the N-terminal 81 amino acids of human -1,4-galactosyltransferase tagged with ECFP) was purchased from Clontech. YFP-PH(Fapp1) was constructed by fusing PCR-amplifying PH domain of human Fapp1 (1-99) into pEFYP-C1 using EcoRI and SalI sites. mCherry-P4Mx2 was constructed by replacing EGFP region of EGFP-P4Mx2 [12] with mCherry using AgeI and BsrGI. Transient transfection was performed by mixing trypsinized cells with a transfection solution containing Opti-MEM I (Gibco, 31985), a specified set of DNA plasmids and FuGENE HD (Promega, E2311), and plating them on Lab-Tek 8-well chambered cover glasses (ThermoFisher Scientific, 155411PK) coated with poly-D-lysine (Sigma, P6407) at $5x10^4$ cells per well. Cells were incubated in imaging media (DMEM (CORNING, 17-205-CV) and 1x GlutaMAX) supplemented with 10%FBS for 16 to 24 hours before fluorescence imaging started. Live-cell fluorescence imaging was performed on a spinning-disk confocal microscope. CFP, YFP and mCherry excitations were conducted with diode lasers and a semiconductor laser (COHERENT, OBIS 445 nm LX 75 mW, OBIS 514 nm LX 40 mW, OBIS 561 nm LS 50 mW). The lasers were fiber-coupled (OZ optics) to the spinning disk confocal unit (CSU10; Yokogawa) mounted with a triple-band dichroic mirror (Di01-T442/514/647, Semrock). The laser was processed with appropriate filter sets for CFP, YFP and mCherry (Chroma Technology) to capture fluorescence images with a CCD camera (Orca ER, Hamamatsu Photonics) driven by or Micro-Manager 1.4 (Open Imaging). Images were taken using a Neo Fluor 40× objective (Zeiss) mounted on an inverted Axiovert 200 microscope (Zeiss). Images were taken every 1 min for 30 to 60 min. During the experiments, temperature (37°C), CO_2 (5%), and humidity of cell samples were held constant by a stage top incubator and a lens heater (Tokai Hit). Image quantification was performed by ImageJ software. Golgi intensity of YFP-PH(Fapp1) was quantified with a binary mask made from the image of Golgi marker GT-CFP. Cytosolic

intensities of YFP-PH(Fapp1) and mCherry-P4Mx2 were measured by taking arbitrary region inside the cell without covering nucleus and endomembranes.

siRNA knockdown

NIH/3T3 cells were seeded into a 24-well plate (Sarstedt, #83.3922) at $3x10^4$ cells per well. After incubation overnight, the cells were transfected with siRNA using the DharmaFECT1 reagent (Dharmacon, #T-2001-02) according to the manufacturer's instructions. PI4KB siRNA (Dharmacon, #J-056390-07) was used at 30 nM final concentration, GAK (#L-052494-00), TTK (#L-047162-00) and PIP5K1C (#L-042927-01) siRNAs (all from Dharmacon) were applied at 50 nM final concentration. After 48 h, the cells were treated with 2 µM Purmorphamine and the compounds or DMSO as a control for another 48 h. The cells were then lysed and either subjected to immunoblotting or RT-qPCR.

CRISPR/Cas9 mediated PI4KB knockdown

The gateway cloning protocol of the Zhang lab was followed [13] to introduce sgRNA targeting the PI4KB locus into the vector pSpCas9(BB)-2A-GFP (PX458, Addgene #48138). The following oligos were employed to induce double strand breaks of the DNA at two sites (one in PI4KB exon 1 and the other one in PI4KB exon 3), according to the method described by Bauer *et al.* [14] to increase the possibility of a successful gene knockout.

2a	PI4KB Exon 1	CGTCATCACGGAGGGGGTCG
4b	PI4KB Exon 3	GATGGCAATCGGCAAACGGC
5a	PI4KB Exon 1	CCCATTAACCAACTCCAGCG

5b	PI4KB Exon 3	GGCTGGCTTTGACCACCACG
7a	PI4KB Exon 1	AGCCCATCAGGGAATAATGG
8b	PI4KB Exon 3	TGTGGGGCACACGGACCACG

To anneal and phosphorylate forward and reverse sequences of each sgRNA, 100 µM each of the single strand oligos (forward and reverse sequence) were incubated with T4 Polynucleotide kinase at 37 °C for 30 min and then at 95 °C for 5 min before ramping down to 25 °C at steps of 5 °C per minute. To ligate the double stand oligo and the linearized vector (digested with the restriction enzyme BbsI for 30 min at 37 °C according to the manufacturer's instructions), 50 ng of the vector and 1 µL of the oligo reaction mixture were supplemented with 5 µl 2x quick ligation buffer (from the Quick LigationTM kit, New England bioloabs) and filled up to 10 µl with water. After addition of 1 µl Quick Ligase (from the Quick LigationTM kit, New England bioloabs), the reaction mixture was incubated at room temperature for 10 min and transformed into chemically competent One Shot OmniMAXTM 2 T1 cells.

To generate PI4KB knockout cells, NIH/3T3 cells were transfected with the successfully cloned plasmids. Cas9-GFP plasmids containing sequences that target sites in exon 1 and exon 3 where transfected in pairs (2a + 4b, 5a + 5b, 7a + 8b) to induce large deletions. After 48 h incubation at 37 °C and 5% CO_2, cells were trypsinized, collected in growth medium, centrifuged, washed with PBS and filtered through a FACS tube cap filter. Cells were then sorted for GFP signal using the FACS Aria Fusion Flow Cytometry System (488/445 laser line) to only obtain successfully transfected cells. The top 10% of GFP-positive cells were re-plated into a 10 cm dish at a very high dilution to allow growth of single clones. Cells at a higher density were seeded into a 6-well plate to use these bulk cells for optimization of screening primers.

Selected colonies were trypsinized, expanded and analysed for successful PI4KB knockout using an anti-PI4KB antibody after cell lysis and immunoblotting. Clones that were validated on protein level were expanded, cryo-conserved and used for further experiments.

Detection of GLI3 by means of immunoblotting

NIH/3T3 cells were seeded in 6-well plates (Sarstedt, #83.3920, $1x10^5$ cells/well). On the next day, cells were starved in assay medium for 8 h and then treated with 2 µM Purmorphamine and the compound or DMSO as a control for 16 h. Cells were then lysed in SDS sample buffer without DTE and bromophenol blue (0.5 M Tris, 40% Glycerol, 8% SDS in mH_2O). After sonification, protein concentration was determined using the DC protein assay kit (Bio-Rad #5000112) and a BSA standard. Then, 0.8 mM DTE and 0.2% Bromophenol blue were added and the samples were boiled at 95°C for 5 min. Proteins were separated via SDS page, transferred via wet blotting onto PVFD membranes (Millipore, # IPFL00010), which were blocked in 5% milk in TBS for 1 h at room temperature. The mouse anti-GLI3 antibody (clone 6F5) was a kind gift from Suzie Scales [15] and was applied at 1:500 dilution in blocking buffer at 4°C overnight. As a reference gene, β-Actin was detected using a rabbit anti- β-Actin antibody (abcam, #ab8227) at 1:5000 dilution in LI-COR blocking buffer (LI-COR Biosciences, #927-40000) at 4°C overnight. Donkey anti-mouse-800CW and Donkey anti-rabbit-800CW secondary antibodies were added at 1:5000 dilution in LI-COR blocking buffer at RT for 1 h to detect the proteins of interest using an Odyssey Fc imaging system (LI-COR Biosciences). Band intensities were quantified with the image studio software (Version 4.0.21, LI-COR Biosciences, ©2014), divided by the β-Actin band intensity and normalized to DMSO treated cells.

ActiveX affinity chromatography

The enrichment of ATP binding proteins was carried out using the Pierce™ Kinase Enrichment Kit with ATP Probe (Thermo Fisher Scientific, # 88310) according to the manufacturer's instructions. Briefly, 1 mg of NIH/3T3 desalted lysate was incubated with 1 M $MgCl_2$ for 1 min at room temperature (RT) followed by addition 100 and 200 µM (immunoblot detection) or 200 µM (mass spectrometry detection) compound (or 1% DMSO as a control) and subsequent incubation for 1 h at room temperature. Then, 5 µM of the desthiobiotin ATP probe was added to each sample and incubated for 10 min at RT. Next, the streptavidin agarose beads were added, and the samples were incubated at room temperature on an overhead rotor for 1 h. After three washing steps with IP lysis buffer provided with the kit, the bound proteins were eluted by addition of 5xSDS sample buffer (0.5 M Tris, 40% glycerol, 8% SDS in mH_2O) and incubation at 95°C for 5 min. Proteins were either detected by immunoblotting employing a rabbit anti-PI4KB antibody (Millipore, #06-578) at 1:1000 dilution in 3% milk in PBS at 4°C overnight (secondary antibodies and detection as described for the GLI3 immunoblotting procedure) or digested and detected via mass spectrometry.

In-gel digestion and mass spectrometry detection after ActiveX affinity chromatography

Protein samples were loaded on a precast TGX 4-20% gel (Bio-Rad, #4561094), the gel was run for approx. 15-30 min at 80 V until the complete sample was inside the gel. The lanes were cut accordingly and the single gel pieces were fixed in fixation solution (H_2O:EtOH:Acetic acid = 5:4:1) overnight. After subsequent washing steps, reduction (50 mM DTT in 25 mM NH_4HCO_3 for 45 min at 600 rpm, 37°C) and alkylation (55 mM iodacetamide in 25 mM NH_4HCO_3 for 1 h at RT in the dark), the gel pieces were washed again and dehydrated with Acetonitrile before they

were dried for 30 min at room temperature. Subsequently, digest solution (0.1 µg/µl Trypsin in 10 mM HCl diluted 1/10 in 25 mM NH_4HCO_3) was added to the dried gel and incubated overnight at 350 rpm and 30°C. On the next day, concentrated TFA was added to stop the digestion reaction. To extract the peptides from the gel, all samples were sonicated on ice for 30 min. Acetonitrile was added and the samples were incubated for 15 min at 350 rpm and 25°C. After one repetition, the supernatant was concentrated in a vacuum centrifuge for 3-6 h at 30°C. The protein fragments were analyzed by nano-HPLC-MS/MS using an UltimateTM 3000 RSLC nano-HPLC system and a Q ExactiveTM Plus or Q ExactiveTM HF Hybrid Quadrupole-Orbitrap mass spectrometer equipped with a nano-spray source (all Thermo Fisher Scientific). For this, the lyophilized tryptic peptides were dissolved in 20 µl 0.1% TFA and 3 µl of these samples were injected and enriched onto a C18 PepMap 100 column (5 µm, 100 Å, 300 µm ID * 5 mm) using 0.1% TFA and a flow rate of 30 µl/min for 5 min. Afterwards, the peptides were separated on a C18 PepMap 100 column (3 µm, 100 Å, 75 µm ID * 25 cm) using a linear gradient starting with 95% solvent A/5% solvent B and increasing to 70.0% solvent A/30.0% solvent B in 90 min with a flow rate of 300 nl/min (solvent A: water containing 0.1% formic acid, solvent B: acetonitrile containing 0.1% formic acid). The nano-HPLC was coupled to the mass spectrometer using a standard coated Pico Tip emitter (ID 20 µm, Tip-ID 10 µM). A mass range of m/z 300 to 1,650 was acquired with a resolution of 70,000 for a full scan, followed by up to ten high energy collision dissociation (HCD) MS/MS scans of the most intense at least doubly charged ions with a resolution of 17,500 for the samples analyzed on the Q Exactive Plus. For samples analyzed on the Q Exactive HF the same mass range was used and a resolution of 60,000 for a full scan, followed by up to fifteen HCD-MS/MS scans of the most intense at least doubly charged ions with a resolution of 16,000. Protein identification and relative quantification were performed using MaxQuant [16] v.1.5.3.30 or v.1.5.6.5, respectively, including the Andromeda search algorithm and searching the mouse reference proteome of the uniprot database. Briefly, a MS/MS ion search was performed for full enzymatic trypsin cleavages

allowing two miscleavages. For protein modifications carbamidomethylation was chosen as fixed and oxidation of methionine and acetylation of the N-terminus as variable modifications. The mass accuracy was set to 20 ppm for the first and 6 ppm for the second search. The false discovery rates for peptide and protein identification were set to 0.01. Only proteins for which at least two peptides were quantified were chosen for further validation. Relative quantification of proteins was carried out using the label-free quantification algorithm implemented in MaxQuant. All experiments were performed in technical triplicates. Statistical data analysis was performed using Perseus [17], Label-free quantification (LFQ) intensities were logarithmized (log2) and samples resulting from affinity purification using the small molecule as a competitor were grouped together and samples resulting from affinity purification using DMSO as solvent control as well. Proteins which were not at least three times quantified in at least one of the groups were filtered off. Missing values were imputed using small normal distributed values and a t-test was performed. Proteins which were statistically significant (FDR: 0.05) outliers and enriched in the samples treated with DMSO over the ones treated with compound competitor were considered as hits.

Cellular Thermal Shift Assay (CETSA)

The compound (or DMSO as a control) was added to 900 µg NIH/3T3 lysate at a concentration of 50 µM for 10 min at room temperature. The lysate was then aliquoted in nine PCR tubes and heated to several temperatures (40.3, 44.5, 50.6, 54, 55.6, 57.1, 58.6, 63.7 and 66.9°C) for 3 min. After heating, the samples were incubated at RT for another 3 min and transferred to ultracentrifuge vials. Ultracentrifugation was carried out at 100,000 g and 4°C for 25 min. The supernatant was supplemented with 1x SDS sample buffer and heated to 95°C for 5 min. The levels of soluble PI4KB were determined by means of immunoblotting as described for the ActiveX affinity chromatography. The band intensities were quantified with the Image studio

software (Version 4.0.21, LI-COR Biosciences, ©2014) and plotted against the temperature followed by non-linear regression using a four-parameter fit (GraphPad Prism 6, GraphPad Software, USA) to calculate the melting temperature.

Biochemical kinase measurements

If not stated otherwise, all kinase measurements were performed by SelectScreen (Thermo Fisher Scientific). All lipid kinases were measured in the Adapta activity assay that monitors ADP formation via a fluorescence resonance energy transfer (FRET)-based ATP-depletion reaction. The majority of kinases measured in the panel was subjected to the Z'Lyte activity assay that measures kinase activity by monitoring the conversion of a synthetic peptide substrate in a FRET-based manner. The Lantha binding assay is a FRET based displacement assay. For MYLK, only the Lantha binding assay was available at SelectScreen. To assess compound influence on kinase activity, we employed the radiometric activity assay offered by Reaction biology. In this assay, ^{33}P-ATP is added to the kinase reaction and after 2 h at RT, the reaction mixture is spotted onto P81 ion exchange paper to separate kinase substrate, which has been phosphorylated with radioactive phosphate, and ^{33}P-ATP. The amount of radiolabeled substrate is then measured by scintillation counting. For all kinase assays, data were normalized to DMSO treated samples.

Mode of inhibition study

The luminescent ADP-Glo assay was performed at SignalChem to measure ATP consumption as per the manufacturer's instructions. Full-length recombinant human PI4KB was co-expressed at SignalChem by baculovirus in Sf9 insect cells using an N-terminal GST tag. For the assay, different concentrations of compound were titrated against different ATP concentrations.

Relative light units (RLU) were converted to ATP conversion rate using standard ATP conversion curves. Reaction velocities were calculated based on the amount of ATP transferred in the reactions. For determination of the mode of inhibition, Km and Vmax were determined using the equation Y= Vmax*X/(Km+X) using GraphPad Prism. Alternatively, the inverse of the ATP concentration was plotted against the inverse velocity in a Lineweaver-Burk plot.

Turbidimetric solubility assay

Various concentrations of Pipinib (0-200 µM) were incubated for 1 h in DMEM+ 10% FBS at 37°C and 5% CO_2 or in the Active X buffer (25 mM Tris-HCl, pH 7.4, 150 mM NaCl, 1 mM EDTA, 1 % NP-40, 5 % glycerol) at room temperature. Absorbance at 620 nm was then determined as a measure of compound precipitation/aggregation.

5. Experimental procedures - Chemical synthesis

The chemicals used were purchased from Alfa Aesar and Sigma Aldrich and were used as received without any further purification. Thin layer chromatography (TLC) was performed on silica gel aluminium plates with F-254 indicator (Merck, Germany). Reactants and products were visualized on the TLC plates by irradiation with UV light. Silica gel (Across 60A, particle size 0.035 – 0.070) was employed for column chromatography purifications. ^{1}H NMR spectra for the reported compounds were recorded on Bruker DRX400 (400 MHz), Bruker DRX500 (500 MHz) and INOVA500 (500 MHz) instruments using deuterated DMSO (DMSO-d6) or chloroform ($CDCl_3$) as internal standard. The following abbreviations were used for ^{1}H-NMR signal characterizations: s (singlet), d (doublet), dd (double doublet), dt (double triplet), t (triplet), q (quartet) and m (multiplet). Coupling constants are reported in Hertz (Hz). Fourier transform infrared spectroscopy (FT-IR) analyses were recorded on a Bruker Tensor 27 spectrometer (ATR, neat) and are presented as frequency of absorption (cm^{-1}). High resolution mass spectrometry (HRMS) analyses were recorded on an LTQ Orbitrap mass spectrometer coupled to an Accela HPLC-System (HPLC column: Hypersyl GOLD, 50 mm × 1 mm, 1.9μm). Eluent mixtures for TLC are depicted as volume : volume. EtOAc represents ethyl acetate whereas PE is the abbreviation for petroleum ether. ^{t}R stands for retention time on TLC plate. Chemical yields denote the isolated and pure substances.

General procedure for the synthesis of Pipinib (entry 1) and entries 2-16

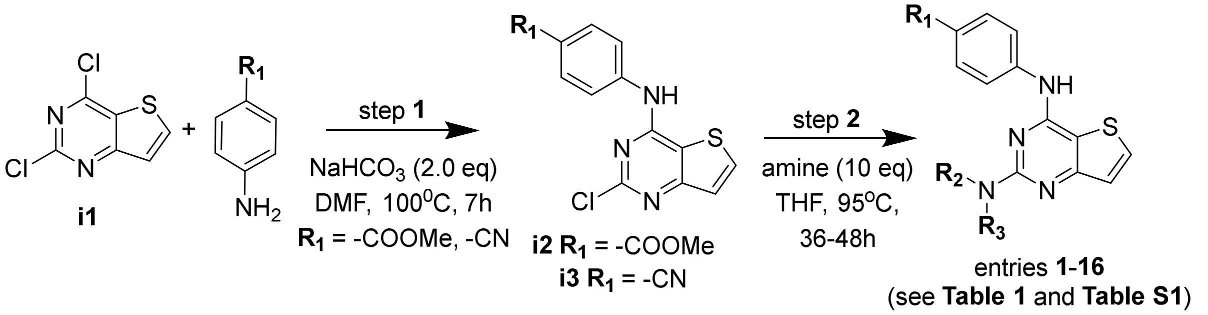

Intermediate 1 (**i1**) has been synthesized according to literature procedure[3]. Intermediates 2-3 (**i2-i3**) have been synthesized similarly a previously reported experimental procedure.[8] However, in our hands the reaction proceeded superior in DMF instead of CH_3CN and was performed as follows (step **1**): **i1** (2.5 mmol, 510 mg, 1 eq), methyl 4-aminobenzoate (3.75 mmol, 566.9 mg, 1.5 eq) and $NaHCO_3$ (5 mmol, 420 mg, 2 eq) were dissolved in 2.5 mL DMF and reacted at 100°C until no starting material has been detected by TLC (usually overnight). Then, DMF was removed and the resulting oily product was precipitated by adding 5 mL water. The solid material was washed 3 x 5 mL MeOH and dried to yield a pale yellow solid (yield = 56%) which was subsequently used in the next step without any further purification. Compound identity has been assessed using LC-MS. Entries **1-11** have been synthesized following previously published methods.[9] In our hands, changing the solvent to THF lead to superior yields and therefore followed the general procedure: to **i2** (28.6 mg, 0.1 mmol, 1 eq) has been added the corresponding primary or secondary amine (1 mmol, 10 eq) in THF (0.3 mL). The mixture was let to react in a sealed tube at 94°C for 36-48 h while monitoring the reaction progression using TLC. The reaction was stopped, concentrated *in vacuo* and purified using column chromatography on silica (eluent: EtOAc : PE = 1:2 to EtOAc : PE = 2:1). The same experimental procedure has been applied to entries **12-16**. Note: entries **1** and **12** were synthesized in the absence of THF, using neat isopropylamine.

Spectral characterization of Pipinib (entry 1) was fully assessed by ^{1}H- and ^{13}C-NMR as well as FT-IR, LC-MS and HRMS, respectively and is representative for all other derivatives reported in

this study. Since following previously reported experimental procedures, the identity of **i2**-**i16** has been verified by ^{1}H-NMR, FT-IR and HRMS only (section 5.5). Compounds' purities have been initially assessed upon completion of their synthesis by means of LC-MS. All derivatives were homogeneous and exhibited purities above 95%. A representative HPLC chromatogram of Pipinib (entry **1**) is shown in section 5.4. Compounds were stored as stock solutions in 100% DMSO at -20°C. Moreover, all compounds proved to be bench stable upon incubation at room temperature for 24 h as 10 mM stock solutions in 100% DMSO as indicated by their LC-MS profiles. Prior each cell-based high-throughput screening, compounds' purities have been verified independently by the screening facility, thus confirming that multiple thaw-freezing cycles did not affect the stability of the compound. Consequently, only homogeneous compounds exhibiting purities above 95% were included in the cell-based screening process. Moreover, i*n vitro* and *in cellulo* experiments with Pipinib (entry **1**) have been performed with multiple batches of stock solutions in 100% DMSO furnishing identical data sets further confirming the chemical stability of the compound.

Methyl 4-((2-(isopropylamino)thieno[3,2-d]pyrimidin-4-yl)amino)benzoate

Pipinib (entry **1**); Yield = 37%; ^{t}R = 0.19 (EtOAc:PE=1:1)

^{1}H-NMR [500 MHz, DMSO-d6] δ: 10.83 (b, 1H), 8.42 (b, 1H), 8.30 (d, J = 5.3 Hz, 1H), 8.00 (b, 4H), 7.27 (d, J = 5.3 Hz, 1H), 4.05 (b, J = 12.3 Hz, 1H), 3.86 (s, 3H), 1.24 (d, J = 6.5 Hz, 6H).

^{13}C NMR [124 MHz, DMSO-d6] δ: 165.80, 159.00, 158.74, 129.95, 122.26, 52.15, 22.09.

FT-IR (cm^{-1}): 3079, 2976, 1717, 1674, 1641, 1589, 1555, 1522, 1465, 1436, 1418, 1379, 1280, 1200, 1179, 1136, 1017, 857, 831, 798, 770, 722, 694, 674, 661, 646, 629, 617.

HRMS Calculated for $C_{17}H_{19}N_4O_2S$: m/z = 343.12232 [M+H$^+$], found: 343.12267.

5.1. PI4KA inhibitor

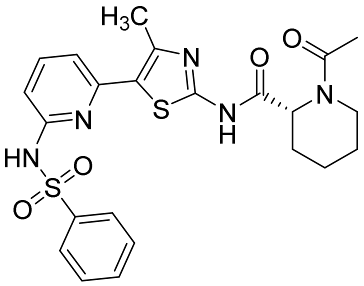

AZ7 was obtained as a kind donation by Astra Zeneca[18] and was later on purchased from Ximbio.

5.2. PIK3C2G inhibitors[19]

*N*²-(3,5-dimethoxyphenyl)pyrazine-2,3-diamine

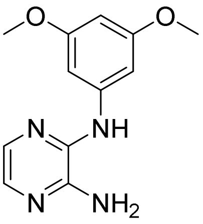

A suspension of 3-chloropyrazin-2-amine (500 mg, 3.86 mmol) and 3,5-dimethoxyaniline (621 mg, 4.05 mmol) in 20 mL HCl (aq., 0.1 M) was heated in the micro wave to 120°C for 2 hours. After bringing the reaction mixture to room temperature it was diluted and thereafter stirred for 5 minutes with 20 mL EtOAC before the two layers were separated. The aqueous layer was extracted three times with in each case 20 mL EtOAc and the combined organic layers were dried over anhydrous magnesium sulfate. The crude was purified by flash column chromatography on silica gel (10-60% EtOAc / Cyclohexane) to yield the product (293 mg, 1.19 mmol, 31%) as an off-white wax.

^{1}H-NMR [600 MHz, $CDCl_3$] δ: 7.69 (d, *J* = 2.8 Hz, 1H), 7.64 (d, *J* = 2.9 Hz, 1H), 6.52 (d, *J* = 2.0 Hz, 2H), 6.17 (t, *J* = 2.0 Hz, 1H), 4.43 (s, 2H), 3.78 (s, 6H).

^{13}C-NMR [151 MHz, $CDCl_3$] δ: 161.45, 145.44, 141.94, 140.96, 133.96, 132.85, 97.86, 94.87, 55.50.

HRMS Calculated for $C_{12}H_{15}O_2N_4$: m/z = 247.11895 [$M+H^+$], found: 247.11874.

N-(3-((3,5-dimethoxyphenyl)amino)pyrazin-2-yl)-3-nitrobenzenesulfonamide (Compound 17)

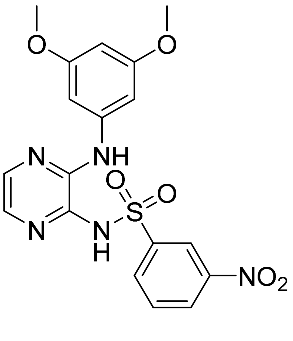

N-(3-((3,5-dimethoxyphenyl)amino)pyrazin-2-yl)-3-nitrobenzene sulfonamide was synthesized as described in the literature.[19]

^{1}H-NMR [500 MHz, $CDCl_3$] δ: 12.02 (s, 1H), 8.82 (s, 1H), 8.41 (d, *J*=6.9, 1H), 8.32 (d, *J*=6.8, 1H), 8.11 (s, 1H), 7.73 (t, *J*=7.5, 1H), 7.46 (s, 1H), 6.94 (d, *J*=1.7, 2H), 6.91 (s, 1H), 6.22 (s, 1H), 3.79 (s, 6H).

^{13}C-NMR [126 MHz, $CDCl_3$] δ: 161.19, 148.29, 146.81, 144.39, 140.32, 139.61, 132.04, 130.61, 128.26, 127.17, 121.72, 113.21, 98.52, 96.20, 55.58.

HRMS Calculated for $C_{18}H_{18}O_6N_5S$: m/z = 432.09723 [M+H$^+$], found: 432.09694.

***N*-(3-(*N*-(3-((3,5-dimethoxyphenyl)amino)pyrazin-2-yl)sulfamoyl)phenyl) acetamide (Compound 18)**

N-(3-(N-(3-((3,5-dimethoxyphenyl)amino)pyrazin-2-yl)sulfamoyl)phenyl)acetamide was synthesized as described in the literature.[19]

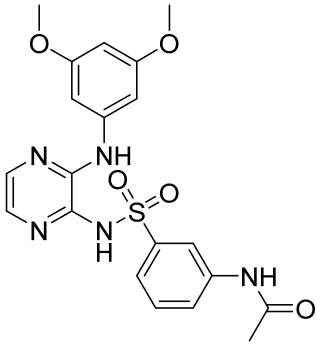

^{1}H-NMR [500 MHz, $CDCl_3$] δ: 11.91 (s, 1H), 8.15 (d, *J* = 6.3 Hz, 1H), 7.84 (d, *J* = 8.1 Hz, 1H), 7.76 (s, 1H), 7.72 – 7.63 (m, 1H), 7.50 – 7.42 (m, 1H), 7.36 (d, *J* = 4.1 Hz, 1H), 6.94 (d, *J* = 2.1 Hz, 2H), 6.83 (d, *J* = 3.7 Hz, 1H), 6.20 (t, *J* = 2.0 Hz, 1H), 3.78 (s, 6H), 2.18 (s, 3H).

HRMS Calculated for $C_{20}H_2O_5N_5S$: m/z = 444.13362 [M+H$^+$]; found: 444.13320.

methyl 3-(*N*-(3-((3,5-dimethoxyphenyl)amino)pyrazin-2-yl)sulfamoyl)benzoate (Compound 19)

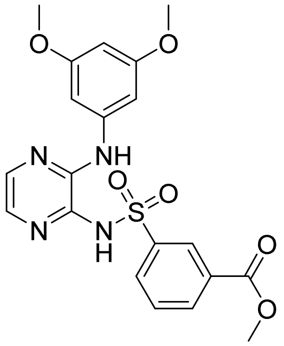

methyl 3-(N-(3-((3,5-dimethoxyphenyl)amino)pyrazin-2-yl)sulfamoyl)benzoate was synthesized as described in the literature.[19]

^{1}H-NMR [400 MHz, $CDCl_3$] δ = 11.97 (s, 1H), 8.63 (s, 1H), 8.24 (d, J = 7.8 Hz, 1H), 8.21 – 8.09 (m, 2H), 7.61 (t, J = 7.8 Hz, 1H), 7.41 (d, J = 4.2 Hz, 1H), 6.96 (d, J = 2.2 Hz, 2H), 6.83 (d, J = 4.1 Hz, 1H), 6.22 (t, J = 2.1 Hz, 1H), 3.95 (s, 3H), 3.79 (s, 6H)

^{13}C-NMR [126 MHz, CDCl3] δ = 161.16, 147.15, 146.90, 143.03, 140.55, 139.95, 132.27, 130.11, 128.72, 127.09, 119.03, 115.93, 112.12, 98.39, 96.06, 55.58, 31.10.

HRMS Calculated for $C_{20}H_2O_5N_5S$: m/z = 445.11763 $[M+H]^+$; found: 445.11635.

5.3. Derivatives of Pipinib (entry 1)

Compounds **15-23 (Supplementray Table S1)** were obtained from BioFocus, UK.

Methyl 4-((2-(allylamino)thieno[3,2-d]pyrimidin-4-yl)amino)benzoate

(entry **2**)

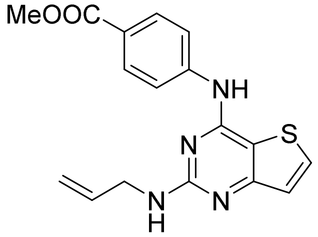

Yield = 26%
^tR = 0.18 (EtOAc:PE=1:1)

^{1}H-NMR [400 MHz, DMSO-d6] δ: 10.81 (b, 1H), 8.42 (b, 1H), 8.30 (d, J = 4.8 Hz, 1H), 7.99 (s, 4H), 7.28 (d, J = 5.4 Hz, 1H), 5.96 (ddt, J = 17.1, 10.1, 5.0 Hz, 1H), 5.21 (s, 1H), 5.15 (dd, J = 10.3, 1.5 Hz, 1H), 4.02 (s, 2H), 3.85 (s, 3H);

FT-IR (cm^{-1}): 3093, 1718, 1677, 1655, 1618, 1560, 1523, 1468, 1438, 1384, 1281, 1202, 1140, 838, 772, 723, 672, 667.

HRMS Calculated for $C_{17}H_{17}N_4O_2S$: m/z = 341.10667 [M+H$^+$], found: 341.10779.

Methyl 4-((2-(isobutylamino)thieno[3,2-d]pyrimidin-4-yl)amino)benzoate

(entry **3**)

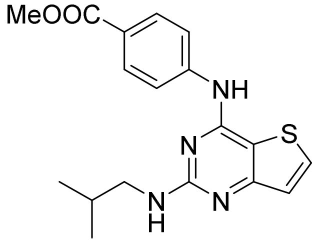

Yield = 47%

^tR = 0.32 (EtOAc:PE=1:1)

^{1}H-NMR [400 MHz, DMSO-d6] δ: 10.91 (b, 1H), 8.58 (b, 1H), 8.31 (d, J = 4.2 Hz, 1H), 7.98 (s, 4H), 7.27 (s, 1H), 3.86 (s, 3H), 3.20 (t, J = 6.0 Hz, 2H), 1.98 – 1.87 (m, 1H), 0.92 (d, J = 6.5 Hz, 6H).

FT-IR (cm^{-1}): 3271, 3081, 2959, 1717, 1674, 1644, 1618, 1587, 1557, 1522, 1466, 1436, 1419, 1383, 1279, 1251, 1199, 1179, 1136, 1017, 964, 834, 798, 769, 722, 694, 662, 645.

HRMS Calculated for $C_{18}H_{21}N_4O_2S$: m/z = 357.13797 [M+H$^+$], found: 357.13818.

Methyl 4-((2-(cyclobutylamino)thieno[3,2-d]pyrimidin-4-yl)amino)benzoate

(entry **4**)

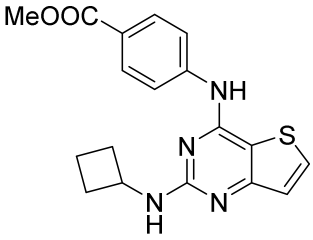

Yield = 67 %
^tR = 0.26 (EtOAc:PE=1:1)

^{1}H-NMR [500 MHz, DMSO-d6] δ: 10.76 (b, 1H), 8.72 (b, 1H), 8.29 (d, J = 5.2 Hz, 1H), 8.01 (s, 4H), 7.28 (d, J = 2.1 Hz, 1H), 4.32 (b, 1H), 3.86 (s, 3H), 2.32 (b, 2H), 2.12 – 2.01 (m, 2H), 1.79 – 1.69 (m, 2H).

FT-IR (cm^{-1}): 3276, 3079, 2951, 1714, 1674, 1639, 1592, 1555, 1521, 1466, 1435, 1380, 1280, 1200, 1180, 1141, 1116, 1018, 839, 798, 769, 722, 674, 662, 646, 630, 616.

HRMS Calculated for $C_{18}H_{19}N_4O_2S$: m/z = 355.12232 [M+H$^+$], found: 355.12277.

Methyl 4-((2-((cyclopropylmethyl)amino)thieno[3,2-d]pyrimidin-4-yl)amino)

benzoate (entry **5**)

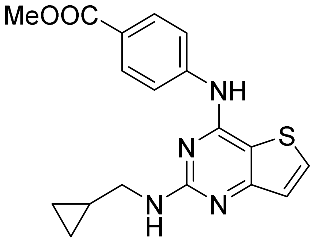

Yield = 36 %

^tR = 0.37 (EtOAc:PE=1:1)

^{1}H-NMR [500 MHz, DMSO-d6] δ: 10.87 (b, 1H), 8.44 (b, 1H), 8.30 (d, J = 4.7 Hz, 1H), 8.00 (s, 4H), 7.28 (d, J = 5.3 Hz, 1H), 3.86 (s, 3H), 3.27 (b, 2H), 1.18 – 1.03 (m, 1H), 0.48 (d, J = 7.5 Hz, 2H), 0.26 (d, J = 4.5 Hz, 2H).

FT-IR (cm^{-1}): 3092, 3014, 1711, 1676, 1636, 1577, 1557, 1521, 1468, 1422, 1378, 1280, 1187, 1146, 1118, 1048, 1018, 1001, 858, 840, 781, 750, 722, 694, 674, 662, 649, 629, 617.

HRMS Calculated for $C_{18}H_{19}N_4O_2S$: m/z = 355.12232 [M+H$^+$], found: 355.12305.

Methyl 4-((2-((2-methoxyethyl)amino)thieno[3,2-d]pyrimidin-4-yl)amino)

benzoate (entry **6**)

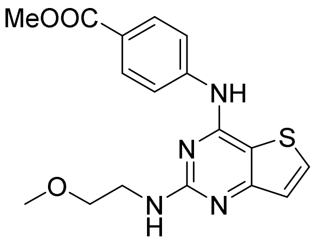

Yield = 29%

^{t}R = 0.1 (EtOAc:PE=1:1)

^{1}**H-NMR** [400 MHz, DMSO-d6] δ: 10.84 (b, 1H), 8.29 (s, 1H), 8.23 (b, 1H), 8.06 (b, 2H), 7.85 (d, J = 8.3 Hz, 2H), 7.29 (d, J = 5.4 Hz, 1H), 3.53 (s, 4H), 3.28 (s, 3H).

FT-IR (cm^{-1}): 2917, 2849, 2297, 2251, 1691, 1552, 1534, 1468, 1218, 1022, 772, 722, 674, 661, 646, 626, 617.

HRMS Calculated for $C_{16}H_{16}N_5OS$: m/z = 326.10701 [M+H$^+$], found: 326.10769.

Methyl 4-((2-(((tetrahydrofuran-2-yl)methyl)amino)thieno[3,2-d]pyrimidin-4-yl)

amino) benzoate (entry **7**)

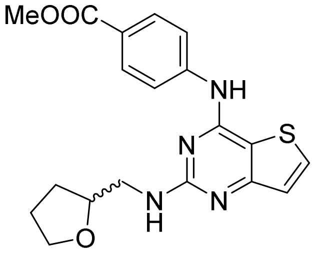

Yield = 42 %

^{t}R = 0.22 (EtOAc)

^{1}**H-NMR** [500 MHz, DMSO-d6] δ: 10.93 (b, 1H), 8.42 (b, 1H), 8.30 (b, 1H), 7.99 (b, 4H), 7.29 (d, J = 5.4 Hz, 1H), 4.09 – 4.00 (m, 1H), 3.86 (s, 3H), 3.77 (dd, J = 13.8, 6.9 Hz, 1H), 3.64 (dd, J = 14.4, 7.2 Hz, 1H), 3.53 – 3.39 (m, 2H), 1.93 (b, 1H), 1.87 – 1.77 (m, 2H), 1.60 (tt, J = 13.6, 7.0 Hz, 1H).

FT-IR (cm^{-1}): 3085, 2952, 1713, 1675, 1645, 1586, 1558, 1521, 1466, 1435, 1379, 1280, 1199, 1179, 1138, 1077, 1017, 967, 857, 833, 798, 769, 722, 693, 671, 664, 617.

HRMS Calculated for $C_{19}H_{21}N_4O_3S$: m/z = 385.13289 [M+H$^+$], found: 385.13304.

Methyl 4-((2-((cyclohexylmethyl)amino)thieno[3,2-d]pyrimidin-4-yl)amino)

benzoate (entry **8**)

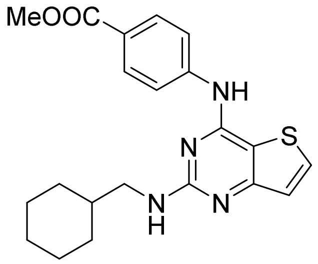

Yield = 47%

^{t}R = 0.31 (EtOAc:PE=1:1)

^{1}H-NMR [400 MHz, DMSO-d6] δ: 10.87 (b, 1H), 8.32 (d, J = 20.6 Hz, 2H), 7.98 (s, 4H), 7.28 (s, 1H), 3.86 (s, 3H), 3.21 (t, J = 6.1 Hz, 2H), 1.77 – 1.61 (m, 6H), 1.17 (m, 3H), 1.01 – 0.89 (m, 2H);

FT-IR (cm^{-1}): 3273, 3080, 2924, 2852, 1716, 1673, 1643, 1617, 1586, 1556, 1521, 1464, 1436, 1420, 1385, 1361, 1279, 1198, 1179, 1136, 1017, 964, 835, 798, 769, 722, 694, 674, 666, 661, 645.

HRMS Calculated for $C_{21}H_{25}N_4O_2S$: m/z = 397.16927 [M+H$^+$], found: 397.16802.

Methyl 4-((2-(pyrrolidin-1-yl)thieno[3,2-d]pyrimidin-4-yl)amino)benzoate

(entry **9**)

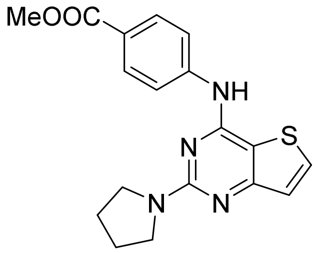

Yield = 24 %

^{t}R = 0.26 (EtOAc:PE=1:1)

^{1}H-NMR [500 MHz, DMSO-d6] δ: 10.80 (b, 1H), 8.31 (d, J = 5.3 Hz, 1H), 8.01 (s, 4H), 7.34 (d, J = 5.4 Hz, 1H), 3.85 (s, 3H), 3.61 (b, 4H), 2.01 (b, 4H).

FT-IR (cm^{-1}): 2934, 1674, 1639, 1616, 1578, 1557, 1510, 1467, 1419, 1367, 1345, 1281, 1200, 1180, 1131, 902, 799, 770, 718, 674, 666, 661, 645, 628, 617.

HRMS Calculated for $C_{18}H_{19}N_4O_2S$: m/z = 355.12232 [M+H$^+$], found: 355.12324.

Methyl 4-((2-(piperidin-1-yl)thieno[3,2-d]pyrimidin-4-yl)amino)benzoate
(entry **10**)

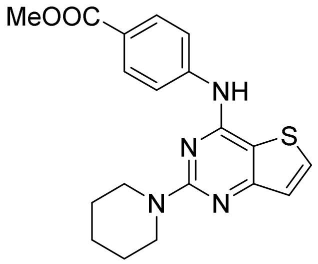

Yield = 42%

^{t}R = 0.29 (EtOAc:PE=1:3)

^{1}H-NMR [500 MHz, DMSO-d6] δ: 10.67 (b, 1H), 8.27 (d, J = 5.4 Hz, 1H), 8.00 (d, J = 8.7 Hz, 2H), 7.87 (d, J = 8.8 Hz, 2H), 7.30 (d, J = 5.4 Hz, 1H), 3.85 (s, 3H), 3.78 – 3.72 (m, 4H), 1.62 (b, 6H).

FT-IR (cm^{-1}): 2948, 1720, 1675, 1616, 1582, 1558, 1512, 1468, 1423, 1377, 1285, 1255, 1202, 1180, 1133, 1020, 890, 853, 800, 771, 719, 695, 668, 663, 646, 619.

HRMS Calculated for $C_{19}H_{21}N_4O_2S$: m/z = 369.13797 [M+H$^+$], found: 369.13904.

Methyl 4-((2-morpholinothieno[3,2-d]pyrimidin-4-yl)amino)benzoate
(entry **11**)

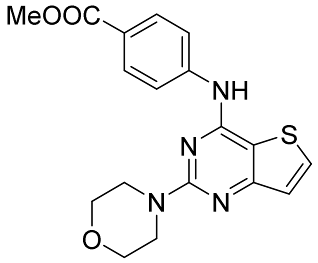

Yield = 57 %

^{t}R = 0.35 (EtOAc:PE=1:1)

^{1}H-NMR [400 MHz, DMSO-d6] δ: 10.45 (b, 1H), 8.23 (d, J = 5.4 Hz, 1H), 7.99 (d, J = 8.8 Hz, 2H), 7.89 (d, J = 8.8 Hz, 2H), 7.27 (d, J = 5.4 Hz, 1H), 3.84 (s, 3H), 3.72 (s, 8H).

FT-IR (cm^{-1}): 2952, 2863, 1717, 1674, 1653, 1614, 1580, 1557, 1511, 1470, 1437, 1405, 1375, 1279, 1246, 1200, 1179, 1117, 1066, 1017, 1007, 966, 894, 850, 827, 798, 771, 718, 694, 675, 667, 661, 645, 628, 617.

HRMS Calculated for $C_{18}H_{19}N_4O_3S$: m/z = 371.11724 [M+H$^+$], found: 371.11804.

4-((2-(isopropylamino)thieno[3,2-d]pyrimidin-4-yl)amino)benzonitrile

(entry **12**)

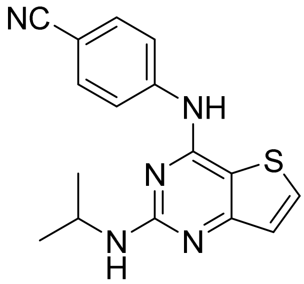

Yield = 29%

^{t}R = 0.26 (EtOAc:PE=1:1)

^{1}H-NMR [400 MHz, DMSO-d6] δ: 10.79 (b, 1H), 8.30 (d, J = 5.1 Hz, 2H), 8.06 (b, 2H), 7.87 (d, J = 8.5 Hz, 2H), 7.28 (d, J = 5.3 Hz, 1H), 4.05 (dd, J = 10.7, 5.4 Hz, 1H), 1.24 (d, J = 6.5 Hz, 6H).

FT-IR (cm^{-1}): 3278, 3066, 2969, 2916, 2251, 2230, 1651, 1618, 1588, 1549, 1511, 1462, 1415, 1378, 1299, 1254, 1236, 1200, 1176, 1127, 1098, 1055, 954, 900, 937, 794, 773, 717, 704, 674, 661, 646, 617.

HRMS Calculated for $C_{16}H_{16}N_5OS$: m/z = 310.11209 [M+H$^+$], found: 310.11298.

4-((2-(isobutylamino)thieno[3,2-d]pyrimidin-4-yl)amino)benzonitrile

(entry **13**)

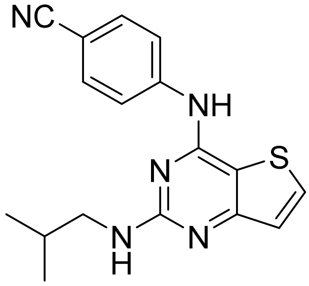

Yield = 29%

^{t}R = 0.30 (EtOAc:PE=1:1)

^{1}H-NMR [400 MHz, DMSO-d6] δ: 10.90 (b, 1H), 8.44 (b, 1H), 8.30 (b, 1H), 8.04 (b, 2H), 7.87 (d, J = 7.5 Hz, 2H), 7.28 (d, J = 4.6 Hz, 1H), 3.20 – 3.17 (m, 2H), 1.97 – 1.85 (m, 1H), 0.92 (d, J = 6.3 Hz, 6H).

FT-IR (cm^{-1}): 2251, 2018, 1651, 1219, 773, 722, 674, 661, 632, 617.

HRMS Calculated for $C_{17}H_{18}N_5S$: m/z = 324.12774 [M+H$^+$], found: 324.12777.

4-((2-(cyclobutylamino)thieno[3,2-d]pyrimidin-4-yl)amino)benzonitrile

(entry **14**)

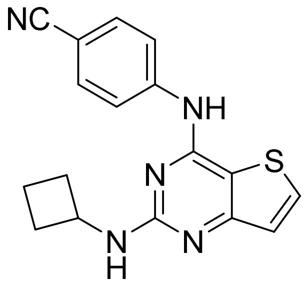

Yield = 33%

^{t}R = 0.27 (EtOAc:PE=1:1)

^{1}H-NMR [400 MHz, DMSO-d6] δ: 10.78 (b, 1H), 8.71 (b, 1H), 8.29 (d, J = 5.2 Hz, 1H), 8.07 (s, 2H), 7.88 (d, J = 7.9 Hz, 2H), 7.28 (d, J = 5.1 Hz, 1H), 4.37 – 4.22 (m, 1H), 2.32 (s, 2H), 2.13 – 1.97 (m, 2H), 1.73 (ddd, J = 18.5, 10.9, 7.7 Hz, 2H).

FT-IR (cm^{-1}): 2251, 2227, 1675, 1641, 1612, 1550, 1512, 1465, 1413, 1378, 1247, 1200, 1136, 837, 772, 722, 674, 666, 661, 646, 629, 617.

HRMS Calculated for $C_{17}H_{16}N_5S$: m/z = 322.11209 [M+H$^+$], found: 322.11276.

4-((2-((cyclopropylmethyl)amino)thieno[3,2-d]pyrimidin-4-yl)amino)benzonitrile

(entry **15**)

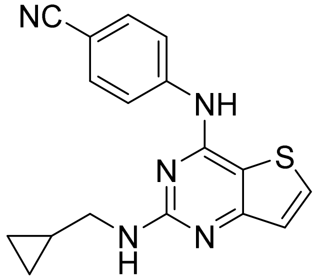

Yield = 16%

^{t}R = 0.26 (EtOAc:PE=1:1)

^{1}H-NMR [600 MHz, DMSO-d6] δ: 8.30 (s, 1H), 8.12 – 8.02 (m, 2H), 7.88 – 7.86 (m, 2H), 7.29 (d, J=5.2, 1H), 3.25 (s, 2H), 1.12 (s, 1H), 0.49 (s, 2H), 0.26 (s, 2H).

FT-IR (cm^{-1}): 3081, 2251, 2227, 1678, 1648, 1585, 1552, 1512, 1465, 1414, 1378, 1201, 1135, 836, 771, 722, 674, 666, 661, 646, 629, 617.

HRMS Calculated for $C_{17}H_{16}N_5S$: m/z = 322.11209 [M+H$^+$], found: 322.11271.

4-((2-((2-methoxyethyl)amino)thieno[3,2-d]pyrimidin-4-yl)amino)benzonitrile

(entry **16**)

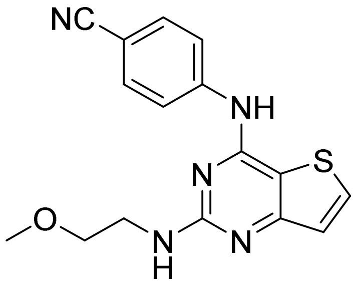

Yield = 29%

^{t}R = 0.1 (EtOAc:PE=1:1)

^{1}H-NMR [400 MHz, DMSO-d6] δ: 10.84 (b, 1H), 8.29 (s, 1H), 8.23 (b, 1H), 8.06 (b, 2H), 7.85 (d, J = 8.3 Hz, 2H), 7.29 (d, J = 5.4 Hz, 1H), 3.53 (s, 4H), 3.28 (s, 3H).

FT-IR (cm^{-1}): 2917, 2849, 2297, 2251, 1691, 1552, 1534, 1468, 1218, 1022, 772, 722, 674, 661, 646, 626, 617.

HRMS Calculated for $C_{16}H_{16}N_5OS$: m/z = 326.10701 [$M+H^+$] found: 326.10769.

5.4. Purity assessment of Pipinib (entry 1) by LC-MS.

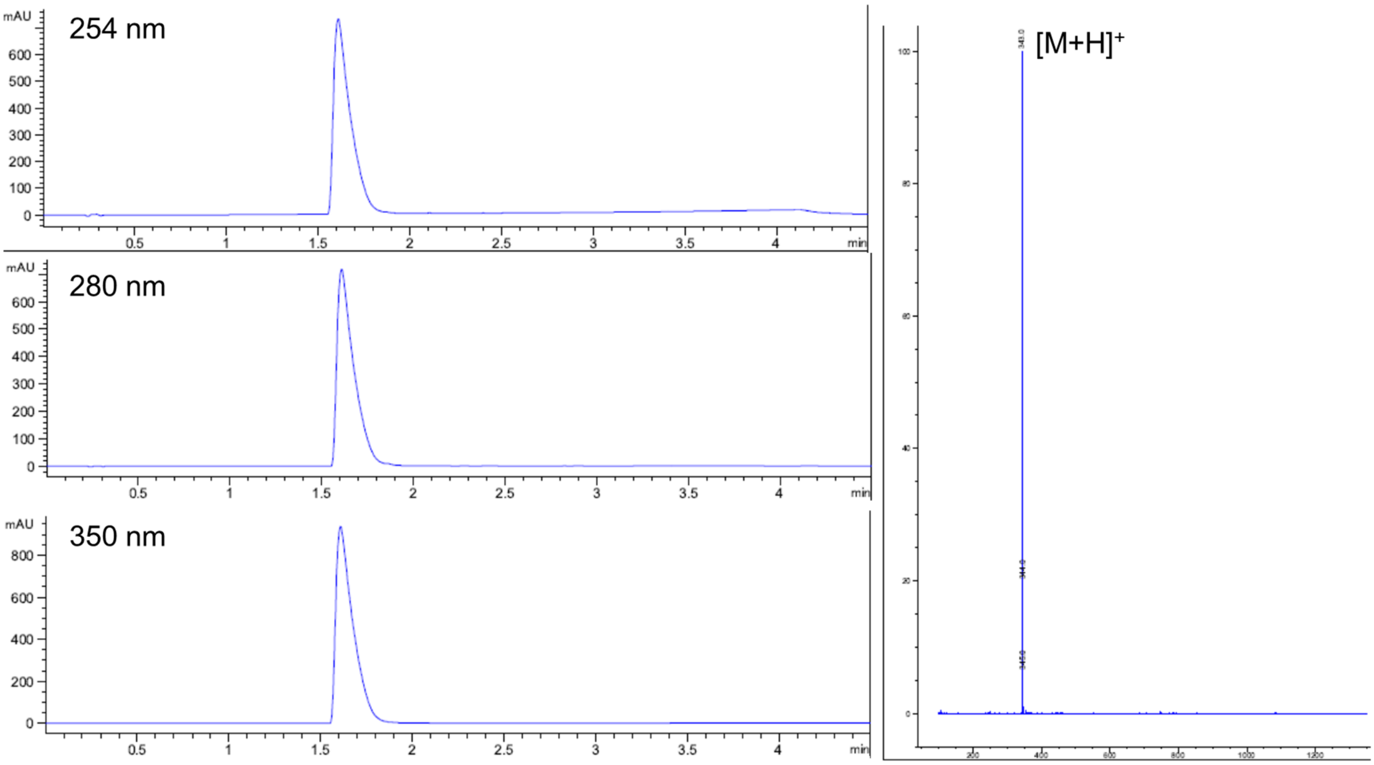

5.5. NMR Spectra

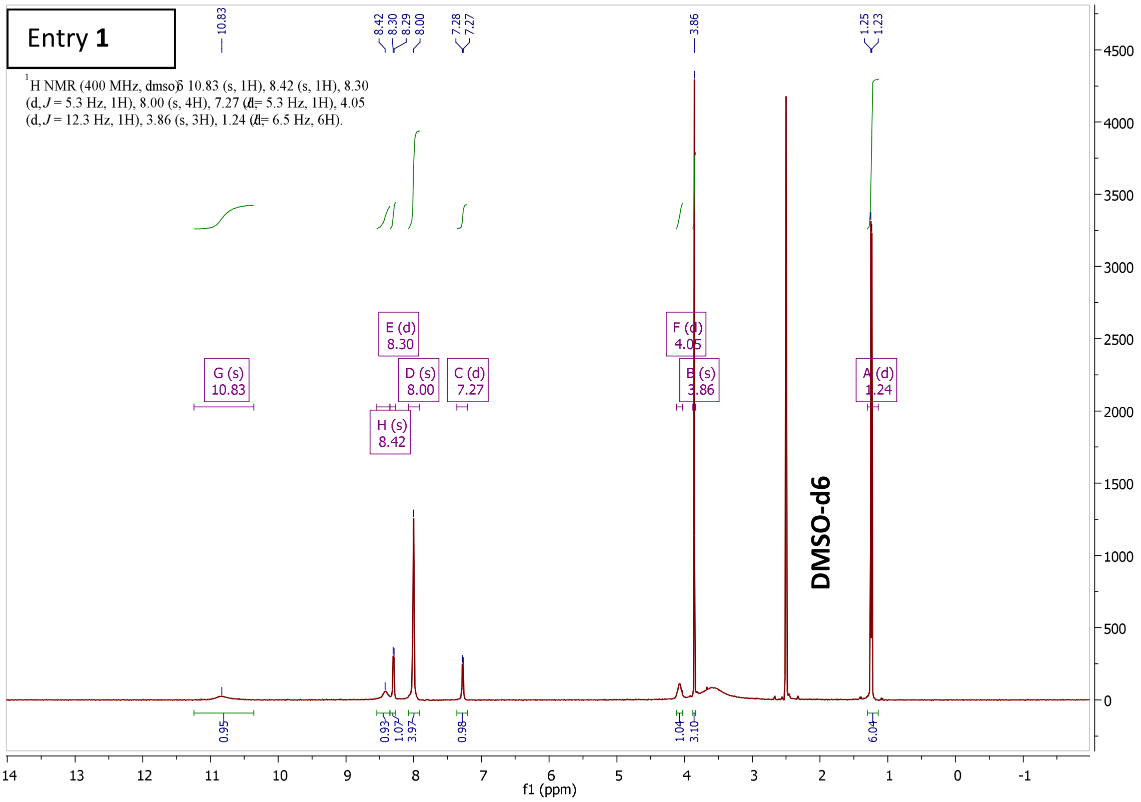

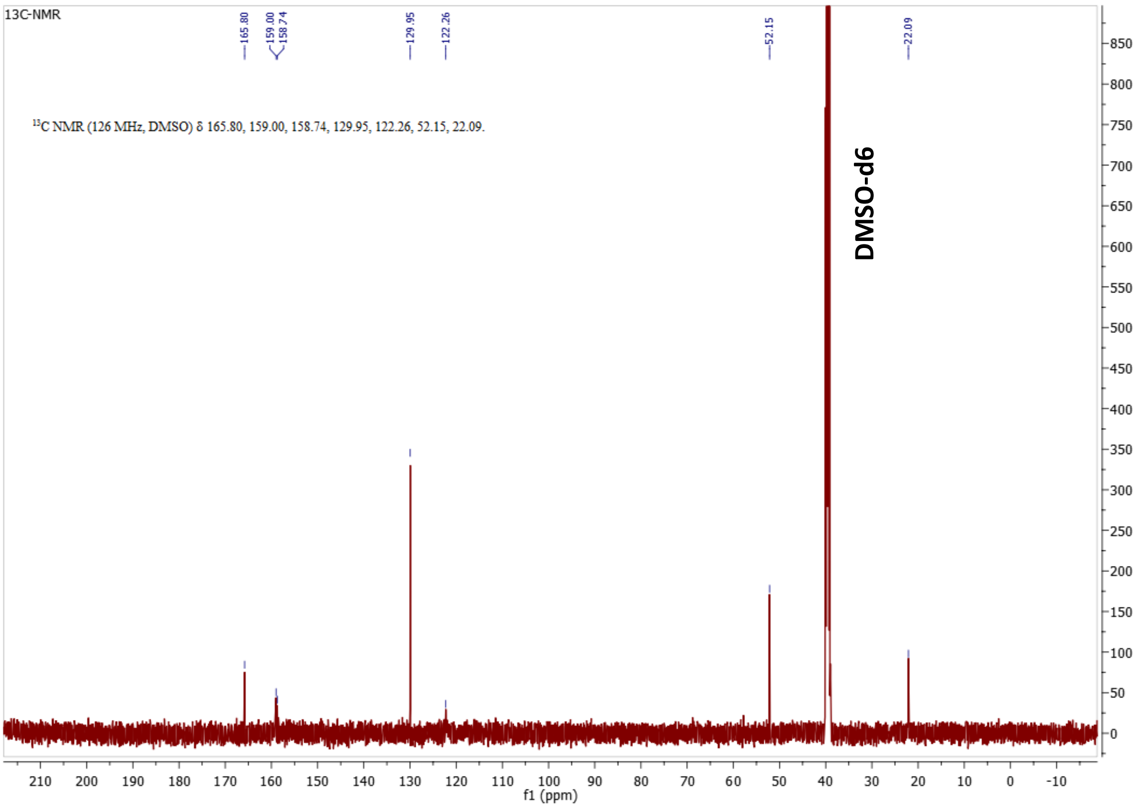

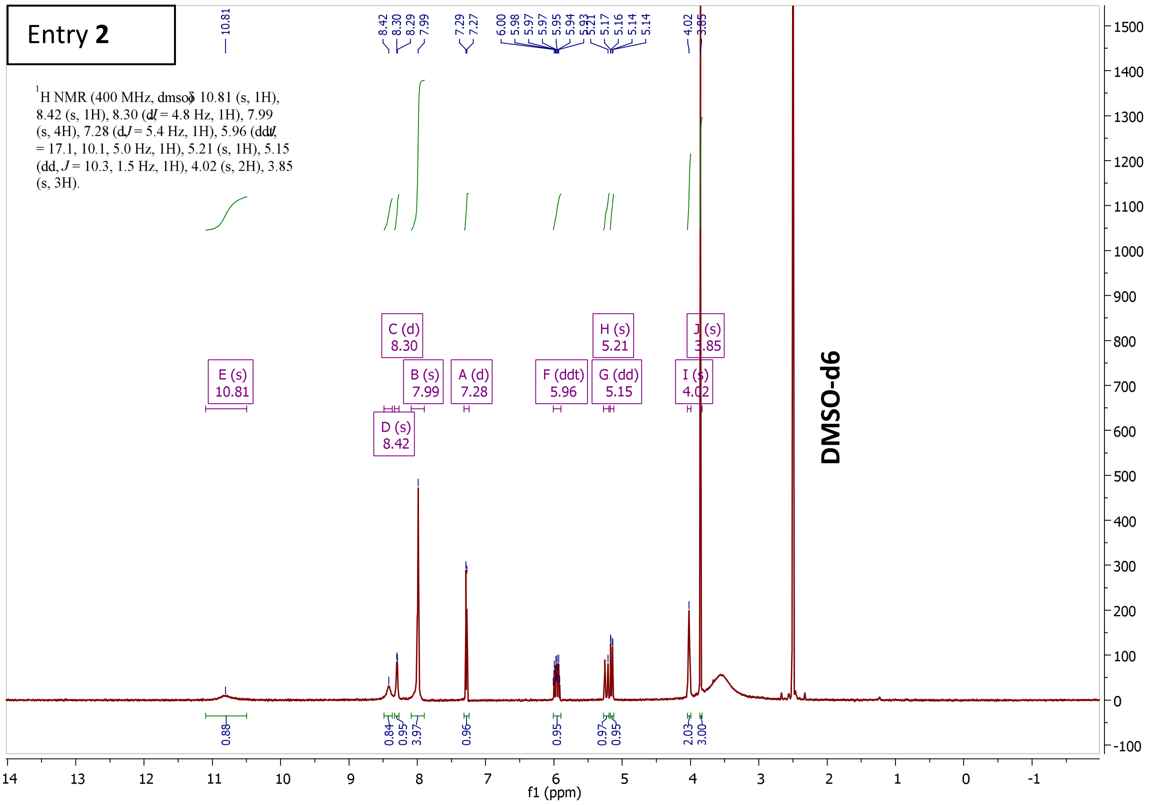

Entry 2
^{1}H NMR (400 MHz, dmso) δ 10.81 (s, 1H), 8.42 (s, 1H), 8.30 (d, J = 4.8 Hz, 1H), 7.99 (s, 4H), 7.28 (d, J = 5.4 Hz, 1H), 5.96 (ddt, J = 17.1, 10.1, 5.0 Hz, 1H), 5.21 (s, 1H), 5.15 (dd, J = 10.3, 1.5 Hz, 1H), 4.02 (s, 2H), 3.85 (s, 3H).
DMSO-d6
f1 (ppm)

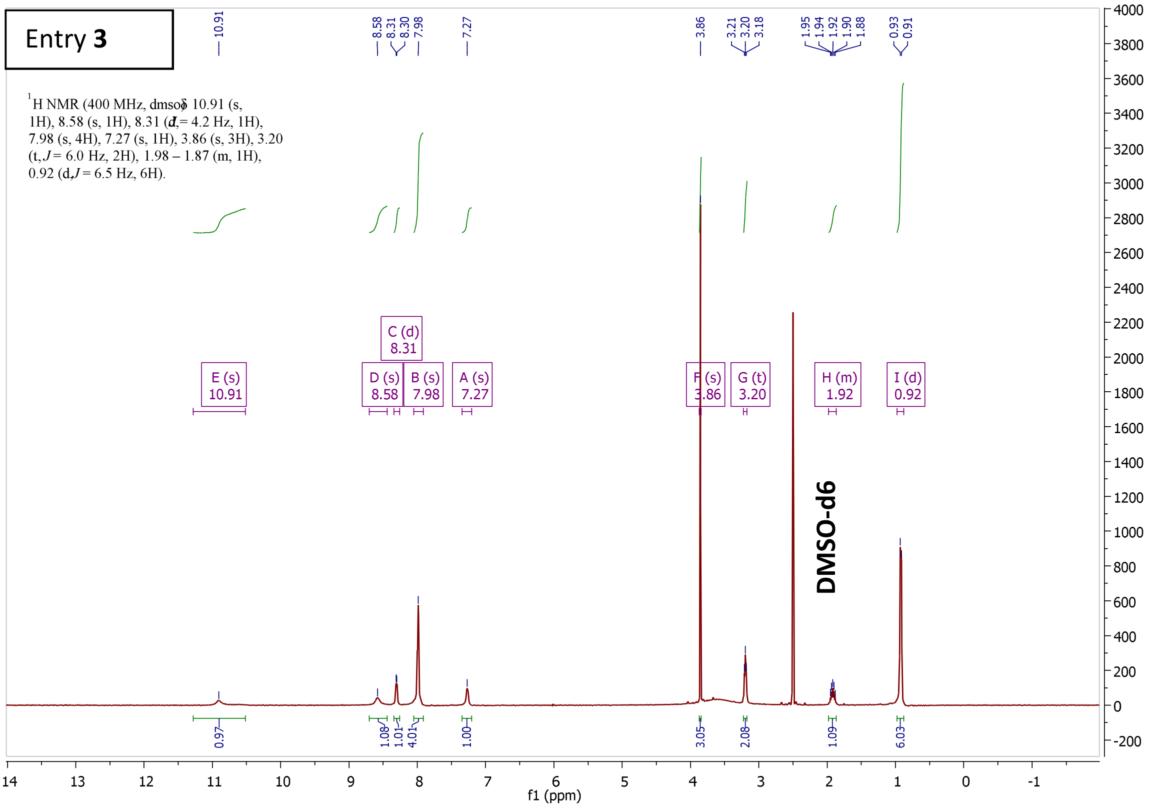

Entry 3
^{1}H NMR (400 MHz, dmso) δ 10.91 (s, 1H), 8.58 (s, 1H), 8.31 (d, J = 4.2 Hz, 1H), 7.98 (s, 4H), 7.27 (s, 1H), 3.86 (s, 3H), 3.20 (t, J = 6.0 Hz, 2H), 1.98 – 1.87 (m, 1H), 0.92 (d, J = 6.5 Hz, 6H).
DMSO-d6
f1 (ppm)

Entry **4**

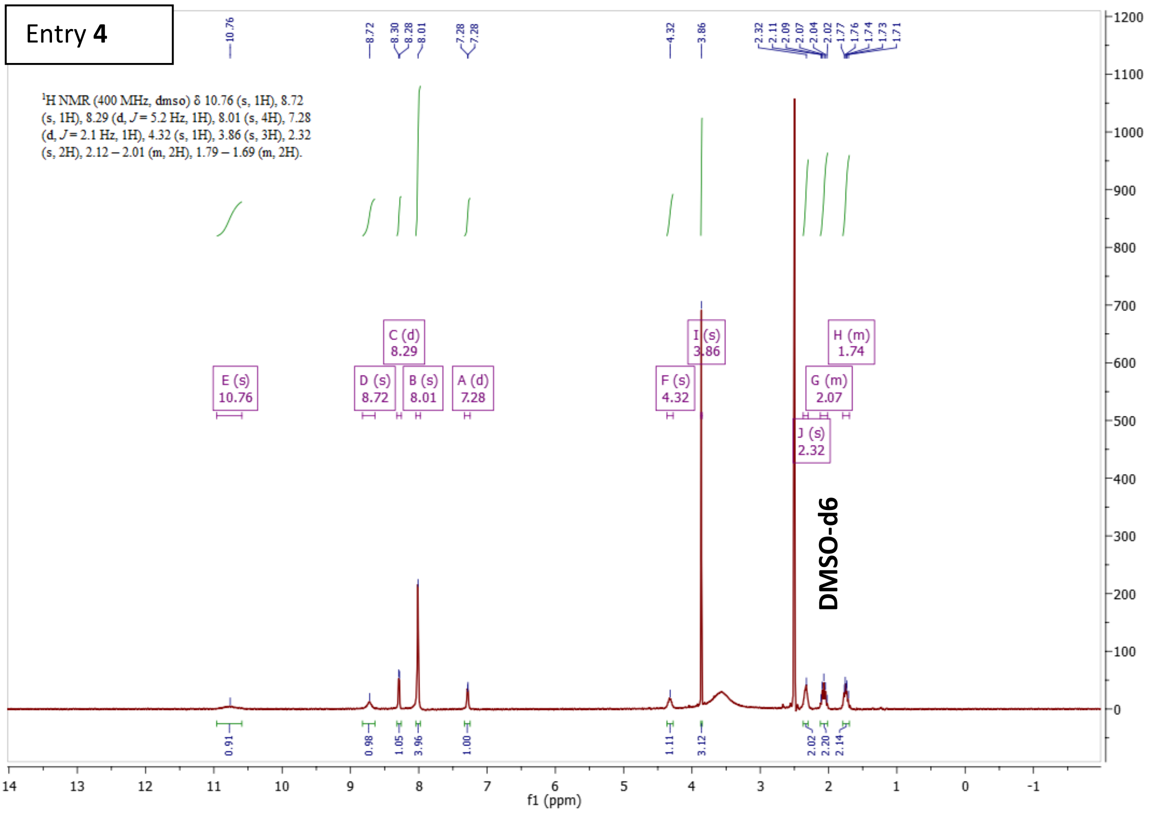

Entry **5**

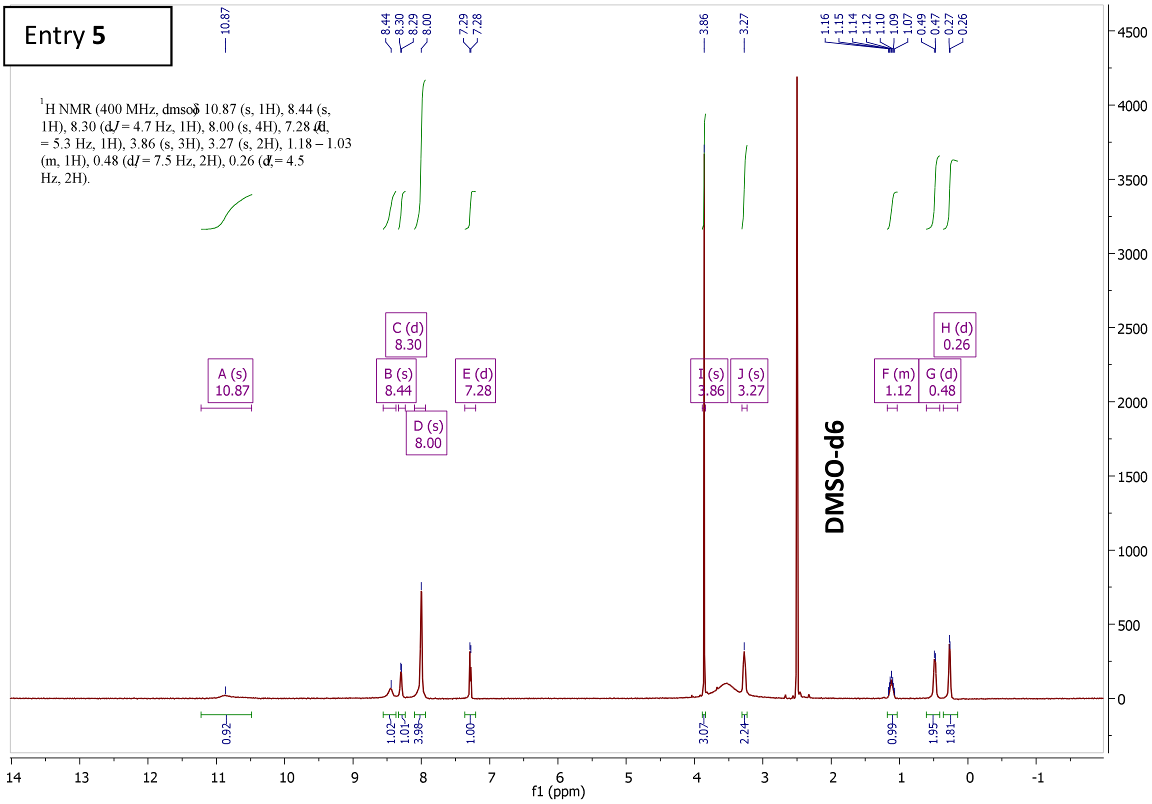

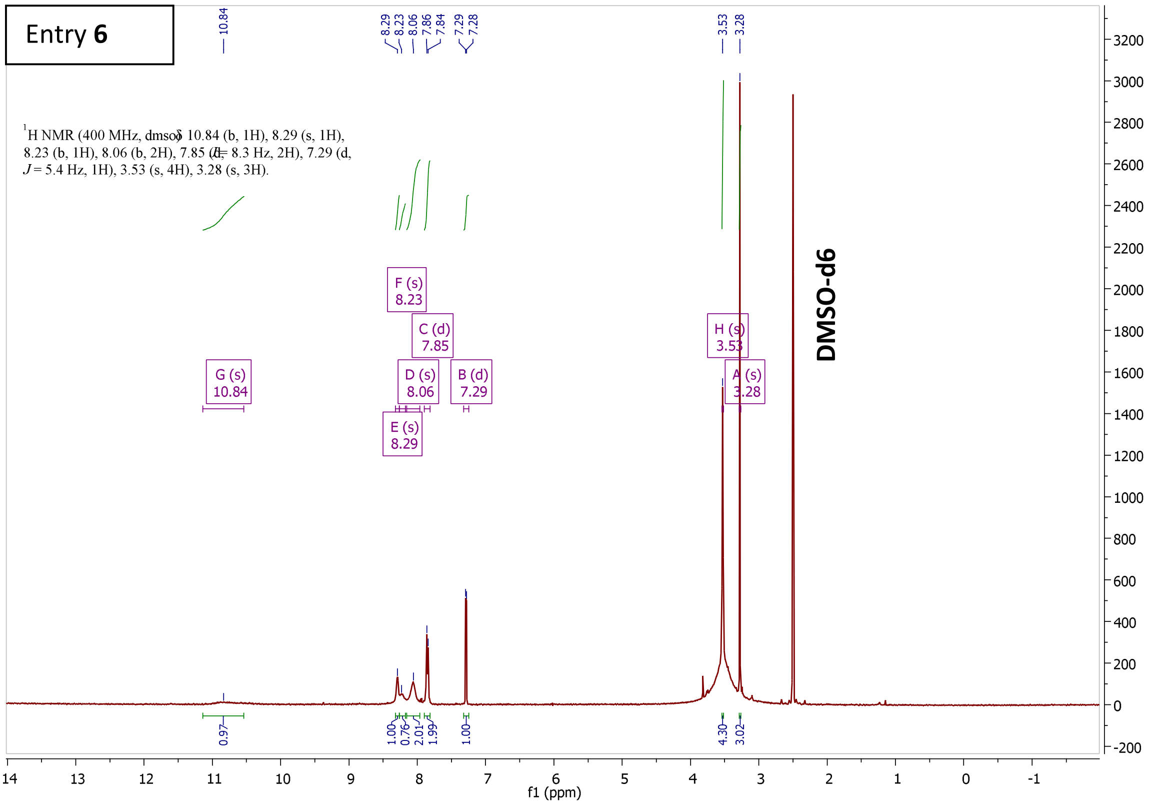
Entry 6
1H NMR (400 MHz, dmso) δ 10.84 (b, 1H), 8.29 (s, 1H), 8.23 (b, 1H), 8.06 (b, 2H), 7.85 (d, J = 8.3 Hz, 2H), 7.29 (d, J = 5.4 Hz, 1H), 3.53 (s, 4H), 3.28 (s, 3H).
DMSO-d6
f1 (ppm)

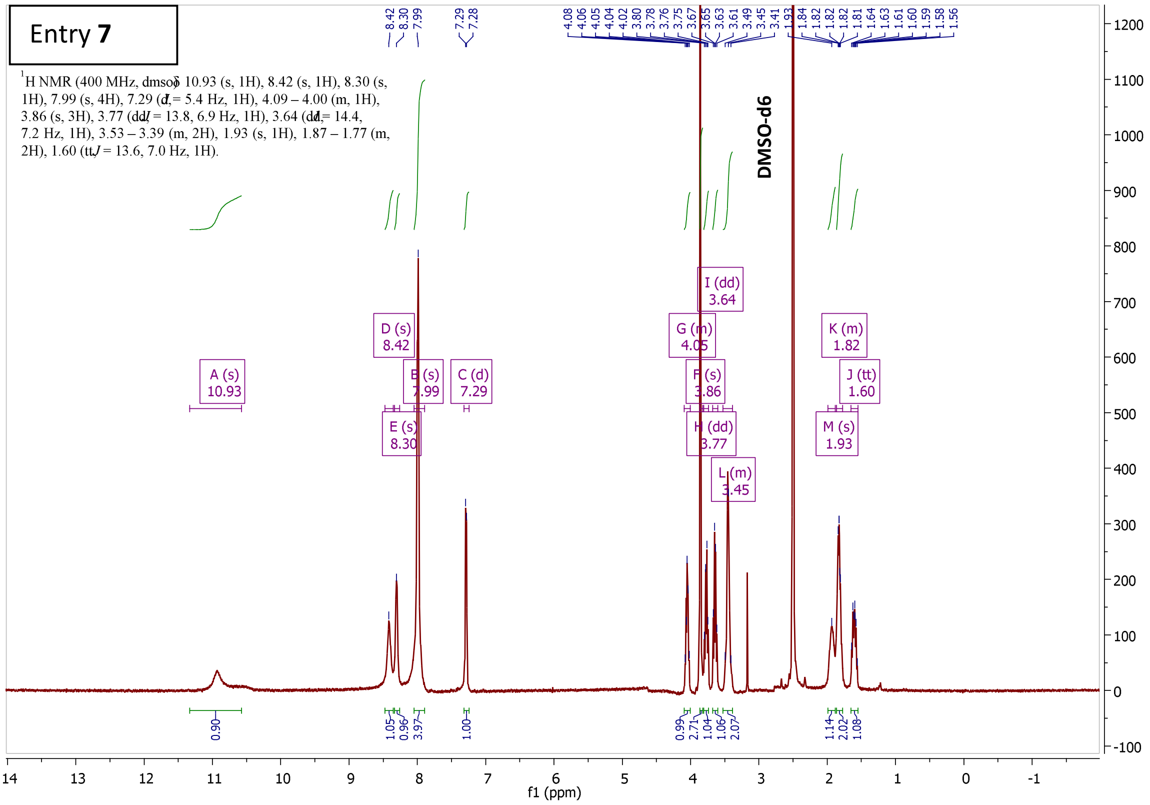
Entry 7
1H NMR (400 MHz, dmso) δ 10.93 (s, 1H), 8.42 (s, 1H), 8.30 (s, 1H), 7.99 (s, 4H), 7.29 (d, J = 5.4 Hz, 1H), 4.09 – 4.00 (m, 1H), 3.86 (s, 3H), 3.77 (dd, J = 13.8, 6.9 Hz, 1H), 3.64 (dd, J = 14.4, 7.2 Hz, 1H), 3.53 – 3.39 (m, 2H), 1.93 (s, 1H), 1.87 – 1.77 (m, 2H), 1.60 (tt, J = 13.6, 7.0 Hz, 1H).
DMSO-d6
f1 (ppm)

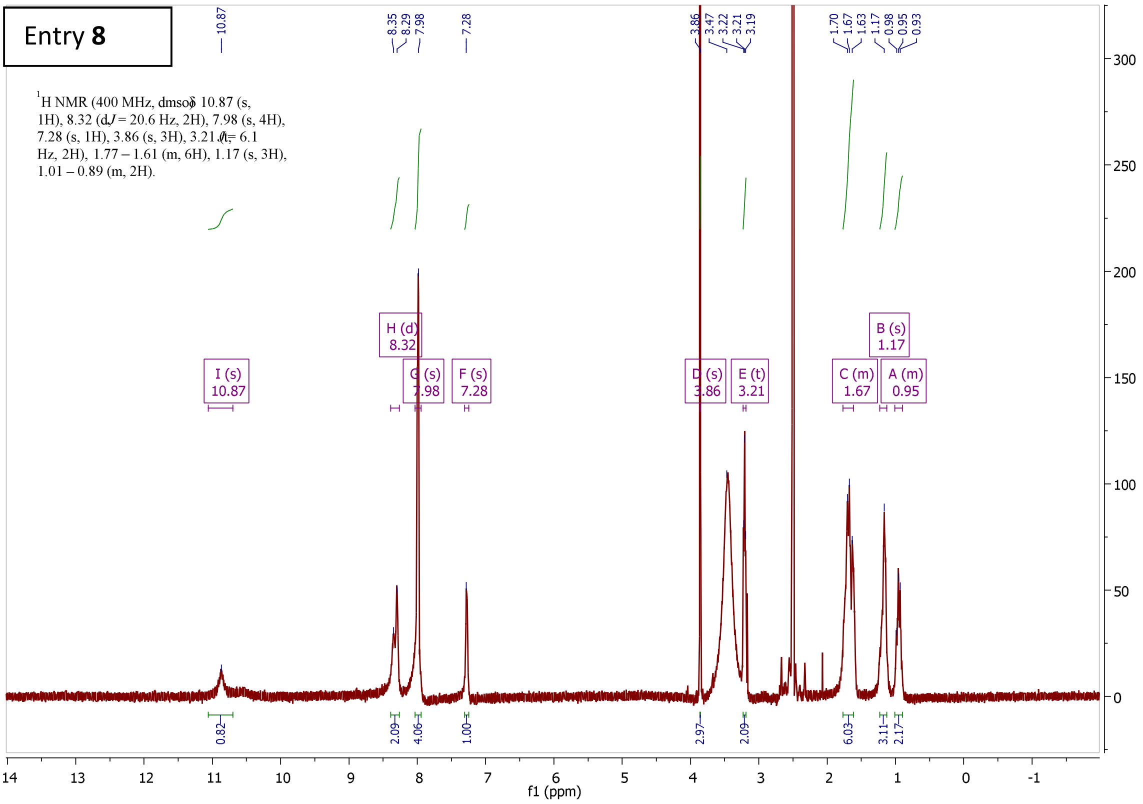
Entry 8
^{1}H NMR (400 MHz, dmso) δ 10.87 (s, 1H), 8.32 (d, J = 20.6 Hz, 2H), 7.98 (s, 4H), 7.28 (s, 1H), 3.86 (s, 3H), 3.21 (t, J = 6.1 Hz, 2H), 1.77 – 1.61 (m, 6H), 1.17 (s, 3H), 1.01 – 0.89 (m, 2H).
I (s) 10.87
H (d) 8.32
G (s) 7.98
F (s) 7.28
D (s) 3.86
E (t) 3.21
C (m) 1.67
B (s) 1.17
A (m) 0.95
f1 (ppm)

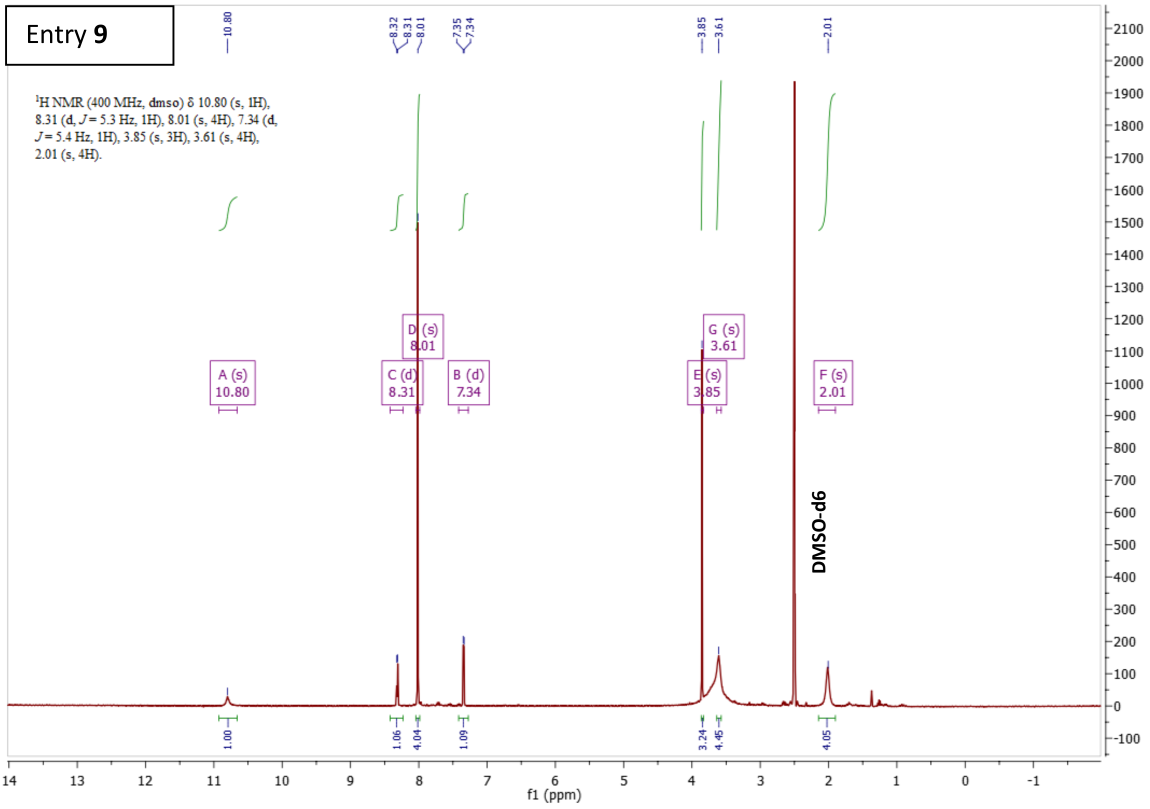
Entry 9
^{1}H NMR (400 MHz, dmso) δ 10.80 (s, 1H), 8.31 (d, J = 5.3 Hz, 1H), 8.01 (s, 4H), 7.34 (d, J = 5.4 Hz, 1H), 3.85 (s, 3H), 3.61 (s, 4H), 2.01 (s, 4H).
A (s) 10.80
C (d) 8.31
D (s) 8.01
B (d) 7.34
E (s) 3.85
G (s) 3.61
F (s) 2.01
DMSO-d6
f1 (ppm)

Entry **10**

^{1}H NMR (400 MHz, dmso) δ 10.67 (s, 1H), 8.27 (d, J = 5.4 Hz, 1H), 8.00 (d, J = 8.7 Hz, 2H), 7.87 (d, J = 8.8 Hz, 2H), 7.30 (d, J = 5.4 Hz, 1H), 3.85 (s, 3H), 3.78 – 3.72 (m, 4H), 1.62 (s, 6H).

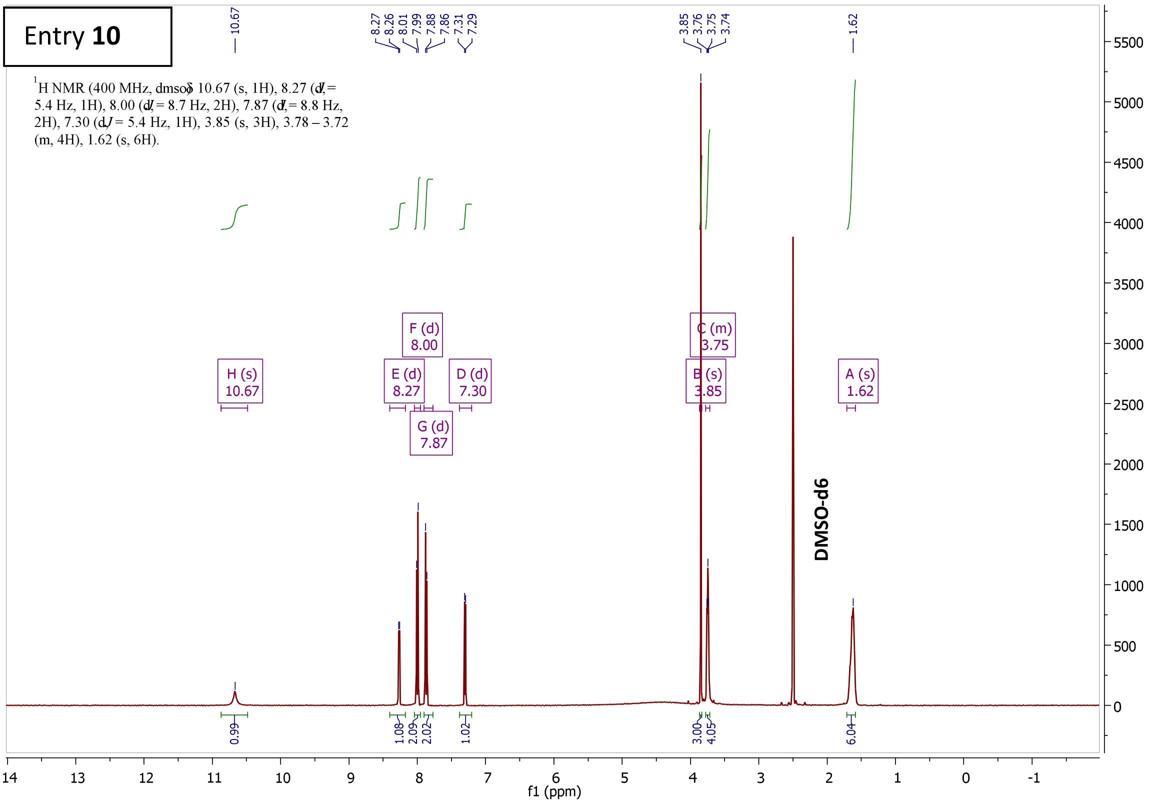

Entry **11**

^{1}H NMR (400 MHz, dmso) δ 10.45 (s, 1H), 8.23 (d, J = 5.4 Hz, 1H), 7.99 (d, J = 8.8 Hz, 2H), 7.89 (d, J = 8.8 Hz, 2H), 7.27 (d, J = 5.4 Hz, 1H), 3.84 (s, 3H), 3.72 (s, 8H).

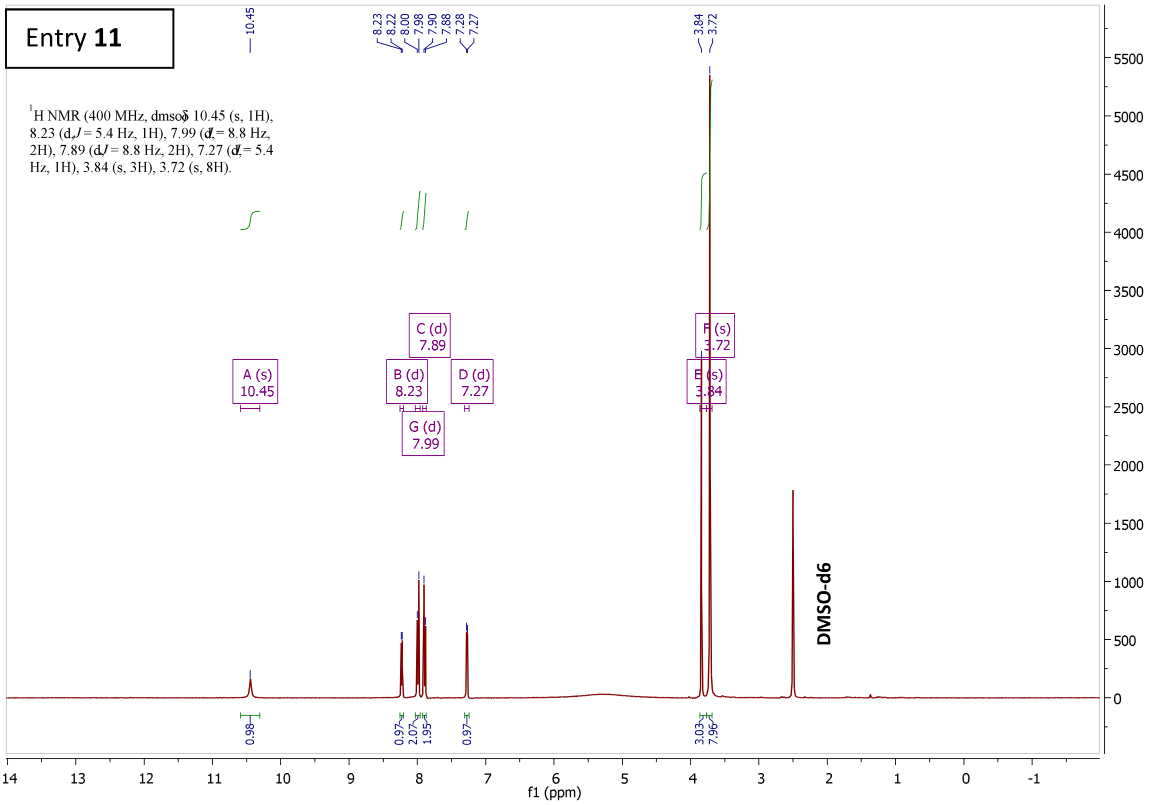

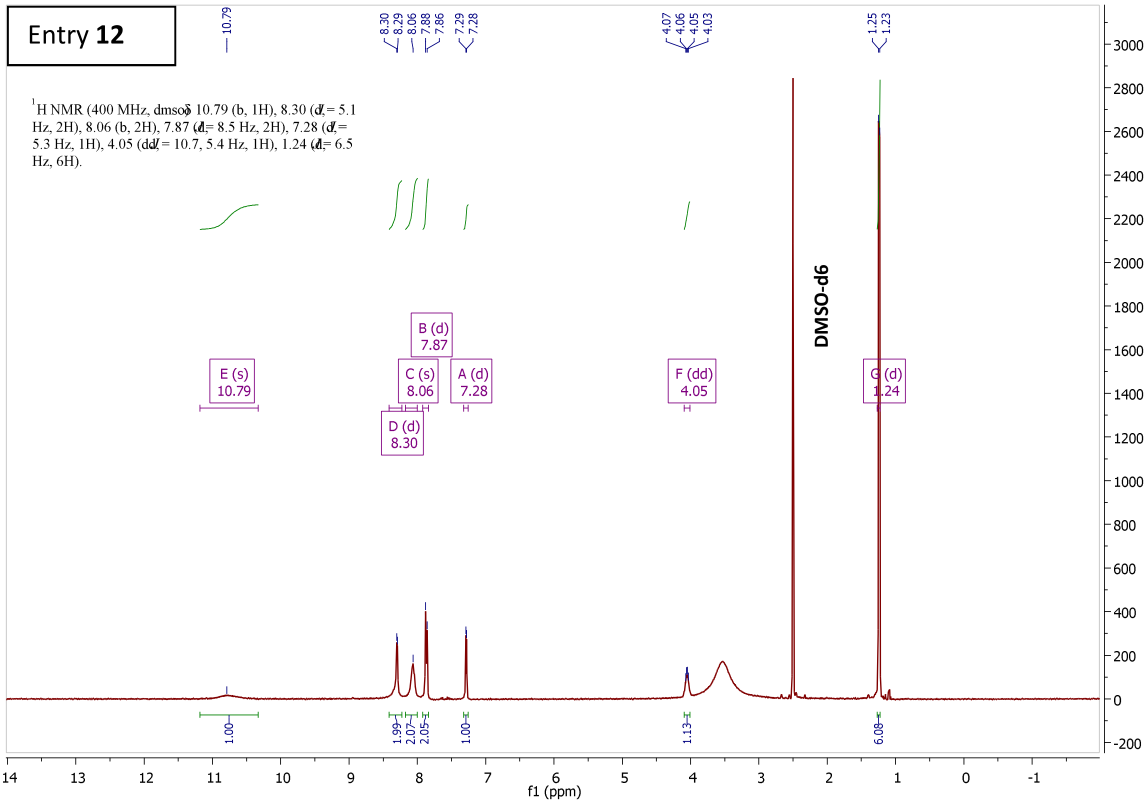
Entry 12
^{1}H NMR (400 MHz, dmso) δ 10.79 (b, 1H), 8.30 (d, J = 5.1 Hz, 2H), 8.06 (b, 2H), 7.87 (d, J = 8.5 Hz, 2H), 7.28 (d, J = 5.3 Hz, 1H), 4.05 (dd, J = 10.7, 5.4 Hz, 1H), 1.24 (d, J = 6.5 Hz, 6H).
DMSO-d6
E (s) 10.79
D (d) 8.30
C (s) 8.06
B (d) 7.87
A (d) 7.28
F (dd) 4.05
G (d) 1.24
f1 (ppm)

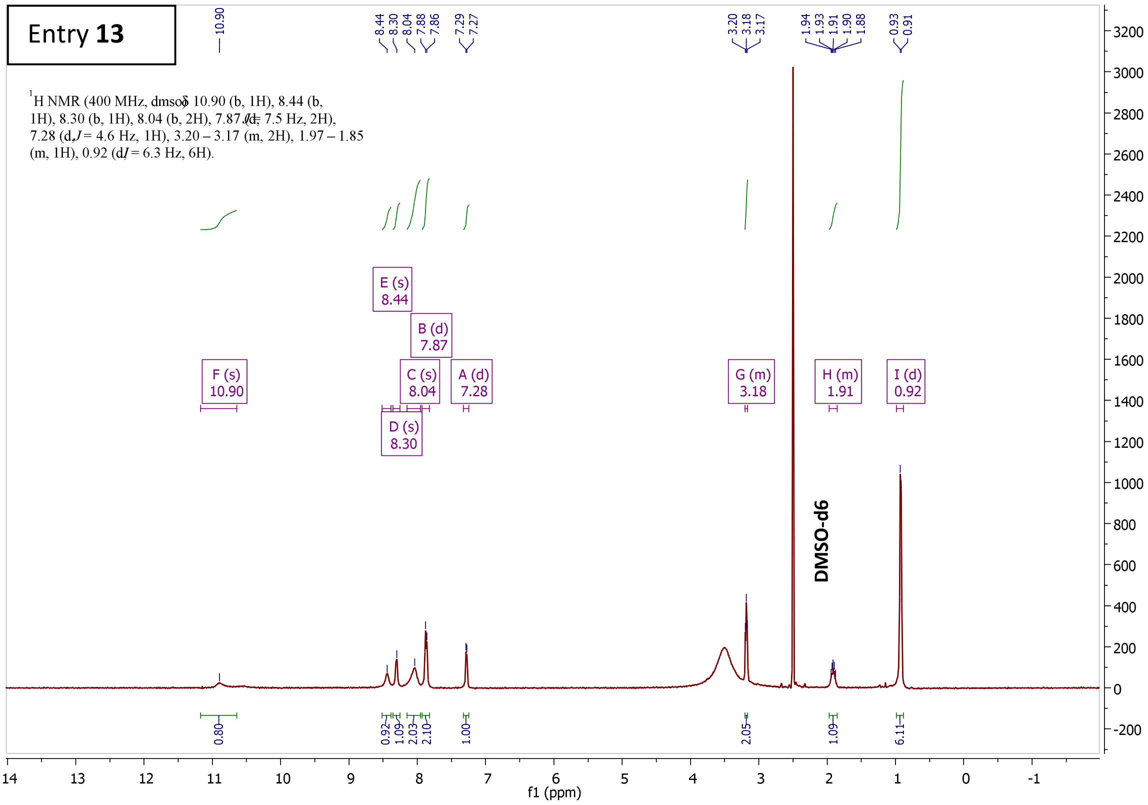
Entry 13
^{1}H NMR (400 MHz, dmso) δ 10.90 (b, 1H), 8.44 (b, 1H), 8.30 (b, 1H), 8.04 (b, 2H), 7.87 (d, J = 7.5 Hz, 2H), 7.28 (d, J = 4.6 Hz, 1H), 3.20 – 3.17 (m, 2H), 1.97 – 1.85 (m, 1H), 0.92 (d, J = 6.3 Hz, 6H).
DMSO-d6
F (s) 10.90
E (s) 8.44
D (s) 8.30
C (s) 8.04
B (d) 7.87
A (d) 7.28
G (m) 3.18
H (m) 1.91
I (d) 0.92
f1 (ppm)

Entry **14**

^{1}H NMR (400 MHz, dmso) δ 10.78 (b, 1H), 8.71 (b, 1H), 8.29 (d, J = 5.2 Hz, 1H), 8.07 (s, 2H), 7.88 (d, J = 7.9 Hz, 2H), 7.28 (d, J = 5.1 Hz, 1H), 4.37 – 4.22 (m, 1H), 2.32 (s, 2H), 2.13 – 1.97 (m, 2H), 1.73 (ddd, J = 18.5, 10.9, 7.7 Hz, 2H).

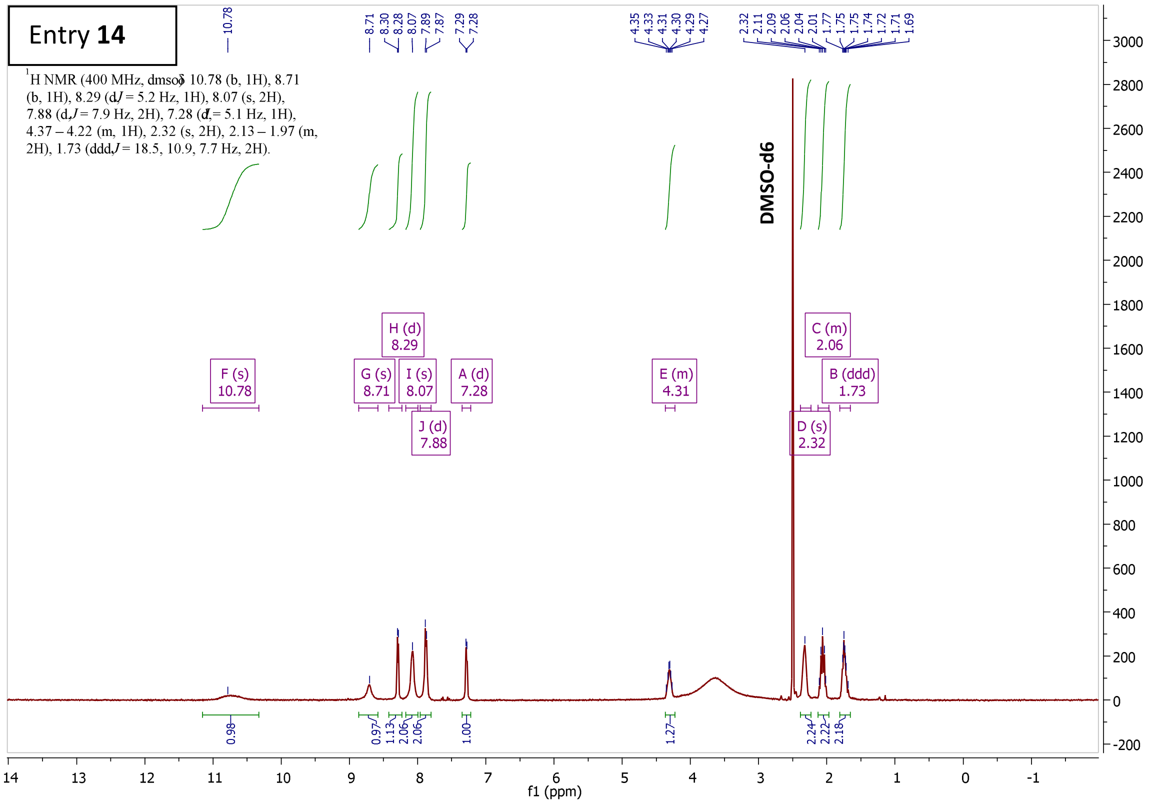

Entry **15**

^{1}H-NMR [600 MHz, DMSO-d6] δ: 8.30 (s, 1H), 8.12 - 8.02 (m, 2H), 7.88 - 7.86 (m, 2H), 7.29 (d, J=5.2, 1H), 3.25 (s, 2H), 1.12 (s, 1H), 0.49 (s, 2H), 0.26 (s, 2H)

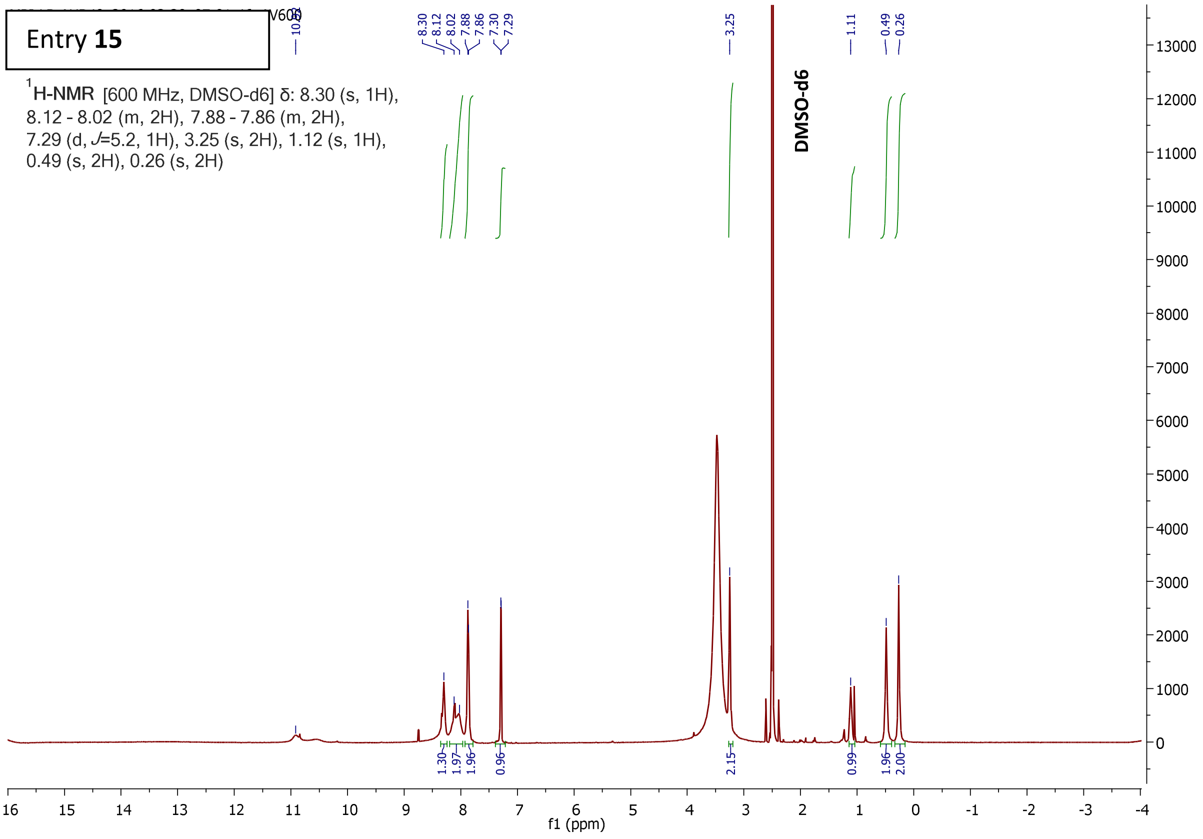

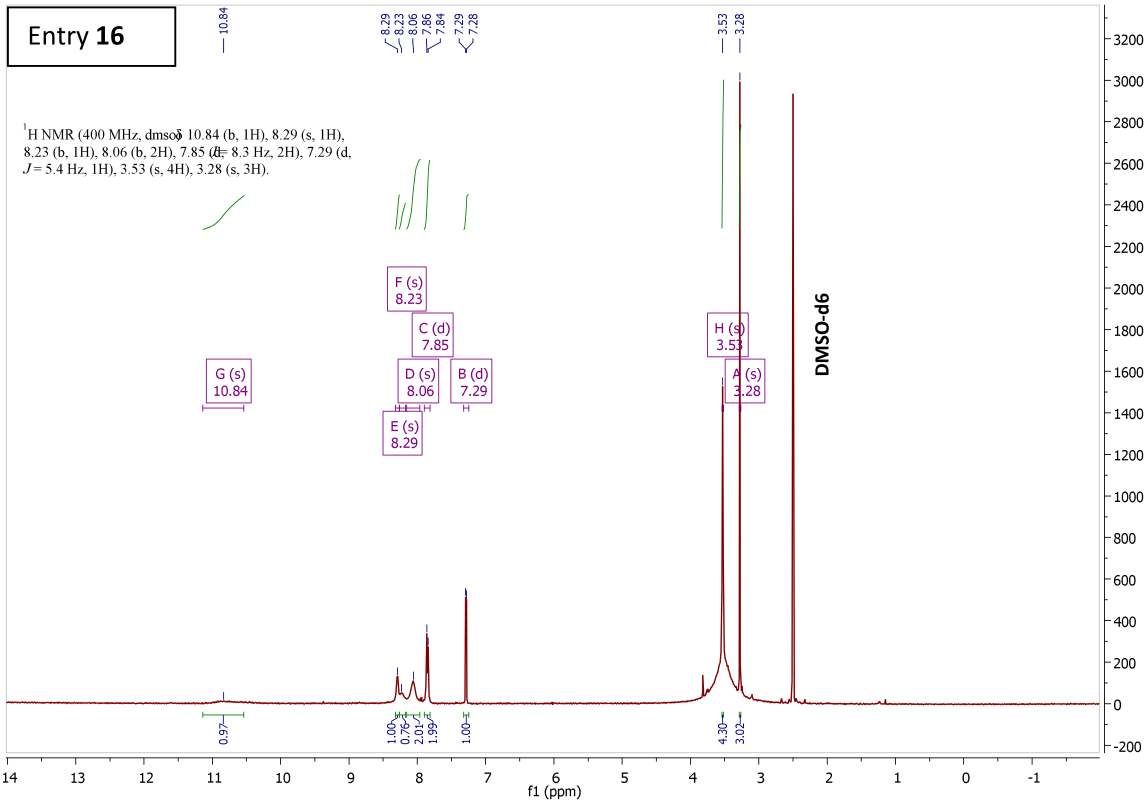

6. References

[1] Sasaki, H.;Hui, C.;Nakafuku, M.; Kondoh, H., A binding site for Gli proteins is essential for HNF-3beta floor plate enhancer activity in transgenics and can respond to Shh in vitro, *Development (Cambridge, England)* **1997**, *124*, 1313–1322.

[2] Taipale, J.;Chen, J. K.;Cooper, M. K.;Wang, B.;Mann, R. K.;Milenkovic, L.;Scott, M. P.; Beachy, P. A., Effects of oncogenic mutations in Smoothened and Patched can be reversed by cyclopamine, *Nature* **2000**, *406*, 1005–1009.

[3] Kremer, L.;Schultz-Fademrecht, C.;Baumann, M.;Habenberger, P.;Choidas, A.;Klebl, B.;Kordes, S.;Scholer, H. R.;Sterneckert, J.;Ziegler, S.;Schneider, G.; Waldmann, H., Discovery of a Novel Inhibitor of the Hedgehog Signaling Pathway through Cell-based Compound Discovery and Target Prediction, *Angewandte Chemie (International ed. in English)* **2017**, *56*, 13021–13025.

[4] Lipinski, R. J.;Gipp, J. J.;Zhang, J.;Doles, J. D.; Bushman, W., Unique and complimentary activities of the Gli transcription factors in Hedgehog signaling, *Experimental cell research* **2006**, *312*, 1925–1938.

[5] Regl, G.;Neill, G. W.;Eichberger, T.;Kasper, M.;Ikram, M. S.;Koller, J.;Hintner, H.;Quinn, A. G.;Frischauf, A.-M.; Aberger, F., Human GLI2 and GLI1 are part of a positive feedback mechanism in Basal Cell Carcinoma, *Oncogene* **2002**, *21*, 5529–5539.

[6] Pfaffl, M. W., A new mathematical model for relative quantification in real-time RT-PCR, *Nucleic acids research* **2001**, *29*, e45.

[7] Schindelin, J.;Rueden, C. T.;Hiner, M. C.; Eliceiri, K. W., The ImageJ ecosystem: An open platform for biomedical image analysis, *Molecular reproduction and development* **2015**, *82*, 518–529.

[8] Rohatgi, R.;Milenkovic, L.; Scott, M. P., Patched1 regulates hedgehog signaling at the primary cilium, *Science (New York, N.Y.)* **2007**, *317*, 372–376.

[9] Sinha, S.; Chen, J. K., Purmorphamine activates the Hedgehog pathway by targeting Smoothened, *Nature chemical biology* **2006**, *2*, 29–30.

[10] Chen, J. K.;Taipale, J.;Cooper, M. K.; Beachy, P. A., Inhibition of Hedgehog signaling by direct binding of cyclopamine to Smoothened, *Genes & development* **2002**, *16*, 2743–2748.

[11] Inoue, T.;Heo, W. D.;Grimley, J. S.;Wandless, T. J.; Meyer, T., An inducible translocation strategy to rapidly activate and inhibit small GTPase signaling pathways, *Nature methods* **2005**, *2*, 415–418.

[12] Hammond, G. R. V.;Machner, M. P.; Balla, T., A novel probe for phosphatidylinositol 4-phosphate reveals multiple pools beyond the Golgi, *The Journal of cell biology* **2014**, *205*, 113–126.

[13] Hsu, P. D.;Lander, E. S.; Zhang, F., Development and Applications of CRISPR-Cas9 for Genome Engineering, *Cell* **2014**, *157*, 1262-1278.

[14] Bauer, D. E.;Canver, M. C.; Orkin, S. H., Generation of Genomic Deletions in Mammalian Cell Lines via CRISPR/Cas9, *Jove-J Vis Exp* **2015**.

[15] Wen, X.;Lai, C. K.;Evangelista, M.;Hongo, J.-A.;Sauvage, F. J. d.; Scales, S. J., Kinetics of hedgehog-dependent full-length Gli3 accumulation in primary cilia and subsequent degradation, *Molecular and cellular biology* **2010**, *30*, 1910–1922.

[16] Cox, J.; Mann, M., MaxQuant enables high peptide identification rates, individualized p.p.b.-range mass accuracies and proteome-wide protein quantification, *Nature biotechnology* **2008**, *26*, 1367–1372.

[17] Tyanova, S.;Temu, T.;Sinitcyn, P.;Carlson, A.;Hein, M. Y.;Geiger, T.;Mann, M.; Cox, J., The Perseus computational platform for comprehensive analysis of (prote)omics data, *Nature methods* **2016**, *13*, 731–740.

[18] M. J. Waring, D. M. Andrews, P. F. Faulder, V. Flemington, J. C. McKelvie, S. Maman, M. Preston, P. Raubo, G. R. Robb, K. Roberts, R. Rowlinson, J. M. Smith, M. E. Swarbrick, I. Treinies, J. J. G. Winter, R. J. Wood, *Chemical communications (Cambridge, England)* **2014**, *50*, 5388;.

[19] A. Freitag, P. Prajwal, A. Shymanets, C. Harteneck, B. Nurnberg, C. Schachtele, M. Kubbutat, F. Totzke, S. A. Laufer, *Journal of medicinal chemistry* **2015**, *58*, 212.
